# Hyperisampsins H–M, Cytotoxic Polycyclic Polyprenylated Acylphloroglucinols from *Hypericum sampsonii*

**DOI:** 10.1038/srep14772

**Published:** 2015-10-06

**Authors:** Hucheng Zhu, Chunmei Chen, Qingyi Tong, Xintao Chen, Jing Yang, Junjun Liu, Bin Sun, Jianping Wang, Guangmin Yao, Zengwei Luo, Yongbo Xue, Yonghui Zhang

**Affiliations:** 1 Hubei Key Laboratory of Natural Medicinal Chemistry and Resource Evaluation, School of Pharmacy, Tongji Medical College, Huazhong University of Science and Technology, Wuhan 430030, China; 2 State Key Laboratory of Phytochemistry and Plant Resources in West China, Kunming Institute of Botany, Chinese Academy of Sciences, Kunming 650204, China

## Abstract

Six new polycyclic polyprenylated acylphloroglucinols (PPAPs), named hyperisampsins H–M (**1**–**6**), were isolated from the aerial parts of *Hypericum sampsonii*, together with five known analogs (**7**–**11**). The structures of **1**–**6** were established by extensive spectroscopic analyses, including HRESIMS and NMR. In addition, the absolute configurations of these new compounds were determined by electronic circular dichroism (ECD) calculations. Compounds **1** and **2** represent the first examples of PPAPs possessing a unique *γ*-lactone ring at C-23, while **3**–**6** differed from normal PPAPs with an unprecedented 1,2-dioxane ring. Compounds **1**–**7** were evaluated for their cytotoxic activities against a panel of human cancer cell lines *in vitro*, of which **3**, **4**, and **6** exhibited significant cytotoxic activities with IC_50_ values ranging from 0.56 to 3.00 *μ*M. Moreover, compound **3** induces leukemia cell apoptotic death, evidenced by activation of caspase-3, degradation of PARP, up-regulation of Bax, and down-regulation of Bcl-2 and Bcl-xl.

Polycyclic polyprenylated acylphloroglucinols (PPAPs) are a class of secondary metabolites that usually possess bicyclo[3.3.1]nonane-2,4,9-trione, adamantyl, or homoadamantyl-like core structures and associated with various bioactivities, such as anti-tumors, anti-bacterial, and anti-HIV^1^. Recently, studies on naturally occurring PPAPs and on the total synthesis of complex PPAPs have attracted growing interest due to their challenging structures and potential biological activities^1^,^2^,^3^,^4^,^5^,^6^. Plants of the genus *Hypericum* are well known as rich sources of PPAPs, and a large number of PPAPs have been discovered and reported as the main components of this genus^1^. *Hypericum sampsonii* is a traditional Chinese medicine that is used to treat backache, burns, diarrhea, and swelling. Over the past two decades, phytochemical investigations conducted by several groups have resulted in the isolation and characterization of more than 50 PPAPs with various carbon skeletons and bioactivities from this plant^7^,^8^,^9^,^10^,^11^,^12^,^13^,^14^,^15^,^16^,^17^,^18^.

As part of our continuing efforts to discover bioactive PPAPs in the genus *Hypericum*, a series of PPAPs and other components were previously identified^19^,^20^,^21^,^22^,^23^. In our current study, further chemical investigation of *H. sampsonii* has led to the isolation of six new PPAPs, termed hyperisampsins H–M (**1**–**6**), together with five known analogs (**7**–**11**). Compounds **1** and **2** represent the first examples of PPAPs possessing a rare *γ*-lactone ring at C-23, while **3**–**6** differed from normal PPAPs with an unprecedented 1,2-dioxane ring. Here, we report the isolation, structural elucidation, and absolute configuration determination of these new PPAPs. In addition, the cytotoxic activities of compounds **1**–**7** against five human cancer cell lines were evaluated, of which **3**, **4**, and **6** exhibited significant cytotoxic activities, with IC_50_ values ranging from 0.56 to 3.00 *μ*M. The apoptosis-inducing potential of compound **3** and the mechanism of action were further studied by FACS and western blotting, and the results showed that it induced apoptosis in leukemia cells, mediated by activation of caspase-3, degradation of PARP, upregulation of Bax, and downregulation of Bcl-2 and Bcl-xl.

## Results and Discussion

### Isolation and Structure Elucidation

**Figure 1:**
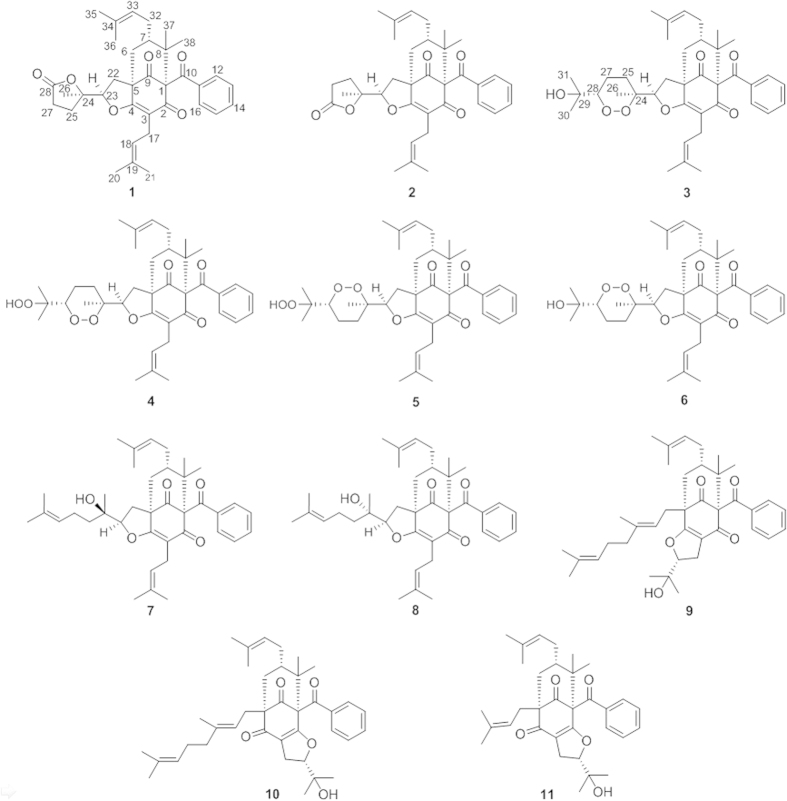
Structures of isolated compounds.

A 95% EtOH extract of the aerial parts of *H. sampsonii* was suspended in water and successively partitioned with petroleum ether and CHCl_3_. The petroleum ether and CHCl_3_ extracts were then subjected to a series of chromatographic separations, including silica gel, RP-C_18_ (reverse-phase), Sephadex LH-20, and semipreparative HPLC to yield six new (**1**–**6**) and five known (**7**–**11**) PPAPs (Fig. 1). The structures of the known compounds were elucidated by comparing their NMR data with those reported in the literature, and they were identified as attenuatumione C (**7**)^24^, sampsonione K (**8**)^10^, otogirinin D (**9**)^25^, and sampsoniones M (**10**)^10^ and N (**11**)^11^.

Hyperisampsin H (**1**), a colorless oil, had a molecular formula of C_35_H_42_O_6_ based on ^13^C NMR data and on a quasi-molecular ion peak at *m*/*z* 581.2799 [M + Na]^+^ in HRESIMS that required 15 indices of hydrogen deficiency. The ^1^H NMR (Table 1) spectrum of **1** showed resonances for a monosubstituted phenyl group (*δ*_H_ 7.43, 2H, d, *J* = 8.2 Hz; 7.38, 1H, t, *J* = 7.3 Hz; and 7.27, 2H, t, *J* = 7.6 Hz), two olefinic protons (*δ*_H_ 4.95, 1H, t, *J* = 7.2 Hz and 4.88, 1H, t, *J* = 7.1 Hz), an oxygenated methine (*δ*_H_ 4.72, 1H, dd, *J* = 10.3, 6.1 Hz), and seven methyl singlets (*δ*_H_ 1.40, 1.45, 1.48, 1.54, 1.56, 1.58, and 1.69). The ^13^C NMR and DEPT (Table 2) of **1** revealed 35 carbons, which resolved into three carbonyls, one ester carbonyl, a monosubstituted benzene ring, six olefinic carbons, four quaternary carbons (one oxygenated), two methines (one oxygenated), six methylenes, and seven methyls. These characteristic NMR data suggested that **1** was a PPAP derivative related to sampsonione K (**8**)^10^.

**Figure 2:**
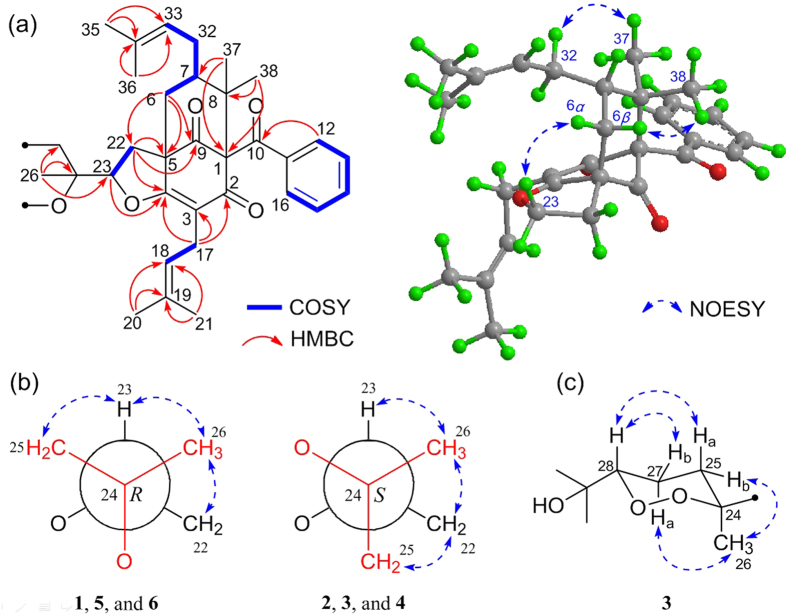
(**a**) Selected 2D NMR correlations for the core structures of **1**–**6**; (**b**) Newman projections for C-24/C-23 of **1**–**6**; (**c**) key NOESY correlations for the 1,2-dioxane ring of **3**.

A side-by-side comparison of the ^1^H and ^13^C NMR data of **1** with those of **8** along with a tracing of the connectivities observed in the HMBC and ^1^H–^1^H COSY spectra (Fig. 2) revealed that both **1** and **8** have the same bicyclo[3.3.1]nonane core structure. However, compound **1** differs from **8** by the absence of two olefinic carbons and two methyl groups, which, together with the presence of an unexpected carboxyl group at *δ*_C_ 175.5 on the side chain at C-23, imply that **1** is an oxidative degradation product of **8**^10^. This conclusion was supported by the ^1^H–^1^H COSY correlations of H-25/H-27 and H-22/H-23 and by the long-range HMBC interactions from H-23 to C-4, H-26 to C-23, C-24, and C-25, and from H-25 to C-23, C-27, and C-28. In addition, with 14 indices of hydrogen deficiency occupied by the tricyclic core, a phenyl group, three carbonyls, and three double bonds, the remaining index indicated the presence of an unprecedented *γ*-lactone ring between C-28 (*δ*_C_ 175.5) and C-24 (*δ*_C_ 85.7).

**Figure 3:**
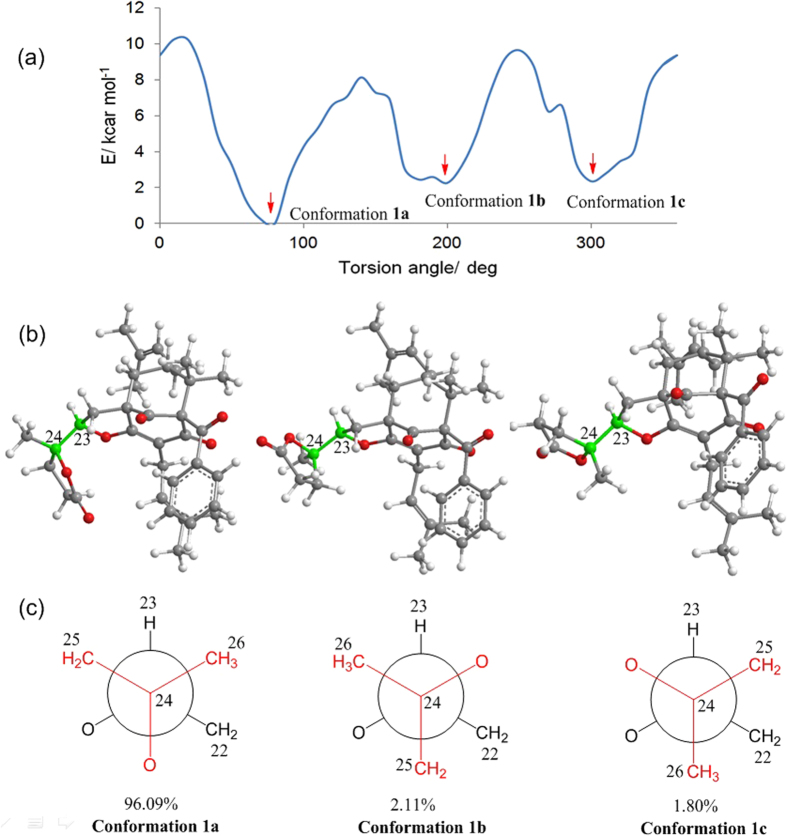
(**a**) Energy profiles displayed for the torsion angle of C-23/C-24 of compound **1**; (**b**) Three stable conformations of compound **1** showing the rotation of C-23/C-24; (**c**) Abbreviated Newman drawing of C-23/C-24 of **1**.

The relative configurations at C-1, C-5, and C-7 of the core skeleton of **1** were established through a NOESY experiment (Fig. 2) and was further confirmed by comparing its ^1^H and ^13^C NMR data (Tables 1 and 2) with those of sampsonione K (**8**)^10^. The NOESY correlation between Me-38 and H-6*β* revealed their cofacial and axial relationship. Therefore, the large coupling constant of H-6*β*/H-7 (*J*_*6β*/7_ = 7.3 Hz) and the absence of coupling between H-6*α* and H-7 indicated that H-7 was equatorial and *β* oriented. These analyses were also supported by the literature^1^, which indicated that when the isoprenyl at C-7 is in an axial position, the deviation of the chemical shifts of the two protons attached to C-6 should be in the range of 0.0–0.2 ppm, while the chemical shift of C-7 should be in the range of 45–49 ppm^1^. With compound **1**, the deviation of the chemical shifts of H-6*α* and H-6*β* (Δ*δ*_H_ = 0.1 ppm), along with that of C-7 (*δ*_C_ 47.6), corresponded well with the above conditions, confirming that the isoprenyl at C-7 was in the axial orientation. Furthermore, the *α*-configuration of H-23 was determined by the NOESY correlation of H-23/H-6*α*, which was identical to that of **8**^10^. To determine the configuration of C-24, the rotation energy barrier of the single bond C-23/C-24 was calculated (Fig. 3a)^26^. As the literature reported, rotational energy barrier of 20 kcal mol^–1^ was a threshold to distinguish between atropisomers and non-atropisomers^26^, therefore, theoretically, the carbon-carbon bond between C-23 and C-24 could rotate to certain extent. However, the calculated Boltzmann distribution of the most stable conformation of **1** (conformation **1a**) could reach up to 96.09% (Fig. 3), which together with the observed NOESY correlations (Fig. 2b) of Me-26 with H-23 and H-22 and of H-23/H-25 indicated that this rotation was restricted^27^, and suggested a R* configuration for C-24. Thus, the structure of **1** was elucidated as shown, and it represents the first example of a PPAP with a unique *γ*-lactone ring.

The molecular formula of hyperisampsin I (**2**) was identical to that of **1** as revealed by the HRESIMS spectrum (*m/z* 581.2798, [M + Na]^+^). The structural elucidation of **2** was straightforward, as a comparison of its ^1^H and ^13^C NMR data (Tables 1 and 2) with those of **1**, suggested that **1** and **2** shared great structural similarity. Careful analysis of the 2D NMR (^1^H–^1^H COSY and HMBC) spectra of **2** suggested that the planar structure of **2** was identical to that of **1**. The relative configurtions at C-1, C-5, and C-7 were established to be identical to those of **1** in the same mannar as described for **1**. In additon, the NOESY correlation of H-23/H-6*α* suggested that the H-23 of **2** was also *α*-oriented. Therefore, the only difference between **2** and **1** was the relative configuration of C-24, which was revealed by the NOESY cross-peaks (Fig. 2b) of Me-26 with H-22 and H-23 and of H-22 with H-25. The relative configuration of C-24 of **2** was thereby elucidated as S*, and compound **2** was revealed as the C-24 epimer of **1**.

Hyperisampsin J (**3**) was isolated as a colorless oil. A molecular formula of C_38_H_50_O_7_ was elucidated by the HRESIMS spectrum, indicating 14 indices of hydrogen deficiency. Extensive comparison of the ^1^H and ^13^C NMR (Tables 1 and 2) data of **3** with those of **8** suggested that the structure of **3** resembled that of **8**^10^ except for the absence of the double bond at C-28 and C-29 in **8** and the presence of two oxygenated carbons (*δ*_C_ 87.0 and 72.0) in **3**. Careful analysis of the HMBC and ^1^H–^1^H COSY spectra of **3** revealed that it possessed the same carbon connectivities as **8**, albeit with significantly higher degrees of oxidation. Considering the chemical shifts of C-24 (*δ*_C_ 80.9) and C-28 (*δ*_C_ 87.0) in **3**, along with the 14 indices of hydrogen deficiency required by its HRESIMS, it was reasonable to locate a 1,2-dioxane ring at C-23 via a peroxide linkage between C-24 and C-28^13^, which was also supported by the TLC detection colorated with KI-starch (Figure S1). This deduction was further confirmed by a comparison of the ^13^C NMR chemical shift values of this fragment (sequence from C-24 to C-28) in **3** with those of the 1,2-dioxane ring reported in the literature^28^. Thus, the planar structure of **3** was elucidated.

The relative configurations of C-1, C-5, C-7, and C-23 in **3** were established to be the same as those of **1** by careful analyses of the ^1^H and ^13^C NMR spectra and the NOESY spectra (Fig. 2). Considering the distributions of **1** based on the former conformation analyses, the S* configuration of C-24 in **3** was determined by NOESY correlations from Me-26 to H-23 and H-22 and from H-22 to H-25. Molecular modeling depicted the 1,2-dioxane ring to be in a chair conformation containing an axially oriented methyl group (Me-26) and an equatorially orientated carbon-carbon single bond at C-24/C-23. Additionally, the NOESY (in C_5_D_5_N, Fig. 2c) correlations from H-27a to H-25b and Me-26 and from H-28 to H-25a and H-27b, together with the large coupling constant of H-28/H-27a (*J* = 10.6 Hz), revealed that H-28 was axial and *β*-oriented. Therefore, the relative configuration of **3** was established, and it appears to be the first example of PPAPs possessing an unexpected 1,2-dioxane ring.

**Figure 4:**
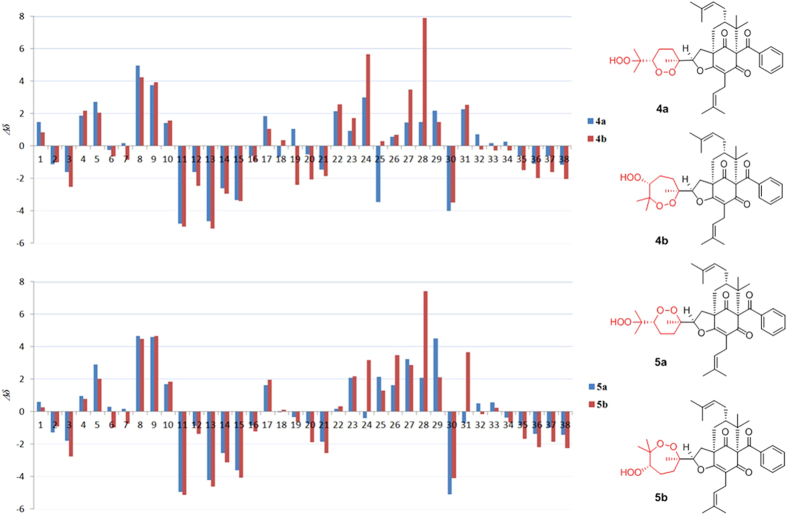
Calculated ^13^C NMR data for 4 and 5.

Detailed inspection of the ^1^H and ^13^C NMR spectroscopic data (Tables 1 and 2) of compounds **4**–**6** indicated that their structures closely resembled that of **3**. Comprehensive analyses of the HMBC, ^1^H–^1^H COSY, and HRESIMS spectra of **4**–**6** suggested that compound **6** has the same planar structure as that of **3**, while **4** and **5** might have structures with 1,2-dioxane rings or 1,2-dioxepane rings (**4a**/**4b** and **5a**/**5b**, Fig. 4). In the end, the complete structures of **4** and **5** were determined to be **4a** and **5a** by comparing the experimental ^13^C NMR data with those calculated for **4a**, **4b**, **5a**, and **5b** (Fig. 4). The relative configurtions at C-1, C-5, C-7, and C-23 of **4**–**6** were identical to those of **3** as revealed by their NOESY experiments and coupling constants. Consequently, the NOESY correlations from Me-26 to H-23 and H-22 and from H-23 to H-25 in **5** suggested an R* configuration of C-24. As with **5**, the relative configurations of C-24 in compounds **4** and **6** were also established by NOESY experiments. Similar to **3**, the orientations of H-28 in **4**–**6** were all set in axial orientations, owing to the large coupling constants of H-28 (9.7–11.0 Hz). Thus, the structures of **4**–**6** were established and named as hyperisampsins K–M.

**Figure 5:**
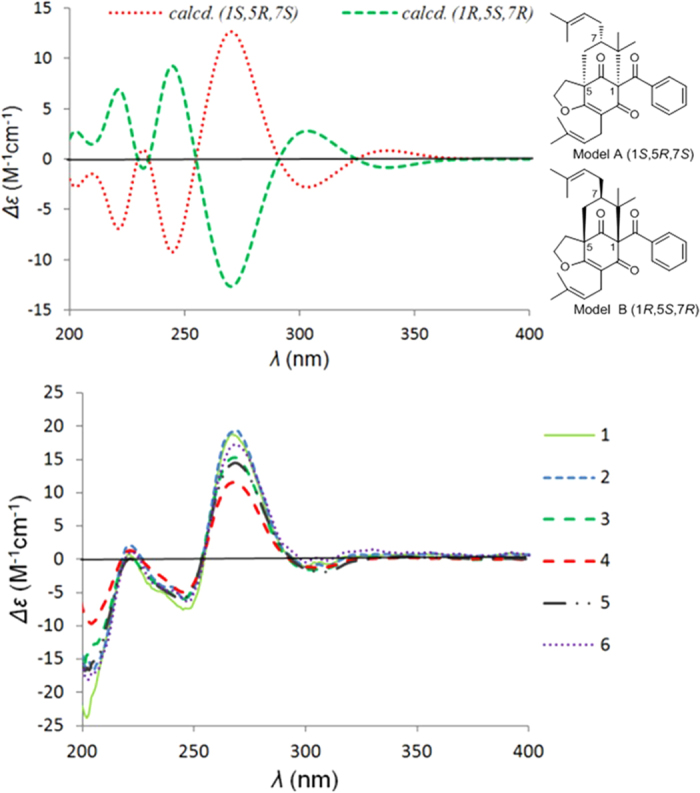
Calculated ECD for models (A,B) and the experimental ECD spectra of 1–6.

Comparison of the electronic circular dichroism (ECD) spectra (Fig. 5) of the closely related compounds **1**–**6** suggested that all of them had a strong positive Cotton effect (CE) at *λ*_max_ 270 nm and two negative CEs at *λ*_max_ 246 and 305 nm. To determine the absolute configurations of **1**–**6**, the ECD spectra of two simplified models A and B (Fig. 5) were calculated using time-dependent density functional theory (TDDFT) with Gaussian 09. The ECD spectra of **1**–**6** were subsequently compared with the calculated ECD curves of models A (1*S*,5*R*,7*S*) and B (1*R*,5*S*,7*R*) (Fig. 5), which revealed a good agreement between the experimental curves and the calculated curve of model A (1*S*,5*R*,7*S*). In addition, the experimental ECD curves of **1**–**6** were closely similar to those of hyperattenins A and B^21^, suggesting the same absolute configurations of the core structures. Thus, the absolute configurations of **1**–**6** were determined as shown.

### Cytotoxic Activities Evaluation

Compounds **1**–**7** were tested for their cytotoxic activities against five human tumor cell lines, including a myeloid leukemia line (HL-60 cells), a hepatocellular carcinoma line (SMMC-7721), a lung carcinoma line (A-549), a breast cancer line (MCF-7), and a colon cancer line (SW480). Cis-platin (DDP) was used as positive control for antitumor activity (Table 3)^19^. Among the compounds tested, compounds **3**, **4**, and **6** exhibited the most potent cytotoxicity, with IC_50_ values ranging from 0.56 to 3.00 *μ*M. Compounds **5** and **7** showed moderate cytotoxic activities, with IC_50_ values over a range of 3.03–25.92 *μ*M.

### Flow cytometry analysis of cell apoptosis

**Figure 6:**
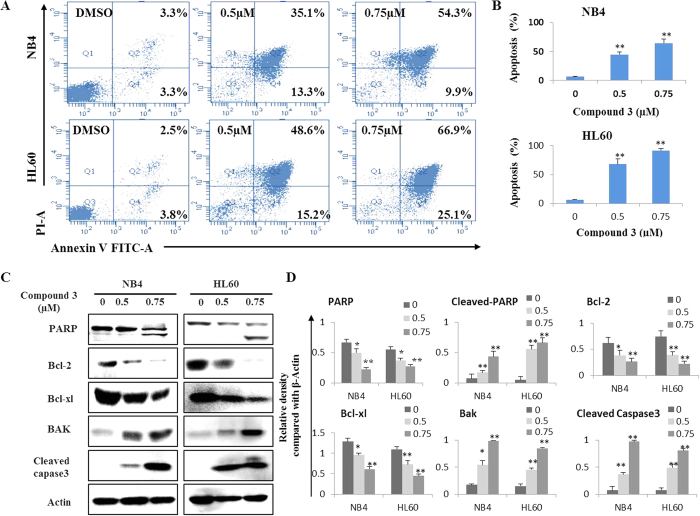
Compound 3 induces apoptosis in leukemia cells. (A) After 48 h treatment, cell apoptosis was determined by Annexin V-FITC and PI staining using flow cytometric analysis. Cells in the lower right quadrant indicate early apoptotic cells, and cells in the upper right quadrant indicate late apoptotic cells. (**B**) Columns, means of three different experiments; bars, SD, *P < 0.05, **P < 0.01 vs control group. (**C**) Western blot analysis for PARP, Bcl-2, Bcl-xl, Bak and caspase-3 levels, *β*-Actin was used as a loading control. (**D**) The relative density compared with *β*-Actin of each protein was detected by Image J, data are presented as the means of three experiments, bars, SD, *P < 0.05, **P < 0.01 vs control group.

To analyze the potential cell apoptosis induced by these cytotoxic PPAPs, two acute myeloid leukemia cell lines (HL60 and NB4) were treated with compound **3** for 48 hours. As show in Fig. 6(A,B), treated HL60 and NB4 cells underwent dramatic cellular apoptosis in a concentration-dependent manner. Compared with the control group, the 0.75 *μ*M compound **3** resulted in about 64.2% and 92.0% apoptosis incidence in NB4 and HL60 cells, respectively.

### Western blot analysis of apoptosis related proteins

Caspase 3 activation is responsible for the proteolytic degradation of PARP, a hallmark of cells undergoing apoptosis. Bcl-2 family also plays a central regulatory role in the mitochondrial pathway of apoptosis. The balance between Bak verses Bcl-2 and Bcl-xl is important for apoptotic induction^29^. As shown in Fig. 6(C,D), compound **3** treatments activatedthe expression levels of Caspase 3 and PARP.It also up-regulated Bak, but down-regulated Bcl-2 and Bcl-xl. In conclusion, these data suggest that compound **3**-induced apoptosis was mediated by the activation of caspase-3, upregulation of Bax, downregulation of Bcl-2/Bcl-xl, and degradation of PARP.

PPAPs are a special class of phloroglucinol derivatives, which have attracted great interest from both chemistry and pharmacology communities, since the report of the first natural occurring adamantyl derivative (plukenetione A) in 1996^30^. Recently, many bioactive PPAPs with complex and intriguing skeletons were reported, such as hyperuralones A and B^31^, hypersubones A and B^32^, and hyperisampsins A–D^19^. In this study, six new PPAPs (**1**–**6**), possessing novel unique *γ*-lactone or 1,2-dioxane rings, were isolated from the aerial parts of *Hypericum sampsonii*. Compounds **3**, **4**, and **6** exhibited significant cytotoxic activities with IC_50_ values ranging from 0.56 to 3.00 *μ*M. Moreover, we have demonstrated that compound **3** has the capacity to induce cell apoptotic death in leukemia cells, revealing, for the first time, the mechanism of PPAP-mediated cytotoxicity, which may attract more attentions from synthesis chemistry and pharmacology communities. In conclusion, the novel structure of **3** combined with its significant cytotoxic activities reported in this study may greatly promote the anti-tumor studies of PPAPs, and further investigations on the mechanism and structure-function relationship for developing more excellent agent are necessary.

## Methods

### General experimental procedures

Optical rotations were determined with a Perkin-Elmer 341 polarimeter. UV, ECD, and FT-IR spectra were measured using a Varian Cary 50, a JASCO-810 spectrometer, and a Bruker Vertex 70, respectively. NMR spectra were recorded on a Bruker AM-400 spectrometer, and the ^1^H and ^13^C NMR chemical shifts were referenced to the solvent or solvent impurity peaks for CDCl_3_ (*δ*_H_ 7.26 and *δ*_C_ 77.0) and pyridine-*d*_5_ (*δ*_H_ 7.19 and *δ*_C_ 123.5). High-resolution electrospray ionization mass spectra (HRESIMS) were obtained in the positive ion mode with a Thermo Fisher LC-LTQ-Orbitrap XL spectrometer. Semi-preparative HPLC was performed on an Agilent 1200 quaternary system with a UV detector or on a Dionex HPLC system equipped with an Ultimate 3000 pump, an Ultimate 3000 autosampler injector, and an Ultimate 3000 diode array detector (DAD) controlled by Chromeleon software (version 6.80) using a reverse-phase C_18_ column (5 *μ*m, 10 × 250 mm, Welch Ultimate XB-C_18_). Column chromatography was performed using silica gel (100–200 and 200–300 mesh; Qingdao Marine Chemical Inc., China), ODS (50 *μ*m, Merck, Germany), Sephadex LH-20 (Merck, Germany), or MCI gel (75–150 *μ*m, Merck, Germany). Thin-layer chromatography (TLC) was performed with silica gel 60 F_254_ (Yantai Chemical Industry Research Institute) and RP-C_18_ F_254_ plates (Merck, Germany).

### Plant material

The aerial parts of *H. sampsonii* were collected from the Da-bie Mountain areas of Hubei Province, P. R. China, in October 2011, and were identified by Professor Jianping Wang. A voucher specimen (ID 20111008) has been deposited with the Herbarium of Materia Medica, Faculty of Pharmacy, Tongji Medical College of Huazhong University of Science and Technology, China.

### Extraction and isolation

The air-dried aerial parts of *H. sampsonii* (50 kg) were extracted with 95% EtOH, and the extract was partitioned with petroleum ether and CHCl_3_ against water. The petroleum ether soluble extract (800 g) was separated by chromatography on a silica gel column (5 kg, 20 × 120 cm; petroleum ether to acetone, 100:0 → 0:100) to furnish eight fractions (Fr. 1–Fr. 8). Fr. 3 was further purified by column chromatography (silica gel CC, 1 kg, 10 × 100 cm), eluting with a gradient of petroleum ether in isopropyl alcohol to yield three subfractions (Fr. 3.1–Fr. 3.3). Fr. 3.2 was separated over silica gel (petroleum ether to acetone, 20:1 → 5:1) to obtain six parts (Fr. 3.2.1–Fr. 3.2.6). These parts were subjected to Sephadex LH-20 (MeOH) and ODS (MeOH-H_2_O) and were then purified by semi-preparative HPLC. Compounds **7** (8 mg) and **10** (10 mg) were obtained from Fr. 3.2.2; Compounds **8** (10 mg) and **11** (5 mg) were purified from Fr. 3.2.3; Compound **9** (17 mg) was isolated from Fr. 3.2.3. Fr. 6 was applied to ODS (MeOH to H_2_O, 50% → 100%) to obtain six subfractions (Fr. 6.1–Fr. 6.6). Fr. 6.3 was separated with Sephadex LH-20 (MeOH) and ODS (MeOH to H_2_O, 60% → 100%) to obtain three mixtures (III–V). Mixture III was purified by semi-preparative HPLC (CH_3_CN-H_2_O, 88%) to obtain **3** (15 mg). Fr. 6.4 was further separated by Sephadex LH-20 (MeOH) and ODS (MeOH to H_2_O, 70% → 100%) and was then purified by semi-preparative HPLC (CH_3_CN-H_2_O, 75%) to obtain **1** (8 mg), **2** (9 mg), and **6** (5 mg).

The CHCl_3_ soluble extract (approximately 1 kg) was also separated by chromatography on silica gel (6 kg, 20 × 120 cm; petroleum ether to acetone, 100:0 → 0:100) to furnish six parts (A–F). Fraction B was subjected to silica gel CC and was eluted with petroleum ether to acetone (50:1 → 1:1) to obtain seven subfractions (B.1–B.7). Subfraction B.2 was further subjected to Sephadex LH-20 (MeOH) and then MPLC (ODS, MeOH to H_2_O, from 40% to 100%) to obtain six additional subfractions (B.2.1–B.2.6). B.2.3 was further purified by Sephadex LH-20 (MeOH), silica gel CC, and finally by semi-preparative HPLC (CH_3_OH-H_2_O, 90%) to obtain compounds **4** (15 mg) and **5** (9 mg).

#### Hyperisampsin H (**1**)

Colorless oil, 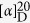 + 4 (*c* = 0.2, CHCl_3_); UV (CH_3_OH) *λ*_max_ (log *ε*) = 204 (4.29), 248 (4.01), and 274 (3.90) nm; ECD (MeOH) *λ*_max_ (Δ*ε*) 221 (+0.7), 247 (−7.4), 267 (+18.8), 300 (−1.1) nm; IR *ν*_max_ = 1780, 1729, 1695, and 1627 cm^−1^; for ^1^H NMR (400 MHz) and ^13^C NMR (100 MHz) data see Tables 1 and 2, respectively; HRESIMS *m/z* 581.2799 [M + Na]^+^ (calculated for C_35_H_42_O_6_Na, 581.2879).

#### Hyperisampsin I (**2**)

Colorless oil, 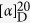 + 42 (*c* = 0.2, CHCl_3_); UV (CH_3_OH) *λ*_max_ (log *ε*) = 204 (4.35), 248 (4.08), and 275 (3.98) nm; ECD (MeOH) *λ*_max_ (Δ*ε*) 221 (+2.0), 245 (−6.0), 269 (+19.3), 300 (−1.2) nm; IR *ν*_max_ = 1780, 1728, 1696, and 1627 cm^−1^; for ^1^H NMR (400 MHz) and ^13^C NMR (100 MHz) data see Tables 1 and 2, respectively; HRESIMS *m/z* 581.2798 [M + Na]^+^ (calculated for C_35_H_42_O_6_Na, 581.2879).

#### Hyperisampsin J (**3**)

Colorless oil, 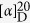 + 21 (*c* = 0.3, CH_3_OH); UV (CH_3_OH) *λ*_max_ (log *ε*) = 205 (4.46), 247 (4.17), and 277 (4.15) nm; ECD (MeOH) *λ*_max_ (Δ*ε*) 221 (+0.3), 245 (−6.0), 268 (+15.3), 302 (−1.6) nm; IR *ν*_max_ = 3518, 1728, 1698, and 1627 cm^−1^; for ^1^H NMR (400 MHz) and ^13^C NMR (100 MHz) data see Tables 1 and 2, respectively; HRESIMS *m/z* 641.3429 [M + Na]^+^ (calculated for C_38_H_50_O_7_Na, 641.3454).

#### Hyperisampsin K (**4**)

Colorless oil, 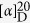 + 12 (*c* = 0.4, CHCl_3_); UV (CH_3_OH) *λ*_max_ (log *ε*) = 203 (4.25), 247 (4.00), and 274 (3.88) nm; ECD (MeOH) *λ*_max_ (Δ*ε*) 221 (+1.3), 246 (−5.0), 268 (+11.6), 307 (−1.2) nm; IR *ν*_max_ = 3419, 1734, and 1700 cm^−1^; for ^1^H NMR (400 MHz) and ^13^C NMR (100 MHz) data see Tables 1 and 2, respectively; HRESIMS *m/z* 657.3380 [M + Na]^+^ (calculated for C_38_H_50_O_8_Na, 657.3403).

#### Hyperisampsin L (**5**)

Colorless oil, 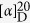 + 9 (*c* = 0.1, CH_3_OH); UV (CH_3_OH) *λ*_max_ (log *ε*) = 203 (4.39), 248 (4.04), and 277 (3.95) nm; ECD (MeOH) *λ*_max_ (Δ*ε*) 221 (+0.1), 244 (−5.9), 268 (+14.5), 307 (−1.9) nm; IR *ν*_max_ = 3423, 1728, 1697, and 1626 cm^−1^; for ^1^H NMR (400 MHz) and ^13^C NMR (100 MHz) data see Tables 1 and 2, respectively; HRESIMS *m/z* 657.3383 [M + Na]^+^ (calculated for C_38_H_50_O_8_Na, 657.3403).

#### Hyperisampsin M (**6**)

Colorless oil, 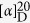 + 47 (*c* = 0.1, CHCl_3_); UV (CH_3_OH) *λ*_max_ (log *ε*) = 204 (4.22), 248 (3.95), and 275 (3.88) nm; ECD (MeOH) *λ*_max_ (Δ*ε*) 221 (+1.0), 247 (–6.3), 267 (+17.3), 300 (−0.4) nm; IR *ν*_max_ = 3421, 1722, 1696, and 1626 cm^−1^; for ^1^H NMR (400 MHz) and ^13^C NMR (100 MHz) data see Tables 1 and 2, respectively; HRESIMS *m/z* 641.3374 [M + Na]^+^ (calculated for C_38_H_50_O_7_Na, 641.3454).

### Computational details

The theoretical calculations of compound **1** and the simplified models (A and B) of compounds **1**–**6** were performed using Gaussian 09. Conformational analysis was initially performed using Maestro in Schrödinger 2010 conformational searching together with the OPLS_2005 molecular mechanics methods. The optimized conformation geometries and thermodynamic parameters of all conformations were provided. The OPLS_2005 conformers were optimized at the B3LYP/6–31G(d, p) level. The theoretical calculation of ECD was performed using time-dependent density functional theory (TDDFT) at the B3LYP/6–31G(d, p) level in methanol with a PCM model. The calculated ECD curve was generated using SpecDis 1.51^33^. *R*_vel_ was used in this work. The 3D structures of **4a**/**4b** and **5a**/**5b**, generated by Chem3D, were optimized in chloroform by using Gaussian 09 at the B3LYP/6–31G* level. Both optimized structures were then further used as the input structures for NMR calculations. For each conformation, the NMR calculation was performed using Gaussian 09 at the B3LYP/6–31G* level. Finally, the relative errors between the computed and recorded ^13^C NMR spectra were calculated^34^.

### Cytotoxic assay

Five human cancer cell lines (HL-60, SMMC-7721, A-549, MCF-7, and SW-480), together with one noncancerous cell line, the Beas-2B human bronchial epithelial cell line, were used in the cytotoxic activity assay. Cytotoxic activity was measured as described in our previous report^19^.

### Flow cytometry analysis of cell apoptosis

Apoptosis analysis was carried out using an apoptosis detection kit (Keygen, Nanjing, China) according to the manufacturer’s instructions. Briefly, HL60 and NB4 cells were exposed to vehicle (DMSO < 0.01%) and compound **3** (0.5 and 0.75 *μ*M) for 48 h, then cells were collected and washed with cold PBS, and then resuspended in 500 *μ*L binding buffer. After that, 5 *μ*L of AnnexinV-FITC and 10 *μ*L of PI were added. After supravital staining, cell apoptosis was analyzed by flow cytometry (Becton Dickinson, San Jose, CA, USA).

### Western blot analysis

Western blot analysis was conducted as described previously (citation). Briefly, cells were treated with DMSO and compound **3** (0.5 and 0.75 *μ*M) for 48 h, and then lysed in a radio immune-precipitation assay buffer. Protein concentrations were determined using a BCA protein assay kit (Byontime, Beijing, China). Samples were subjected to electrophoresis in 10% SDS-PAGE gels followed by transfer to PVDF membrane and probed with specific antibodies, including PARP, Bcl-2, BCL-XL, Bak, Cleaved Caspase 3, and *β*-Actin (Cell Signaling Technology, Inc.). Blots bands were visualized using the horseradish peroxidase conjugated secondary antibodies and chemiluminescent substrate.

## Additional Information

**How to cite this article**: Zhu, H. *et al.* Hyperisampsins H–M, Cytotoxic Polycyclic Polyprenylated Acylphloroglucinols from *Hypericum sampsonii*. *Sci. Rep.* **5**, 14772; doi: 10.1038/srep14772 (2015).

## Supplementary Material

Supplementary Information

## Figures and Tables

**Table 1:** ^1^H NMR Data for Compounds 1–6 in CDCl_3_ (400 MHz, *J* in Hz).

*no.*	1	2	3	4	5	6
6*α*	2.29 d (14.1)	2.34 d (14.1)	2.30 d (14.1)	2.30 d (14.2)	2.36 d (14.1)	2.31 d (14.1)
6*β*	2.19 dd (14.1, 7.3)	2.18 dd (14.1, 7.5)	2.16 m	2.16 m	2.22 m	2.18 m
7	1.50 m	1.51 m	1.50 m	1.48 m	1.56 m	1.51 m
12	7.43 d (8.2)	7.44 d (8.2)	7.45 d (7.4)	7.45 d (8.0)	7.51 d (7.3)	7.46 d (8.5)
13	7.27 t (7.6)	7.23 t (7.8)	7.21 t (7.8)	7.22 t (7.8)	7.27 t (7.8)	7.21 t (7.8)
14	7.38 t (7.3)	7.39 t (7.4)	7.38 t (7.4)	7.38 t (7.4)	7.44 t (7.4)	7.38 t (7.4)
15	7.27 t (7.6)	7.23 t (7.8)	7.21 t (7.8)	7.22 t (7.8)	7.27 t (7.8)	7.21 t (7.8)
16	7.43 d (8.2)	7.44 d (8.2)	7.45 d (7.4)	7.45 d (8.0)	7.51 d (7.3)	7.46 d (8.5)
17*a*	3.09 dd (14.2, 7.9)	3.07 dd (14.2, 7.0)	3.08 dd (13.9, 7.4)	3.08 dd (13.9, 7.4)	3.13 dd (14.2, 7.5)	3.08 dd (13.9, 7.7)
17*b*	2.91 m	2.97 dd (14.2, 7.6)	3.00 dd (13.9, 7.8)	3.00 dd (13.9, 7.9)	3.05 dd (14.2, 7.6)	3.01 dd (13.9, 7.7)
18	4.95 t (7.2)	4.94 t (7.0)	5.07 t (7.6)	5.07 t (7.5)	5.11 t (7.5)	5.05 t (7.7)
20	1.58 s	1.60 s	1.61 s	1.61 s	1.65 s	1.60 s
21	1.56 s	1.60 s	1.63 s	1.63 s	1.66 s	1.61 s
22*a*	2.88 m	2.46 dd (13.3, 11.1)	2.63 dd (13.3, 11.0)	2.64 dd (13.3, 10.8)	2.65 dd (13.3, 10.9)	2.58 dd (13.3, 11.0)
22*b*	1.91 dd (13.3, 6.1)	1.92 dd (13.3, 5.8)	1.86 dd (13.3, 5.8)	1.86 m	1.90 m	1.86 dd (13.3, 6.1)
23	4.72 dd (10.3, 6.1)	4.88 dd (11.1, 5.8)	4.67 dd (11.0, 5.9)	4.65 dd (10.8, 5.8)	4.78 m	4.75 m
25*a*	2.54 dd (13.1, 2.2)	2.14 m	1.88 m	1.88 m	1.88 m	1.81 m
25*b*	2.16 m	2.01 dt (13.2, 8.4)	1.81 m	1.78 m	1.72 m	1.66 m
26	1.45 s	1.47 s	1.35 s	1.37 s	1.37 s	1.31 s
27*a*	2.79 m	2.69 t (8.4)	1.97 m	1.87 m	1.90 m	1.96 m
27*b*	2.66 m		1.82 m		1.62 m	1.81 m
28			3.91 dd (10.6, 3.2)	4.27 dd (9.7, 3.2)	4.29 dd (10.8, 3.2)	3.88 dd (11.0, 3.5)
30			1.23 s	1.27 s	1.32 s	1.23 s
31			1.27 s	1.28 s	1.38 s	1.28 s
32	2.16 m	2.14 m	2.16 m	2.16 m	2.22 m	2.18 m
33	4.88 t (7.1)	4.87 t (7.5)	4.87 t (7.2)	4.87 t (7.2)	4.93 t (7.2)	4.87 t (7.1)
35	1.69 s	1.69 s	1.69 s	1.69 s	1.66 s	1.69 s
36	1.54 s	1.53 s	1.54 s	1.53 s	1.75 s	1.54 s
37	1.48 s	1.48 s	1.48 s	1.47 s	1.59 s	1.49 s
38	1.40 s	1.41 s	1.40 s	1.40 s	1.45 s	1.40 s

**Table 2:** ^13^C NMR Data for Compounds 1–6 in CDCl_3_ (100 MHz).

*no.*	1	2	3	4	5	6	*no.*	1	2	3	4	5	6
1	76.9	77.2	77.2	77.2	77.7	77.1	20	25.6	25.8	25.8	25.8	25.8	25.8
2	193.8	193.7	193.8	193.9	193.8	193.7	21	17.7	17.7	17.8	17.8	17.8	17.8
3	116.0	116.2	115.8	115.8	115.8	115.8	22	31.1	31.8	31.0	30.9	31.0	31.1
4	171.4	171.5	172.2	172.2	172.6	172.2	23	88.3	87.0	87.1	87.1	86.8	86.7
5	58.4	58.4	58.2	58.2	58.2	58.2	24	84.1	85.7	80.9	80.9	81.5	81.7
6	36.9	36.4	36.5	36.5	36.7	36.6	25	31.3	27.7	27.5	27.5	27.5	28.1
7	47.6	47.6	47.7	47.7	47.7	47.7	26	23.1	23.1	17.3	17.4	15.4	15.3
8	49.2	49.4	49.3	49.3	49.2	49.2	27	28.9	28.6	19.2	19.4	19.4	18.9
9	204.9	205.1	205.3	205.8	205.4	205.5	28	175.5	175.3	87.0	84.4	84.3	86.9
10	193.3	193.2	193.4	193.4	193.4	193.4	29			72.0	83.1	83.0	72.1
11	136.5	136.9	136.9	136.9	136.9	136.8	30			24.9	20.6	20.6	24.7
12	128.1	128.0	128.1	128.1	128.1	128.1	31			26.2	21.1	21.1	25.8
13	128.1	127.9	127.9	127.9	127.9	127.9	32	29.0	28.9	29.0	28.9	28.9	28.9
14	132.1	132.1	131.9	132.0	132.0	132.0	33	124.4	124.0	124.3	124.3	124.3	124.2
15	128.1	127.9	127.9	127.9	127.9	127.9	34	132.8	133.2	132.9	133.0	133.0	133.0
16	128.1	128.0	128.1	128.1	128.1	128.1	35	25.8	25.8	25.8	25.8	25.8	26.1
17	22.4	22.3	22.4	22.4	22.3	22.3	36	17.7	17.8	17.7	17.7	17.8	17.8
18	119.0	119.2	119.6	119.5	119.4	119.4	37	22.2	22.2	22.3	22.2	22.3	22.3
19	133.4	133.2	132.6	132.6	132.9	132.8	38	26.9	26.9	27.0	26.9	26.9	26.9

**Table 3:** Cytotoxic Activities of 1–7 (IC_50_ in *μ*M).

Compounds	HL-60	SMMC-7721	A-549	MCF-7	SW480	BEAS-2B
1	>40.00	>40.00	>40.00	>40.00	>40.00	–
2	>40.00	>40.00	>40.00	>40.00	>40.00	–
3^a^	0.56	0.58	0.53	0.88	2.49	1.50
4	1.67	2.15	2.13	2.73	3.00	2.71
5	3.03	11.30	11.13	11.54	13.59	15.77
6	1.42	2.28	1.89	1.66	2.90	3.04
7	15.52	18.36	15.19	5.72	20.10	17.08
DDP^b^	1.17	6.43	9.24	15.86	13.42	11.11

^a^The IC_50_ value of compound **3** against NB4 cell was 0.63 *μ*M.

^b^DDP (*cis*-platin) was used as a positive control; “–” not tested.

## References

[b1] Ciochina R. & Grossman R. B. Polycyclic polyprenylated acylphloroglucinols. Chem. Rev. 106, 3963–3986 (2006). 16967926 10.1021/cr0500582

[b2] Richard J. A., Pouwer R. H. & Chen D. Y. K. The chemistry of the polycyclic polyprenylated acylphloroglucinols. Angew. Chem. Int. Ed. 51, 4536–4561 (2012). 10.1002/anie.201103873 22461155

[b3] Singh I. P., Sidana J., Bharate S. B. & Foley W. J. Phloroglucinol compounds of natural origin: Synthetic aspects. Nat. Prod. Rep. 27, 393–416 (2010). 20179878 10.1039/b914364p

[b4] Boyce J. H. & Porco J. A. Jr. Asymmetric, stereodivergent synthesis of (–)-clusianone utilizing a biomimetic cationic cyclization. Angew. Chem. Int. Ed. 53, 7832–7837 (2014). 10.1002/anie.201404437 PMC4182949 24916169

[b5] Bellavance G. & Barriault L. Total syntheses of hyperforin and papuaforins A–C, and formal synthesis of nemorosone through a gold(I)-catalyzed carbocyclization. Angew. Chem. Int. Ed. 53, 6701–6704 (2014). 10.1002/anie.201403939 24838522

[b6] Grenning A. J., Boyce J. H. & Porco J. Jr. Rapid synthesis of polyprenylated acylphloroglucinol analogs via dearomative conjunctive allylic annulation. A. J. Am. Chem. Soc. 136, 11799–11804 (2014). 25061804 10.1021/ja5060302 PMC4140454

[b7] Hu L. H & Sim K. Y. Complex caged polyisoprenylated benzophenone derivatives, sampsoniones A and B, from *Hypericum sampsonii*. Tetrahedron Lett. 39, 7999–8002 (1998).

[b8] Hu L. H. & Sim K. Y. Cytotoxic polyprenylated benzoylphloroglucinol derivatives with an unusual adamantyl skeleton from *Hypericum sampsonii* (Guttiferae). Org. Lett. 1, 879–882 (1999). 10823216 10.1021/ol9907825

[b9] Hu L. H. & Sim K. Y. Sampsoniones C–H, a unique family of polyprenylated benzophenone derivatives with the novel tetracyclo[7.3.1.1^3,11^.0^3,7^]tetradecane-2,12,14-trione skeleton, from *Hypericum sampsonii* (Guttiferae). Tetrahedron Lett. 40, 759–762 (1999).

[b10] Hu L. H. & Sim K. Y. Sampsoniones A–M, a unique family of caged polyprenylated benzoylphloroglucinol derivatives, from *Hypericum sampsonii*. Tetrahedron 56, 1379–1386 (2000).

[b11] Xiao Z. Y., Mu Q., Shiu W. K. P., Zeng Y. H. & Gibbons S. Polyisoprenylated benzoylphloroglucinol derivatives from *Hypericum sampsonii*. J. Nat. Prod. 70, 1779–1782 (2007). 18038963 10.1021/np0704147

[b12] Zeng Y. H. *et al.* Geranyl bearing polyisoprenylated benzoylphloroglucinol derivatives from *Hypericum sampsonii*. Chem. Lett. 38, 440–441 (2009). 10.1021/np0704147 18038963

[b13] Xiao Z. Y., Zeng Y. H., Mu Q., Shiu W. K. P. & Gibbons S. Prenylated benzophenone peroxide derivatives from *Hypericum sampsonii*. Chem. Biodiversity 7, 953–958 (2010). 10.1002/cbdv.200900247 20397228

[b14] Zeng Y. H., Osman K., Xiao Z. Y., Gibbons S. & Mu Q. Four geranyl-bearing polyisoprenylated benzoylphloroglucinol derivatives from *Hypericum sampsonii*. Phytochemistry Lett. 5, 200–205 (2012).

[b15] Tian W. J. *et al.* Norsampsones A–D, four new decarbonyl polycyclic polyprenylated acylphloroglucinols from *Hypericum sampsonii*. Org. Lett. 16, 3448–3451 (2014). 24932990 10.1021/ol501333k

[b16] Dai Y. *et al.* Novel polycyclic polyprenylated acylphloroglucinols from *Hypericum sampsonii*. Tetrahedron 80, 822–822 (2014).

[b17] Lin Y. L. & Wu Y. S. Polyprenylated phloroglucinol derivatives from *Hypericum sampsonii*. Helv. Chim. Acta 86, 2156–2163 (2003).

[b18] Xin W. B. *et al.* Prenylated phloroglucinol derivatives from *Hypericum sampsonii*. Fitoterapia 83, 1540–1547 (2012). 22981504 10.1016/j.fitote.2012.08.022

[b19] Zhu H. *et al.* Bioactive acylphloroglucinols with adamantyl skeleton from *Hypericum sampsonii*. Org. Lett. 16, 6322–6325 (2014). 25453445 10.1021/ol5030579

[b20] Zhu H. *et al.* Hyperascyrones A–H, polyprenylated spirocyclic acylphloroglucinol derivatives from *Hypericum ascyron* Linn. Phytochemistry 115, 222–230 (2015). 25800107 10.1016/j.phytochem.2015.02.009

[b21] Li D. *et al.* Hyperattenins A–I, bioactive polyprenylated acylphloroglucinols from *Hypericum attenuatum* Choisy. RSC Adv. 5, 5277–5287 (2015).

[b22] Li D. *et al.* Two new adamantyl-like polyprenylated acylphloroglucinols from *Hypericum attenuatum* choisy. Tetrahedron Lett. 56, 1953–1955 (2015).

[b23] Chen C. *et al.* A new 3, 4-seco-oleanane-type triterpenoid with an unusual enedione moiety from *Hypericum ascyron*. Fitoterapia 103, 227–230 (2015). 25870936 10.1016/j.fitote.2015.04.009

[b24] Zhou Z. B., Zhang Y. M., Pan K., Luo J. G. & Kong L.Y. Cytotoxic polycyclic polyprenylated acylphloroglucinols from *Hypericum attenuatum*. Fitoterapia 95, 1–7 (2014). 24603092 10.1016/j.fitote.2014.02.011

[b25] Ishida Y. *et al.* Polyprenylated benzoylphloroglucinol-type derivatives including novel cage compounds from *Hypericum erectum*. Chem. Pharm. Bull. 58, 336–343 (2010). 20190438 10.1248/cpb.58.336

[b26] LaPlante S. R., Edwards P. J., Fader L. D., Jakalian A. & Hucke O. Revealing atropisomer axial chirality in drug discovery. Chem. Med. Chem. 6, 505–513 (2011). 21360821 10.1002/cmdc.201000485

[b27] Luo Q. *et al.* Applanatumin A, a new dimeric meroterpenoid from *Ganoderma applanatum* that displays potent antifibrotic activity. Org. Lett. 17, 1110–1113 (2015). 25706347 10.1021/ol503610b

[b28] Boukouvalas J., Pouliot R. & Fréchette Y. Concise synthesis of yingzhaosu C and *epi*-yingzhaosu C by peroxyi radical cyclization. Assignment of relative configuration. Tetrahedron Lett. 36, 4167–4170 (1995).

[b29] Elmore S. Apoptosis: A review of programmed cell death. Toxicol. Pathol. 35, 495–516 (2007). 17562483 10.1080/01926230701320337 PMC2117903

[b30] Henry G. E., Jacobs H., Carrington C. M. S., McLean S. & Reynolds W. F. Plukenetione A. An unusual adamantyl ketone from *Clusia plukenetii* (guttiferae). Tetrahedron Lett. 37, 8663–8666 (1996).

[b31] Zhang J. J. *et al.* Hyperuralones A and B, new acylphloroglucinol derivatives with intricately caged cores from *Hypericum uralum*. Org. Lett. 16, 4912–4915 (2014). 25192496 10.1021/ol502425f

[b32] Liao Y. *et al.* Hypersubones A and B, new polycyclic acylphloroglucinols with intriguing adamantane type cores from *Hypericum subsessile*. Org. Lett. 17, 1172–1175 (2015). 25699579 10.1021/acs.orglett.5b00100

[b33] Bruhn T. H., Schaumlöffel Y., Bringmann A., G. Spec Dis, version 1.51, University of Würzburg, Germany (2010).

[b34] Rychnovsky S. D. Predicting NMR spectra by computational methods: Structure revision of hexacyclinol. Org. Lett. 8, 2895–2898 (2006). 16774284 10.1021/ol0611346

