# Supplementary material for: Hyperisampsins H–M, Cytotoxic Polycyclic Polyprenylated Acylphloroglucinols from Hypericum sampsonii
Source: Sci Rep. 2015 Oct 6;5:14772. doi: 10.1038/srep14772 (PMC4594001; doi:10.1038/srep14772)

Hyperisampsins H−M, Cytotoxic Polycyclic Polyprenylated Acylphloroglucinols from Hypericum sampsonii

Hucheng Zhu[1], Chunmei Chen[1], Qingyi Tong[1], Xintao Chen[1], Jing Yang[2], Junjun Liu[1], Bin Sun[1], Jianping Wang[1], Guangmin Yao[1], Zengwei Luo[1], Yongbo Xue[1,]*, & Yonghui Zhang[1,]*

[1]Hubei Key Laboratory of Natural Medicinal Chemistry and Resource Evaluation, School of Pharmacy, Tongji Medical College, Huazhong University of Science and Technology, Wuhan 430030, China

[2]State Key Laboratory of Phytochemistry and Plant Resources in West China, Kunming Institute of Botany, Chinese Academy of Sciences, Kunming 650204, China

* **Corresponding Authors**

* Tel./fax: +86-027-83692892

Emails: zhangyh@mails.tjmu.edu.cn (Y.Z.);

yongboxue@mail.hust.edu.cn (Y.X.)

CONTENTS

Figure S1. TLC detection experiment for compounds **3** and **6** 1

ECD Calculation of Models A and B for compounds **1**–**6** 1

^{13}C NMR calculation of **4a** and **4b**..9

HRESIMS, UV, IR, and NMR spectra of **1** 12

HRESIMS, UV, IR, and NMR spectra of **2** 16

HRESIMS, UV, IR, and NMR spectra of **3** 21

HRESIMS, UV, IR, and NMR spectra of **4** 28

HRESIMS, UV, IR, and NMR spectra of **5** 32

HRESIMS, UV, IR, and NMR spectra of **6** 37

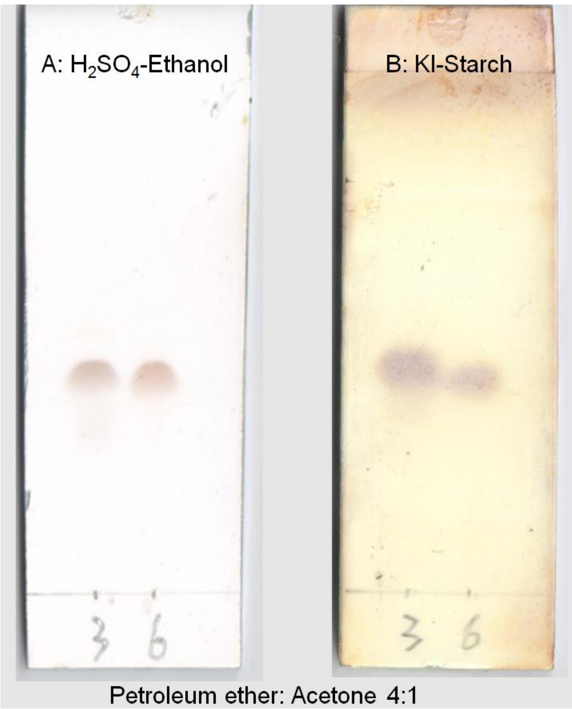

Figure S1. TLC detection experiment for compounds **3** and **6**.

ECD Calculation

ECD Calculation of Models A and B for compounds 1−6

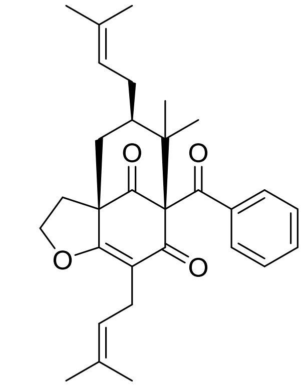

Model B

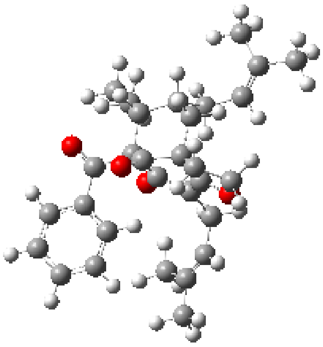

a

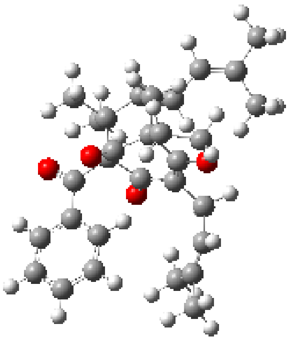

b

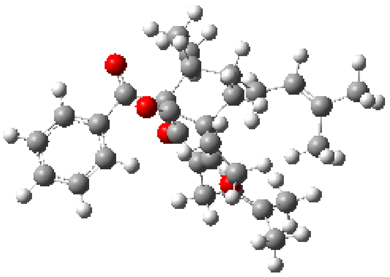

c

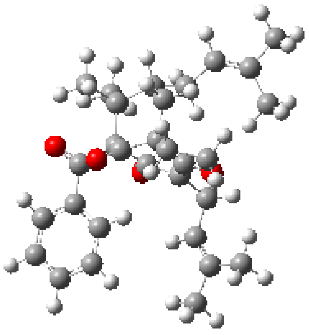

d

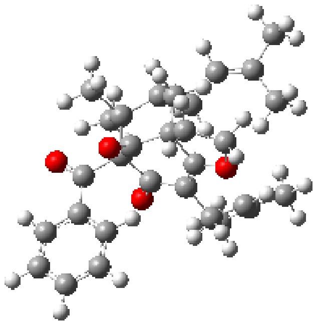

e

Optimized geometries of predominant conformers for compound **model B** at the B3LYP/6-31G(d,p) level in the gas phase.

Important thermodynamic parameters (a.u.) and Boltzmann distributions of the optimized compound **model B** at B3LYP/6-31G(d,p) level in the gas phase

Conformations	E+ZPE	G	%
**a**	-1465.176475	-1465.242471	9.6
**b**	-1465.178090	-1465.242912	8.9
**c**	-1465.177614	-1465.242392	15.3
**d**	-1465.178022	-1465.244214	60.9
**e**	-1465.177181	-1465.241914	5.3

E+ZPE, G: total energy with zero point energy (ZPE) and Gibbs free energy in the gas phase at B3LYP/6-31G(d,p) level., %: Boltzmann distributions, using the relative Gibbs free energies as weighting factors

Optimized Z-matrixes of **model B** in the gas phase (Å) at B3LYP/6-31G(d,p) level

**a**				**b**			
C	0.152029	-1.31908	-0.00302	C	0.298921	-1.42449	-0.16428
C	0.321922	-0.00084	-0.82224	C	0.274119	-0.0255	-0.857
C	-0.007	1.292864	-0.20965	C	-0.26997	1.136461	-0.14231
C	-0.5861	1.281971	1.013447	C	-0.88422	0.916451	1.044029
C	-0.96516	0.050266	1.802344	C	-1.09387	-0.4312	1.695795
C	0.067254	-1.02417	1.492674	C	0.113214	-1.28994	1.345621
C	0.328188	2.567632	-0.96107	C	-0.09686	2.51594	-0.75034
C	1.621454	3.205271	-0.49993	C	1.097703	3.261918	-0.19566
C	2.801799	3.217061	-1.13847	C	2.267478	3.50586	-0.80668
C	3.98892	3.934164	-0.54062	C	3.345159	4.300963	-0.10869
C	3.068366	2.553531	-2.46728	C	2.626526	3.042707	-2.19724

C	-1.24754	-2.04105	-0.3824	C	-0.94481	-2.32608	-0.67109
C	-2.35957	-0.52119	1.404004	C	-2.36476	-1.17592	1.182166
C	-2.4731	-1.11016	-0.02336	C	-2.32472	-1.65626	-0.28873
C	1.41459	-2.20059	-0.29795	C	1.699623	-2.05852	-0.47321
C	2.797824	-1.66396	-0.03988	C	2.966527	-1.34509	-0.08122
O	0.764159	-0.08714	-1.96409	O	0.753757	0.068944	-1.98286
C	3.085605	-0.41281	0.524041	C	3.025682	-0.11805	0.594533
C	4.405751	-0.01558	0.731649	C	4.254946	0.452661	0.92174
C	5.458282	-0.85993	0.377371	C	5.4434	-0.19223	0.57894
C	5.184404	-2.10809	-0.18718	C	5.397709	-1.41467	-0.09597
C	3.867534	-2.5049	-0.39186	C	4.171544	-1.98391	-0.42141
O	1.298093	-3.33221	-0.73468	O	1.78594	-3.14018	-1.02782
O	0.705725	-1.60187	2.348862	O	0.808204	-1.83437	2.179159
O	-0.86378	2.406941	1.706397	O	-1.35404	1.912888	1.823657
C	-1.33678	2.062958	3.038174	C	-1.82919	1.369153	3.086152
C	-0.94571	0.596	3.241541	C	-1.21616	-0.0309	3.177667
C	-2.9123	-0.0869	-1.12205	C	-2.92833	-0.61849	-1.28204
C	-4.05952	0.802087	-0.70735	C	-4.38429	-0.35956	-0.98833
C	-5.3713	0.599927	-0.91298	C	-5.01927	0.812767	-0.82703
C	-6.39507	1.606916	-0.44708	C	-6.50725	0.853412	-0.56921
C	-5.95031	-0.60452	-1.61367	C	-4.36352	2.16944	-0.90445
C	-1.40573	-3.33931	0.452801	C	-0.90878	-3.70329	0.045193
C	-1.29271	-2.43199	-1.87332	C	-0.87458	-2.58673	-2.18916
H	0.364655	2.326545	-2.02458	H	-0.01761	2.395639	-1.83187
H	-0.48777	3.285397	-0.81391	H	-0.99948	3.104843	-0.54787
H	1.559258	3.705615	0.466373	H	0.965052	3.634098	0.820212
H	4.82354	3.240602	-0.37292	H	4.261956	3.706749	0.000827
H	4.364527	4.708502	-1.22279	H	3.620697	5.187894	-0.69467
H	3.743972	4.413224	0.4116	H	3.032191	4.636239	0.884282
H	3.975439	1.939819	-2.40441	H	1.915091	2.326153	-2.60824
H	2.257454	1.900386	-2.79058	H	2.709512	3.896776	-2.88303
H	3.254052	3.304092	-3.24754	H	3.609441	2.555739	-2.18748
H	-2.57891	-1.30885	2.132902	H	-2.4906	-2.03922	1.844604
H	-3.12373	0.248448	1.549925	H	-3.24326	-0.53877	1.330571
H	-3.32521	-1.79989	0.034785	H	-3.03196	-2.49603	-0.33047
H	2.2945	0.270737	0.804505	H	2.124492	0.413655	0.87126
H	4.608422	0.955803	1.172132	H	4.279924	1.402427	1.446958
H	6.485898	-0.54755	0.54014	H	6.399653	0.254354	0.836278
H	5.998155	-2.77067	-0.46687	H	6.318383	-1.92277	-0.3673
H	3.636705	-3.47067	-0.82626	H	4.117378	-2.93232	-0.94319
H	-2.42039	2.220493	3.066557	H	-2.92415	1.351205	3.059024
H	-0.86132	2.754947	3.736108	H	-1.5057	2.054082	3.87239
H	0.061277	0.503533	3.657451	H	-0.22367	-0.00426	3.63581
H	-1.63434	0.067607	3.904396	H	-1.83533	-0.72201	3.75359
H	-3.1932	-0.66191	-2.00678	H	-2.85809	-1.03779	-2.29175
H	-2.08364	0.550148	-1.43337	H	-2.36247	0.311323	-1.2982
H	-3.77814	1.719994	-0.19004	H	-4.99431	-1.26306	-0.91893

H	-5.9311	2.459206	0.056953	H	-6.9416	-0.14858	-0.51506
H	-6.98237	1.99062	-1.29181	H	-6.73272	1.374417	0.370859
H	-7.11402	1.149834	0.245834	H	-7.02909	1.407228	-1.36081
H	-6.63537	-1.14595	-0.94828	H	-4.80492	2.762901	-1.71572
H	-6.54467	-0.29748	-2.48399	H	-4.53368	2.736925	0.019791
H	-5.19162	-1.31024	-1.95677	H	-3.28759	2.121	-1.07596
H	-0.6	-4.03893	0.239318	H	0.0192	-4.22986	-0.16863
H	-1.41857	-3.15924	1.531201	H	-1.01064	-3.62644	1.131213
H	-2.35468	-3.81657	0.186164	H	-1.74104	-4.31296	-0.32238
H	-0.55446	-3.20345	-2.0858	H	-0.03063	-3.23393	-2.42146
H	-2.28509	-2.8356	-2.10598	H	-1.79281	-3.0941	-2.50728
H	-1.09898	-1.58789	-2.53576	H	-0.76428	-1.67116	-2.77116
**c**				**d**			
C	1.345718	-0.96715	-0.33178	C	0.405505	-1.53689	-0.05594
C	0.72012	0.399642	-0.74775	C	0.312332	-0.26944	-0.96207
C	-0.12101	1.138811	0.198589	C	-0.23212	0.981828	-0.42216
C	-0.5026	0.503755	1.332428	C	-0.79593	0.947958	0.807802
C	-0.12178	-0.90856	1.724866	C	-0.95211	-0.28609	1.6698
C	1.27963	-1.15886	1.181307	C	0.265581	-1.16497	1.418467
C	-0.49959	2.568371	-0.13617	C	-0.12661	2.24025	-1.26212
C	-1.60277	2.679673	-1.16308	C	1.268718	2.823605	-1.27352
C	-2.79944	3.272429	-1.02736	C	1.683203	3.967699	-0.7082
C	-3.7596	3.334594	-2.19166	C	3.119522	4.414878	-0.84597
C	-3.30142	3.934849	0.232673	C	0.807429	4.908632	0.083274
C	0.497358	-2.19131	-0.95513	C	-0.82578	-2.53935	-0.36201
C	-1.05271	-2.01068	1.129983	C	-2.22106	-1.12327	1.319868
C	-0.98557	-2.19685	-0.4044	C	-2.21028	-1.84255	-0.05197
C	2.840821	-0.9289	-0.80275	C	1.814363	-2.17478	-0.3128
C	3.751361	0.187555	-0.36141	C	3.067697	-1.36086	-0.12752
C	3.423897	1.171175	0.583197	C	4.274834	-1.98641	-0.48377
C	4.34526	2.158251	0.928968	C	5.489918	-1.325	-0.34308
C	5.60769	2.179834	0.335763	C	5.522635	-0.02267	0.161255
C	5.946317	1.20533	-0.60586	C	4.332063	0.609328	0.520204
C	5.027676	0.218961	-0.9483	C	3.113868	-0.05347	0.377255
C	-1.32599	0.220271	3.457024	C	-1.68209	1.697492	2.789313
C	-0.2285	-0.82831	3.259778	C	-1.03633	0.338991	3.074728
C	-2.06181	-1.33893	-1.13848	C	-2.86538	-0.99671	-1.18528
C	-3.44006	-1.90178	-0.89692	C	-4.31699	-0.7153	-0.89274
C	-4.50346	-1.33301	-0.30646	C	-4.96022	0.463045	-0.85671
C	-5.8062	-2.08778	-0.17973	C	-6.4427	0.523117	-0.57237
C	-4.53752	0.064597	0.26087	C	-4.31955	1.806184	-1.10701
C	1.132987	-3.53787	-0.51537	C	-0.7315	-3.77578	0.57189
C	0.500629	-2.15447	-2.49629	C	-0.7928	-3.04713	-1.81736
O	1.004597	0.858091	-1.85003	O	0.746053	-0.33984	-2.10774
O	3.288738	-1.79272	-1.53571	O	1.91875	-3.3365	-0.66552
O	2.211213	-1.51185	1.875579	O	1.003541	-1.55117	2.302094
O	-1.23041	1.097908	2.301784	O	-1.26809	2.043739	1.439215

H	-0.75772	3.091372	0.786697	H	-0.39456	1.97246	-2.29154
H	0.396376	3.049778	-0.54903	H	-0.8585	2.968882	-0.90902
H	-1.35225	2.241648	-2.12741	H	1.999636	2.222524	-1.811
H	-3.35328	2.847425	-3.08183	H	3.716009	3.701118	-1.42011
H	-4.71506	2.85329	-1.94407	H	3.182182	5.390688	-1.34627
H	-3.99559	4.375298	-2.45135	H	3.590814	4.543581	0.137936
H	-3.42792	5.015013	0.080334	H	0.694136	5.868916	-0.4376
H	-4.29086	3.543396	0.501611	H	-0.1876	4.508288	0.283135
H	-2.64382	3.788965	1.091253	H	1.272478	5.137599	1.050935
H	-0.76173	-2.94205	1.627874	H	-2.31757	-1.86613	2.118998
H	-2.08905	-1.814	1.425437	H	-3.10682	-0.48201	1.384197
H	-1.30796	-3.23133	-0.58231	H	-2.89842	-2.69039	0.069043
H	2.453524	1.180933	1.063134	H	4.231751	-2.99799	-0.87055
H	4.07516	2.910417	1.664137	H	6.41222	-1.82354	-0.62637
H	6.323341	2.951164	0.605502	H	6.470363	0.496332	0.272883
H	6.926744	1.215837	-1.07282	H	4.347112	1.621598	0.912304
H	5.274999	-0.54624	-1.67492	H	2.210062	0.469035	0.663039
H	-2.33109	-0.21496	3.470735	H	-2.77648	1.658906	2.81088
H	-1.19802	0.842545	4.345019	H	-1.34342	2.50211	3.444971
H	0.724763	-0.49497	3.67911	H	-0.03027	0.452148	3.487653
H	-0.4802	-1.78811	3.715912	H	-1.62172	-0.26393	3.772234
H	-1.86901	-1.37598	-2.21555	H	-2.81763	-1.57976	-2.11143
H	-2.0095	-0.28876	-0.85445	H	-2.3191	-0.07508	-1.37806
H	-3.57006	-2.92328	-1.26116	H	-4.91493	-1.60903	-0.70072
H	-5.73864	-3.09627	-0.59663	H	-6.8663	-0.46867	-0.39224
H	-6.11427	-2.17293	0.870983	H	-6.65297	1.149291	0.304937
H	-6.61865	-1.56032	-0.69696	H	-6.98638	0.976877	-1.41157
H	-3.59587	0.604931	0.156768	H	-4.48298	2.478609	-0.25474
H	-4.80454	0.041709	1.326184	H	-4.77862	2.294429	-1.97659
H	-5.31787	0.657784	-0.23421	H	-3.2454	1.748098	-1.28656
H	2.166076	-3.60848	-0.84969	H	0.205282	-4.30818	0.41932
H	1.116832	-3.68711	0.567791	H	-0.80604	-3.51979	1.632298
H	0.568544	-4.35859	-0.97046	H	-1.55583	-4.45738	0.337051
H	1.495112	-2.38296	-2.8769	H	0.06264	-3.70327	-1.9692
H	-0.19348	-2.91428	-2.87387	H	-1.70475	-3.62256	-2.01496
H	0.205253	-1.1843	-2.89723	H	-0.72772	-2.2377	-2.54525
**e**							
C	1.206796	-0.88846	-0.5279				
C	0.668665	0.576122	-0.5065				
C	-0.08239	1.06102	0.656969				
C	-0.46497	0.147572	1.581329				
C	-0.18632	-1.33953	1.531677				
C	1.175394	-1.50516	0.86884				
C	-0.36796	2.547244	0.781363				
C	-1.79992	2.945311	0.494328				
C	-2.27895	3.526433	-0.61707				
C	-3.73622	3.909549	-0.7167				

C	-1.45049	3.862436	-1.83332				
C	0.253938	-1.82964	-1.42855				
C	-1.21907	-2.15722	0.694813				
C	-1.20849	-1.89979	-0.83107				
C	2.685485	-0.80812	-1.04489				
C	3.689103	0.078728	-0.35516				
C	4.944471	0.197868	-0.9752				
C	5.944121	0.987106	-0.41681				
C	5.709321	1.670635	0.778518				
C	4.468853	1.558006	1.406634				
C	3.466124	0.769601	0.844729				
C	-1.23454	-0.67967	3.579935				
C	-0.23564	-1.69995	3.029031				
C	-2.24135	-0.80315	-1.237				
C	-3.64556	-1.34885	-1.15399				
C	-4.66987	-0.96143	-0.37703				
C	-6.00962	-1.65356	-0.47055				
C	-4.62109	0.167159	0.622759				
C	0.801642	-3.2828	-1.41674				
C	0.214574	-1.35676	-2.89519				
O	0.957722	1.306637	-1.44993				
O	3.045547	-1.45476	-2.01254				
O	2.102142	-2.0959	1.385018				
O	-1.1036	0.48675	2.720882				
H	-0.12915	2.852573	1.80813				
H	0.322544	3.068469	0.116737				
H	-2.49859	2.746281	1.306318				
H	-3.8454	4.984878	-0.91117				
H	-4.22345	3.393144	-1.55414				
H	-4.28731	3.67386	0.197947				
H	-1.96689	3.532048	-2.74334				
H	-0.46635	3.392768	-1.82517				
H	-1.32416	4.949469	-1.92947				
H	-0.99264	-3.21017	0.893483				
H	-2.22797	-1.98105	1.083453				
H	-1.60957	-2.81841	-1.2792				
H	5.110755	-0.34228	-1.90001				
H	6.9068	1.070241	-0.91253				
H	6.488304	2.287297	1.217588				
H	4.279234	2.084275	2.33737				
H	2.514982	0.702517	1.357407				
H	-2.27153	-1.02773	3.52325				
H	-1.02621	-0.35193	4.600288				
H	0.755514	-1.56988	3.472076				
H	-0.55042	-2.73079	3.204894				
H	-2.05822	-0.50906	-2.2754				
H	-2.13217	0.102216	-0.64114				

H	-3.83412	-2.1845	-1.8316				
H	-6.79964	-0.94544	-0.75398				
H	-6.00196	-2.4635	-1.20488				
H	-6.307	-2.07456	0.499222				
H	-4.90469	-0.19011	1.622038				
H	-5.34953	0.945396	0.359568				
H	-3.64239	0.642302	0.695334				
H	1.819632	-3.31904	-1.79908				
H	0.798643	-3.73535	-0.42128				
H	0.170427	-3.90184	-2.06298				
H	1.177987	-1.52759	-3.37329				
H	-0.54397	-1.93404	-3.43654				
H	-0.02225	-0.29654	-2.99092				

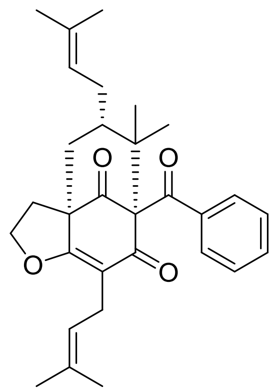

Model A

Key transitions, oscillator strengths, and rotatory strengths in the ECD of conformer **d** at B3LYP-PCM/6-31G(d,p)//B3LYP/6-31G(d,p) level.

Species	Excited State	$\Delta E(eV)^a$	$\lambda(nm)^b$	f^c	$R_{vel}{}^d$
**d**	121 ->125	3.6994	335.15	0.0006	-10.1619
	120 ->126	3.9366	314.96	0.0014	-5.3587
	124 ->125	3.9767	311.78	0.0035	11.7687
	124 ->126	4.0882	303.27	0.0085	0.0162
	123 ->125	4.1509	298.7	0.0112	31.3510
	122 ->125	4.1608	297.98	0.0059	-2.9578
	120 ->127	4.255	291.38	0.0006	3.1372
	123 ->126	4.2617	290.93	0.0016	-3.6199
	120 ->125	4.4252	280.18	0.0061	-9.2609
	124 ->127	4.5122	274.77	0.0256	-14.0792
	122 ->126	4.5694	271.33	0.0795	-29.3259
	119 ->125	4.5739	271.07	0.0140	-28.2205
	123 ->127	4.7245	262.43	0.0296	-25.6998
	122 ->127	4.7501	261.01	0.0145	-18.8788
	122 ->127	4.8854	253.78	0.0632	76.4455

	118 ->125	4.9092	252.55	0.1488	-35.7894
	117 ->125	4.9567	250.14	0.0176	-39.1012
	119 ->126	4.9934	248.3	0.0097	4.6845
	121 ->127	5.0146	247.24	0.0687	-18.8414
	118 ->126	5.0568	245.18	0.0933	87.8902
	118 ->127	5.3401	232.17	0.0122	-8.4760
	119 ->127	5.3516	231.68	0.0020	2.1271
	117 ->126	5.3984	229.67	0.0199	-25.9849
	124 ->128	5.4171	228.87	0.0027	0.0429
	117 ->127	5.5616	222.93	0.0348	46.3549
	122 ->128	5.7053	217.31	0.0037	0.6905
	123 ->128	5.7365	216.13	0.0002	-0.0104
	121 ->128	5.9319	209.01	0.0029	2.7933
	116 ->125	6.0452	205.09	0.0011	-0.9172
	120 ->128	6.0831	203.82	0.0149	-3.6431
	115 ->125	6.2075	199.73	0.0022	-2.5003
	114 ->125	6.237	198.79	0.0240	68.5400
	118 ->128	6.2416	198.64	0.0458	-16.5442
	116 ->126	6.2796	197.44	0.0075	-8.5730
	115 ->126	6.318	196.24	0.0606	-57.3246
	124 ->129	6.3227	196.09	0.1025	-13.5618
	114 ->126	6.4219	193.06	0.0026	-1.9407
	124 ->131	6.4435	192.42	0.0030	8.1950
	110 ->125	6.5049	190.6	0.0027	3.4526
	124 ->130	6.5106	190.43	0.0022	4.7830
	123 ->129	6.5559	189.12	0.0012	-2.6128
	119 ->128	6.5994	187.87	0.1695	-72.3886
	113 ->125	6.6123	187.51	0.0319	-17.7043
	115 ->127	6.6304	186.99	0.0046	-2.6670
	122 ->129	6.6577	186.23	0.1394	82.3816
	116 ->127	6.6719	185.83	0.0348	18.8666
	116 ->127	6.6938	185.22	0.0540	-4.7142
	113 ->126	6.7177	184.56	0.0005	-1.0486
	117 ->128	6.7267	184.32	0.0551	-10.5526
	113 ->126	6.7282	184.28	0.0293	1.3335

[a]Excitation energy. [b]Wavelength. [c]Oscillator strength. [d]Rotatory strength in velocity form (10^{-40} cgs.).

^{13}C NMR calculation of 4a and 4b

		4a			4b		
NO.	Exptl.	Calcd.	Corrected	*Δδ*	Calcd.	Corrected	*Δδ*
1	77.2	70.3728	78.68328	1.48328	70.3527	78.0264549	0.8264549
2	193.9	180.3252	192.758895	-1.141105	180.7284	192.894446	-1.005554
3	115.8	104.5771	114.170241	-1.629759	104.2308	113.283394	-2.516606
4	172.2	162.3149	174.073209	1.8732088	162.9309	174.372588	2.1725876
5	58.2	53.2381	60.9060288	2.7060288	53.2601	60.2381861	2.0381861
6	36.5	29.4656	36.24206	-0.25794	29.8173	35.8412641	-0.658736
7	47.7	40.6754	47.8722275	0.1722275	40.3815	46.8354271	-0.864573
8	49.3	46.8228	54.250155	4.950155	46.8319	53.5483583	4.2483583
9	205.8	196.4952	209.53527	3.73527	196.8956	209.719651	3.9196509
10	193.4	182.2966	194.804223	1.4042225	182.7147	194.961588	1.5615883
11	136.9	121.8616	132.10291	-4.79709	122.1413	131.922851	-4.977149
12	128.1	116.4346	126.472398	-1.627602	116.0898	125.625055	-2.474945
13	127.9	113.3234	123.244528	-4.655472	113.3754	122.800179	-5.099821
14	132	119.2549	129.398459	-2.601541	119.3902	129.059781	-2.940219
15	127.9	114.5829	124.551259	-3.348741	114.9978	124.48861	-3.41139
16	128.1	117.4027	127.476801	-0.623199	117.5557	127.150617	-0.949383
17	22.4	17.8925	24.2349688	1.8349688	17.9233	23.4631783	1.0631783
18	119.5	109.0677	118.829239	-0.670761	110.552	119.861866	0.3618664
19	132.6	123.364	133.66165	1.06165	120.4741	130.187796	-2.412204
20	25.8	18.8899	25.2697713	-0.530229	18.1823	23.7327196	-2.06728
21	17.8	10.2703	16.3269363	-1.473064	10.6903	15.9357952	-1.864205
22	30.9	26.3778	33.0384675	2.1384675	27.5343	33.465346	2.565346
23	87.1	79.3875	88.0360313	0.9360313	80.7328	88.829025	1.729025
24	80.9	75.3988	83.897755	2.997755	78.5535	86.5610275	5.6610274
25	27.5	17.712	24.0477	-3.4523	22.0883	27.7976938	0.2976938
26	17.4	11.8482	17.9640075	0.5640075	12.7529	18.082343	0.682343
27	19.4	14.6274	20.8474275	1.4474275	17.3616	22.8786171	3.4786171
28	84.4	77.3077	85.8782388	1.4782387	84.0569	92.2884158	7.8884158
29	83.1	76.7162	85.2645575	2.1645575	76.6605	84.5909824	1.4909824
30	20.6	10.5263	16.5925363	-4.007464	11.8188	17.1102252	-3.489775
31	21.1	17.0544	23.36544	2.26544	18.0849	23.6313554	2.5313554
32	28.9	23.0932	29.630695	0.730695	22.945	28.6892615	-0.210738
33	124.3	114.5038	124.469193	0.1691925	114.5474	124.019879	-0.280121
34	133	122.9734	133.256403	0.2564025	122.9166	132.729706	-0.270294
35	25.8	18.8022	25.1787825	-0.621217	18.747	24.3204029	-1.479597
36	17.7	10.5474	16.6144275	-1.085573	10.4894	15.7267186	-1.973281
37	22.2	15.329	21.5753375	-0.624662	15.1698	20.5976109	-1.602389
38	26.9	19.3431	25.7399663	-1.160034	19.2585	24.852721	-2.047279
			**average: 1.8072889**			**average: 2.239795**	

^{13}C NMR calculation of 5a and 5b

		5a			5b		
NO.	Exptl.	Calcd.	Corrected	*Δδ*	Calcd.	Corrected	*Δδ*
1	77.7	70.0886	78.31117	0.611173	70.3403	77.96494	0.264939
2	193.8	179.9829	192.5133	-1.28667	180.4223	192.8795	-0.92046
3	115.8	104.446	114.0154	-1.78462	103.9244	113.0234	-2.77662
4	172.6	161.7319	173.5469	0.94689	161.7395	173.3766	0.776564
5	58.2	53.5342	61.10784	2.907841	53.3552	60.23419	2.034193
6	36.7	30.3198	36.98344	0.283436	29.8832	35.73177	-0.96823
7	47.7	40.7897	47.86376	0.163756	40.6487	46.96988	-0.73012
8	49.2	46.5671	53.86763	4.66763	47.0715	53.67464	4.474639
9	205.4	196.8147	210.0049	4.604936	196.8891	210.0692	4.669231
10	193.4	182.4652	195.0929	1.692936	182.6703	195.2262	1.826226
11	136.9	121.7028	131.9486	-4.95135	121.8749	131.7619	-5.13809
12	128.1	117.1408	127.2078	-0.89218	117.05	126.7252	-1.3748
13	127.9	113.7404	123.6741	-4.22588	113.761	123.2918	-4.60819
14	132	119.2806	129.4315	-2.5685	119.0901	128.8549	-3.14514
15	127.9	114.3289	124.2857	-3.61431	114.2892	123.8432	-4.0568
16	128.1	117.1687	127.2368	-0.86319	117.1941	126.8756	-1.22438
17	22.3	17.742	23.91259	1.612586	18.8883	24.2542	1.954196
18	119.4	109.5852	119.356	-0.04396	110.1285	119.4998	0.099841
19	132.9	122.3031	132.5725	-0.32752	122.3513	132.2592	-0.64078
20	25.8	18.8391	25.05269	-0.74731	18.5738	23.92589	-1.87411
21	17.8	10.0729	15.94286	-1.85714	10.2714	15.25901	-2.54099
22	31	24.7142	31.1581	0.158097	25.649	31.31169	0.311691
23	86.8	80.2738	88.89563	2.095633	80.8972	88.98529	2.185287
24	81.5	72.7605	81.08781	-0.41219	76.7618	84.66834	3.168343
25	27.5	23.2461	29.63245	2.132447	23.2328	28.78942	1.28942
26	15.4	11.1244	17.03558	1.635576	13.726	18.86527	3.465271
27	19.4	16.5233	22.64611	3.246113	17.0018	22.28488	2.884879
28	84.3	77.8511	86.37796	2.077963	83.5167	91.71978	7.419783
29	83	78.9517	87.52171	4.521707	77.1858	85.11096	2.110957
30	20.6	9.6597	15.51346	-5.08654	11.4694	16.50961	-4.09039
31	21.1	14.3472	20.38471	-0.71529	19.3581	24.74462	3.644621
32	28.9	23.0258	29.40351	0.503511	23.184	28.73848	-0.16152
33	124.3	114.8834	124.8619	0.561929	114.9408	124.5234	0.223401
34	133	122.3515	132.6228	-0.37722	122.4001	132.3102	-0.68984
35	25.8	18.7287	24.93797	-0.86203	18.7752	24.13613	-1.66387
36	17.8	10.5472	16.43575	-1.36425	10.6151	15.6178	-2.1822
37	22.3	15.2629	21.33631	-0.96369	15.2495	20.45565	-1.84435
38	26.9	19.2236	25.45227	-1.44773	19.267	24.64952	-2.25048
			**average: 1.8109403**			**average: 2.2548644**	

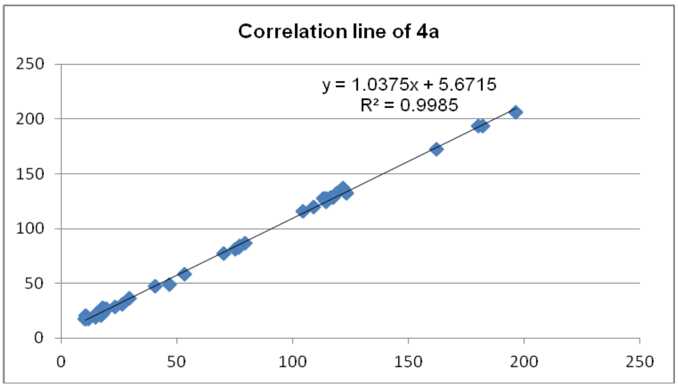
Correlation line of 4a
y = 1.0375x + 5.6715
R² = 0.9985
0
50
100
150
200
250

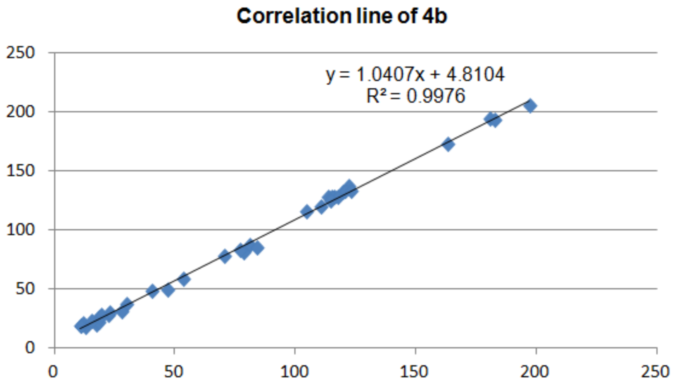
Correlation line of 4b
y = 1.0407x + 4.8104
R² = 0.9976
0
50
100
150
200
250

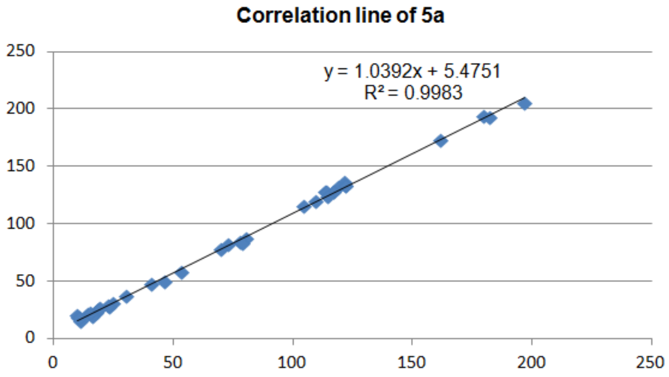
Correlation line of 5a
y = 1.0392x + 5.4751
R² = 0.9983
0
50
100
150
200
250

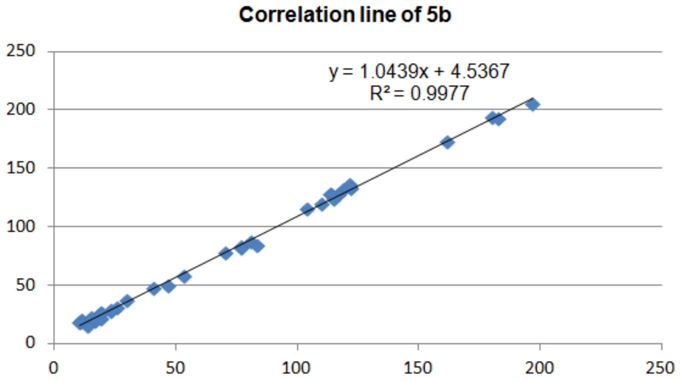
Correlation line of 5b
y = 1.0439x + 4.5367
R² = 0.9977
0
50
100
150
200
250

HRESIMS of compound 1

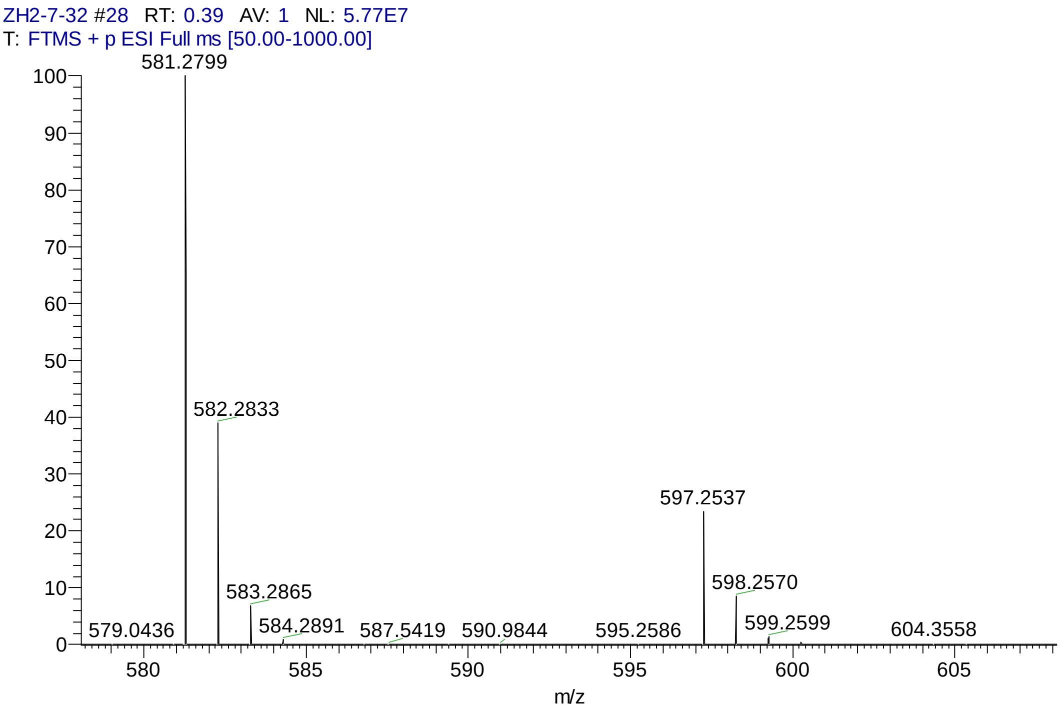

UV spectrum of compound 1

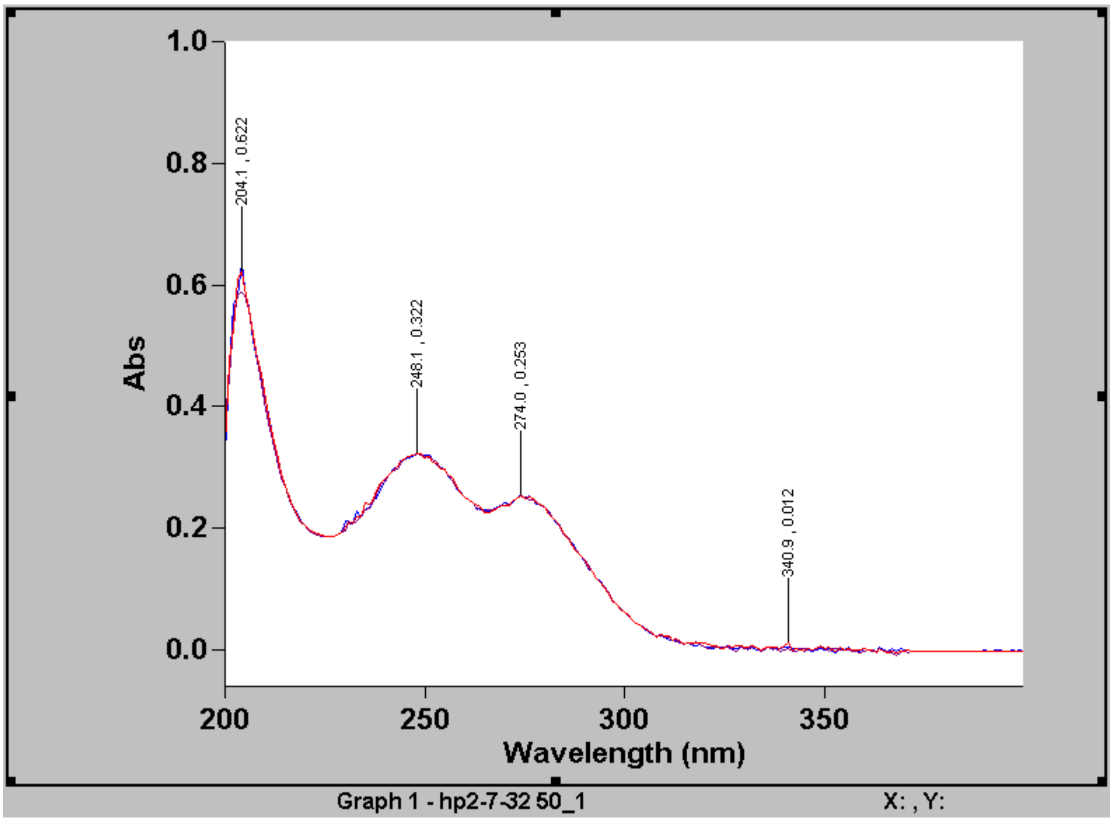

IR spectrum of compound 1

E:\同济医学院\张勇慧\20130314\zh2-7-32.0

仪器型号：Bruker Vertex 70

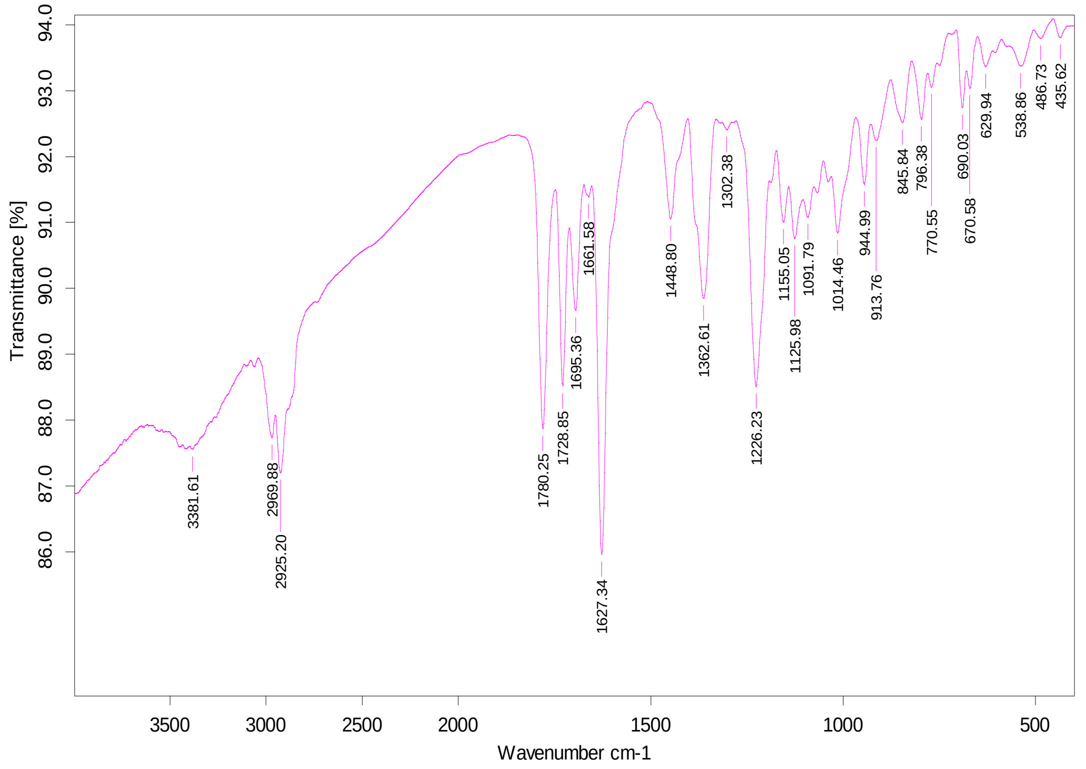

^{1}H NMR of compound 1

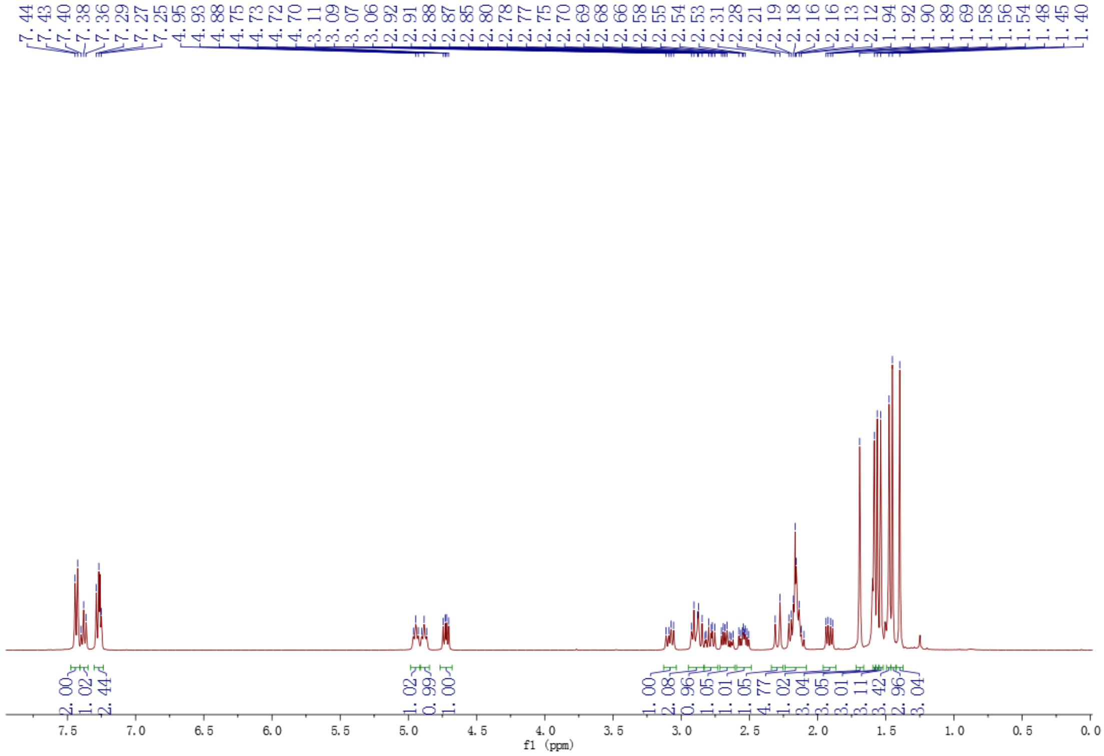

^{13}C NMR and DEPT of compound 1

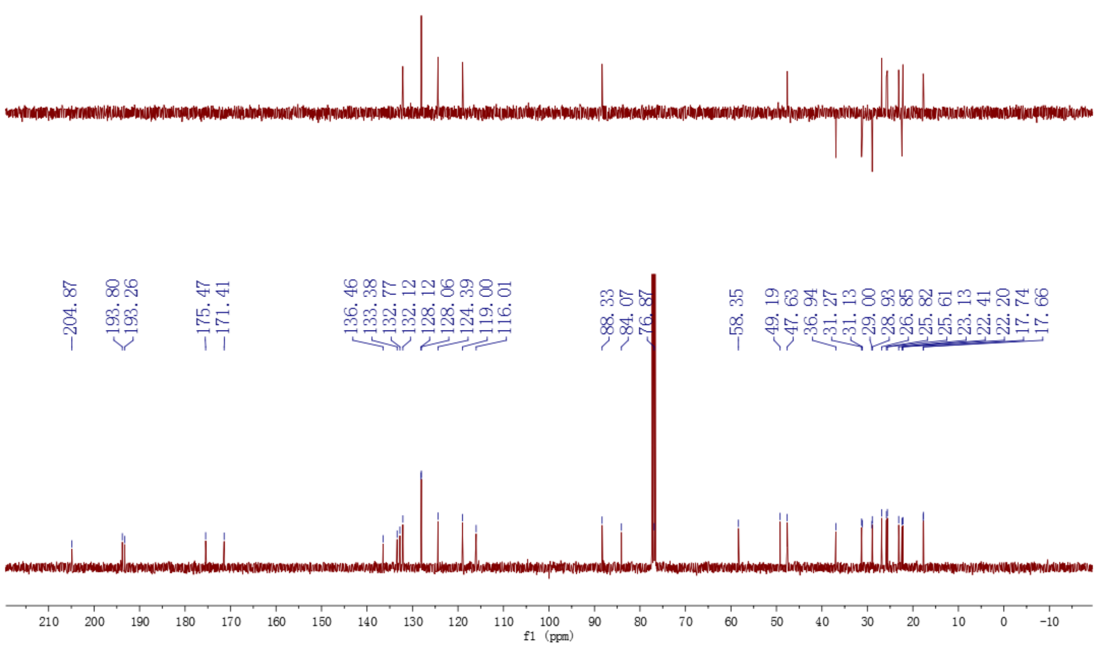

HSQC of compound 1

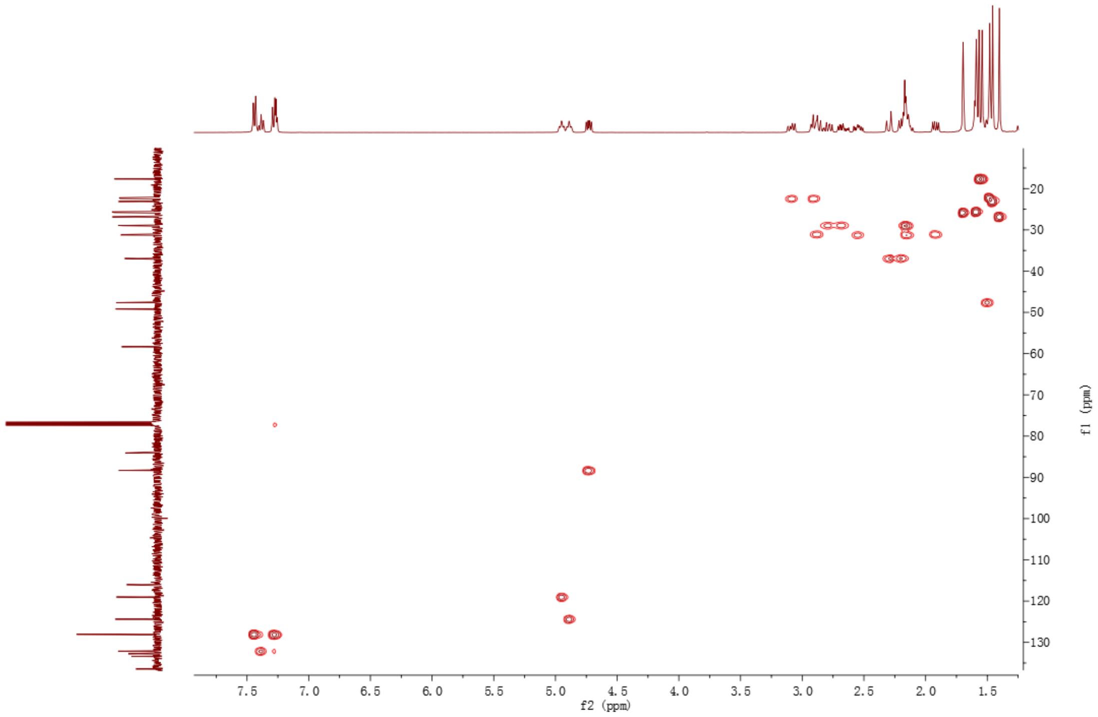

HMBC of compound 1

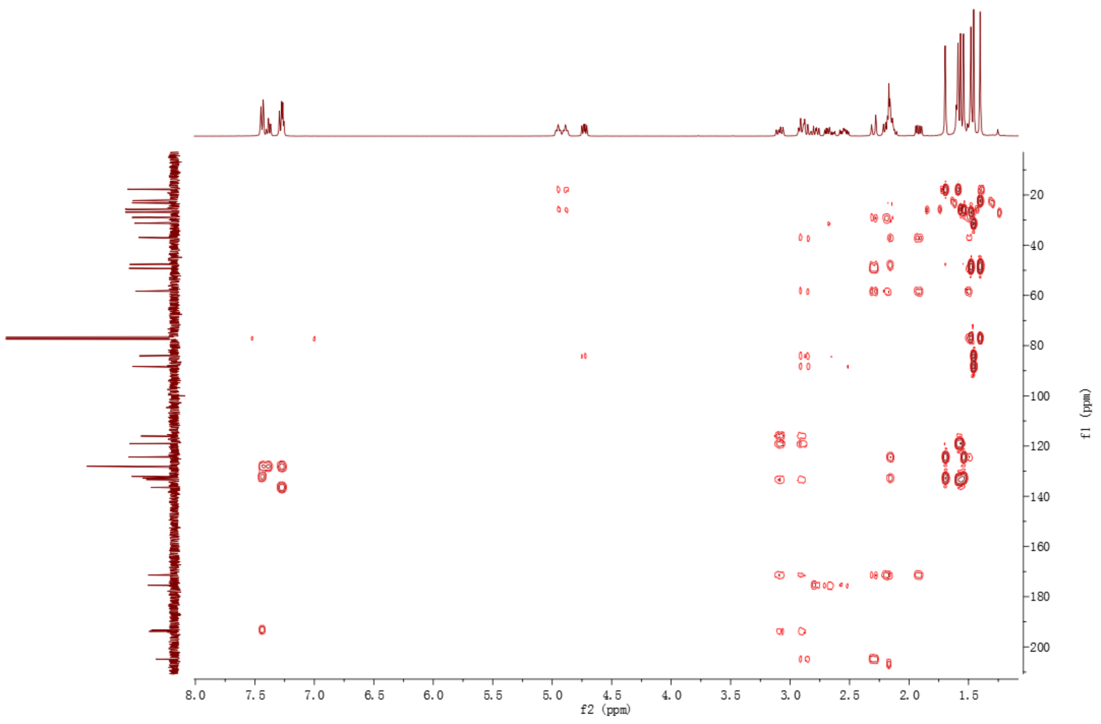

^{1}H–^{1}H COSY of compound 1

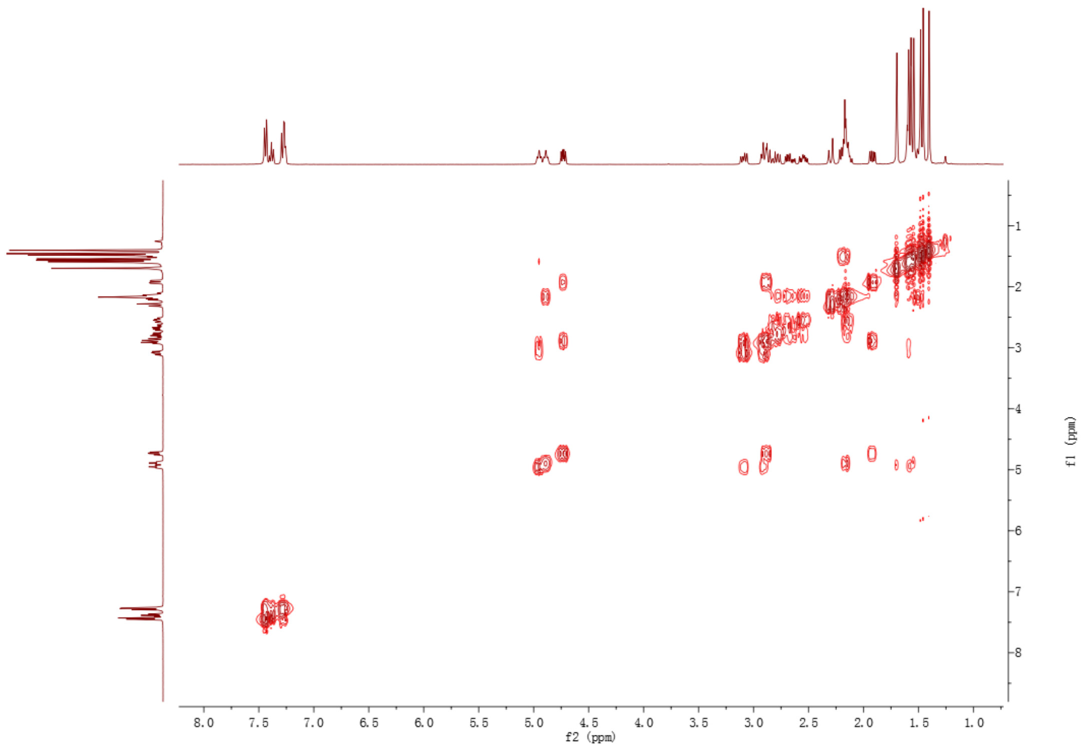

NOESY of compound 1

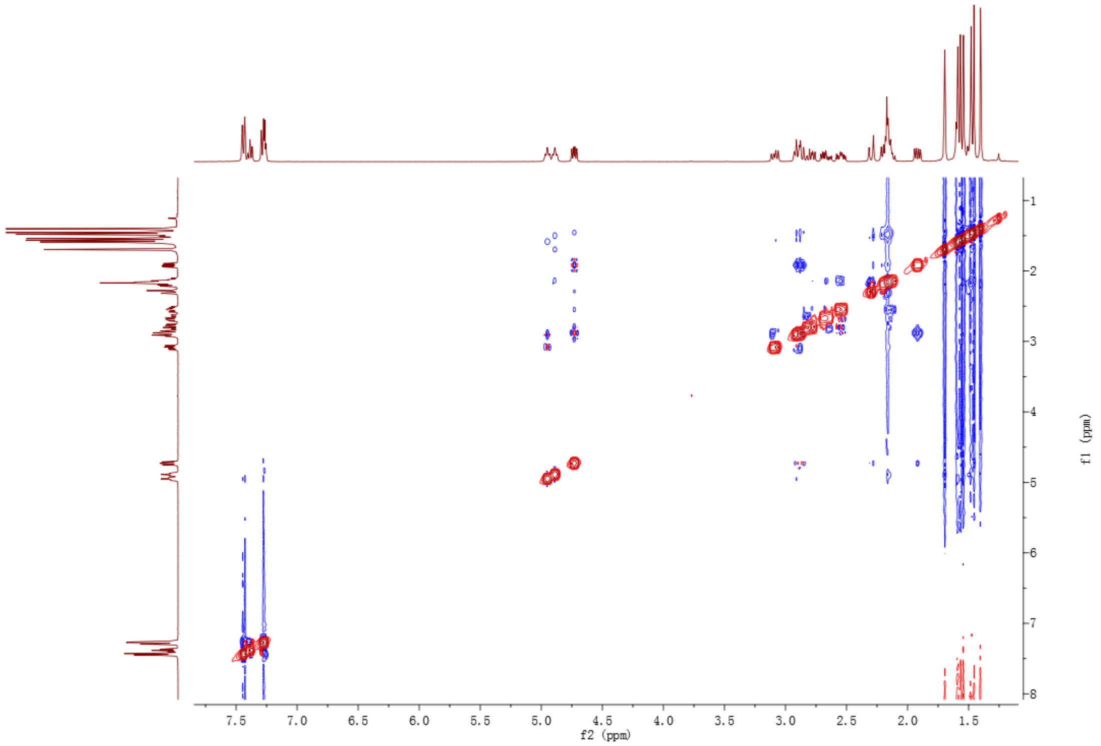

HRESIMS of compound 2

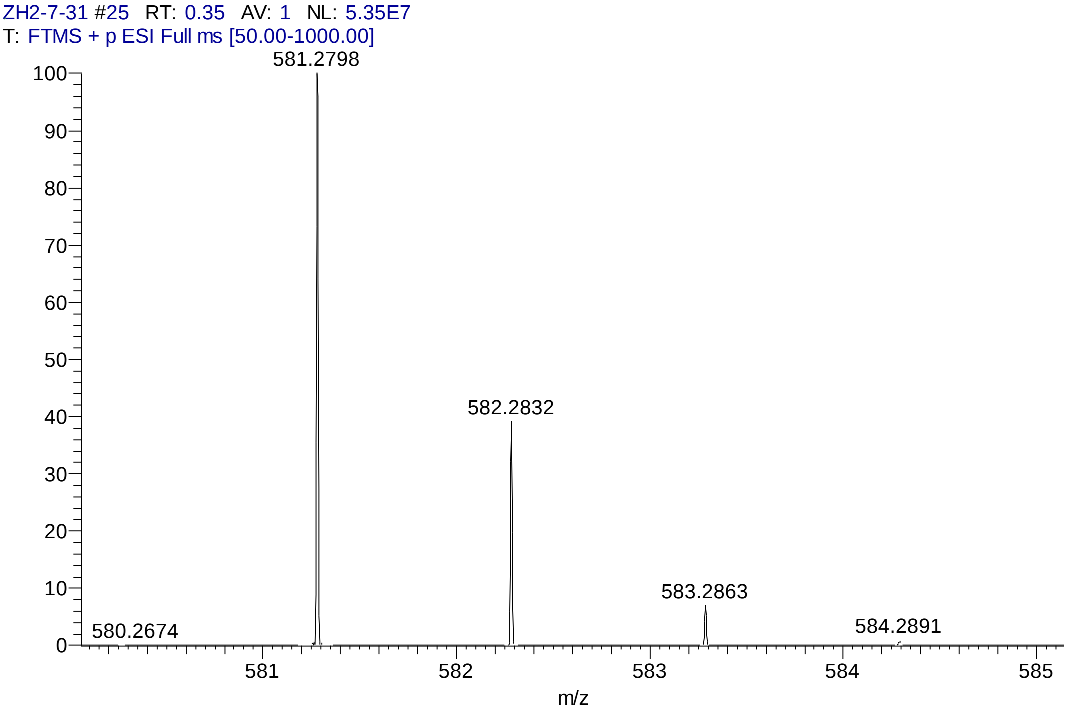

UV spectrum of compound 2

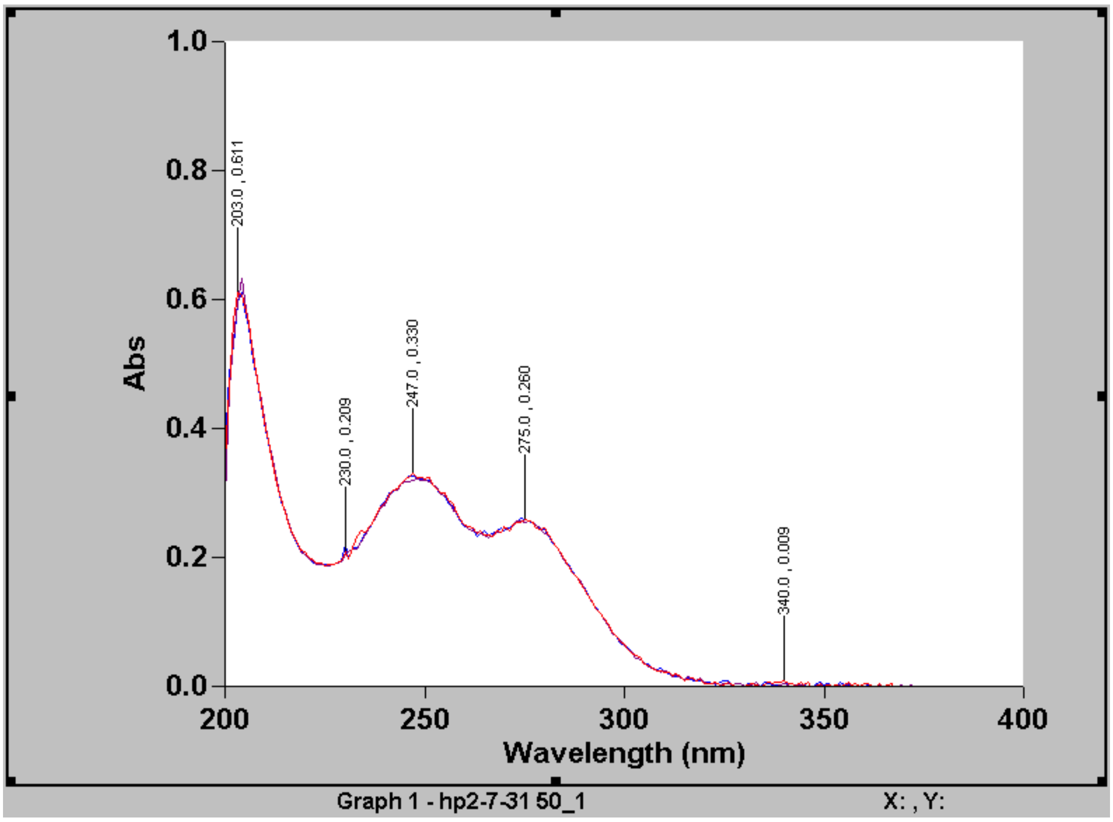

IR spectrum of compound 2

E:\同济医学院\张勇慧\20130314\zh2-7-31.2

仪器型号：Bruker Vertex 70

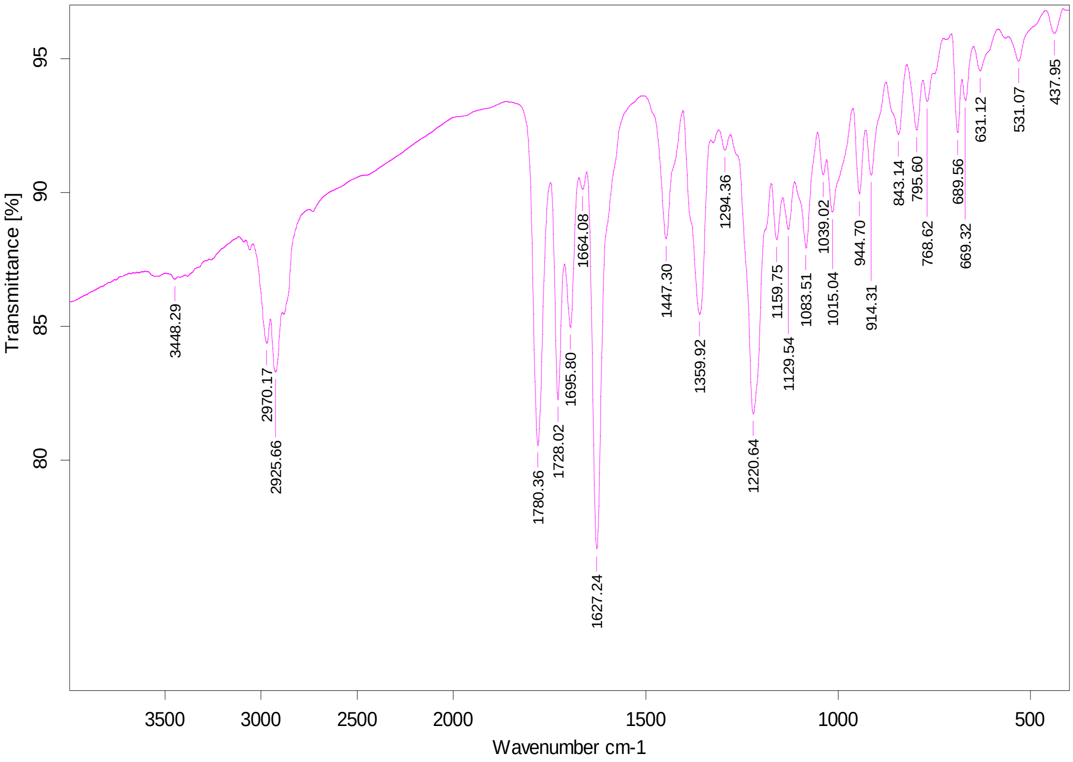

^{1}H NMR of compound 2

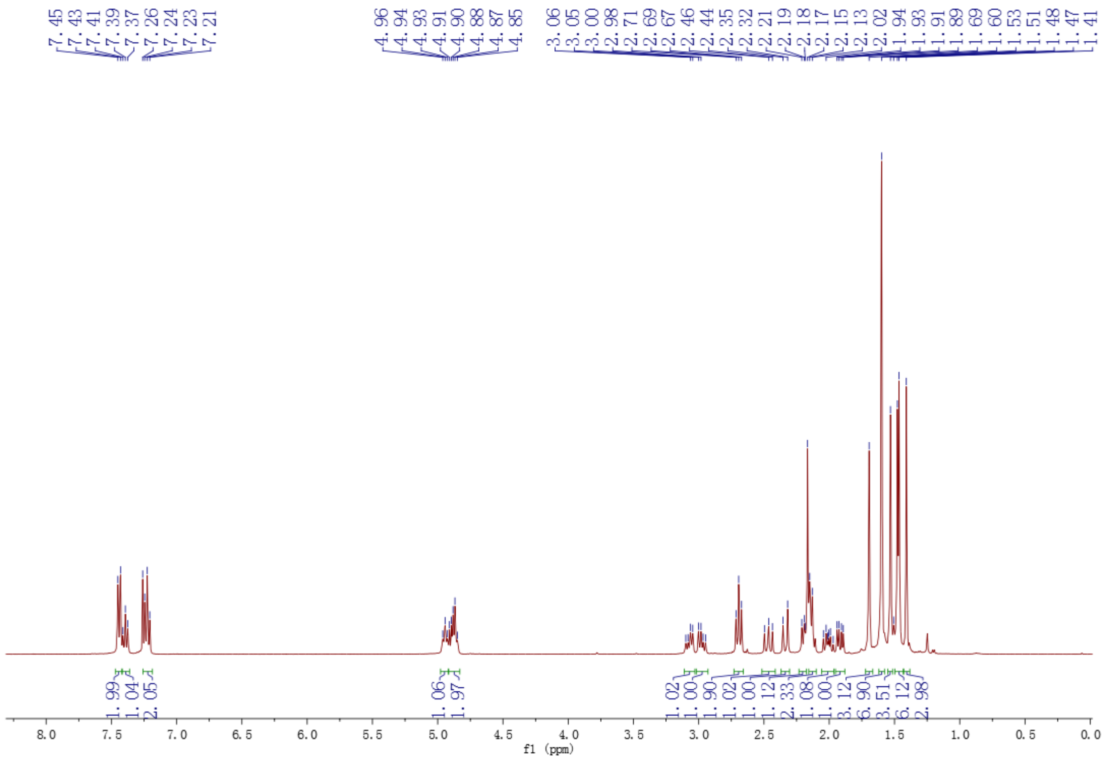

^{13}C NMR and DEPT of compound 2

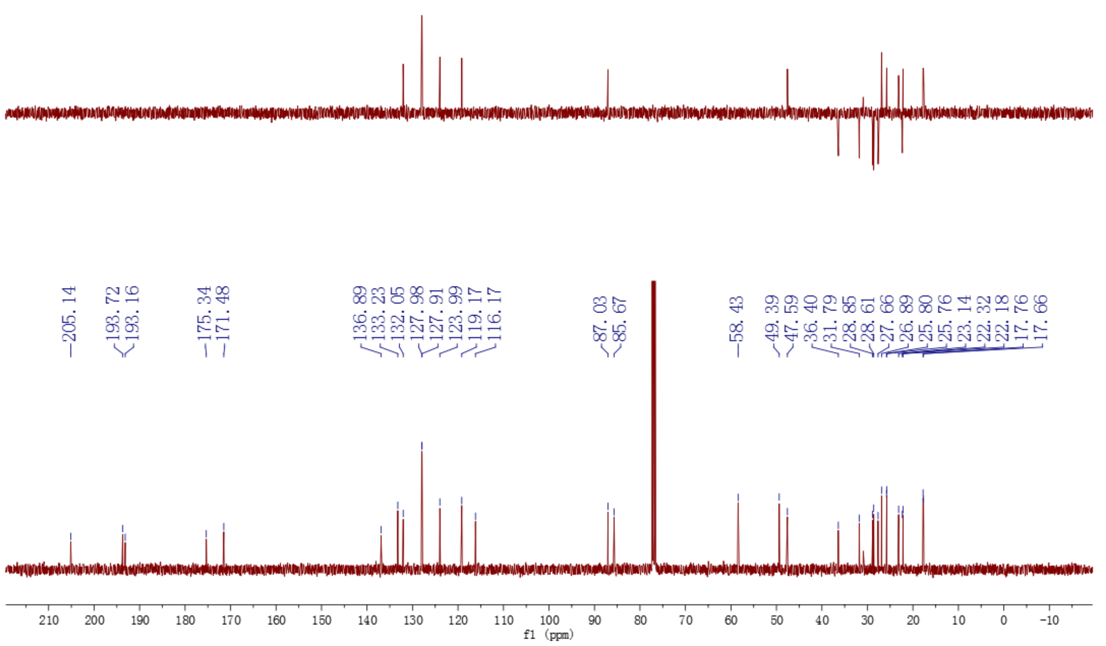

HSQC of compound 2

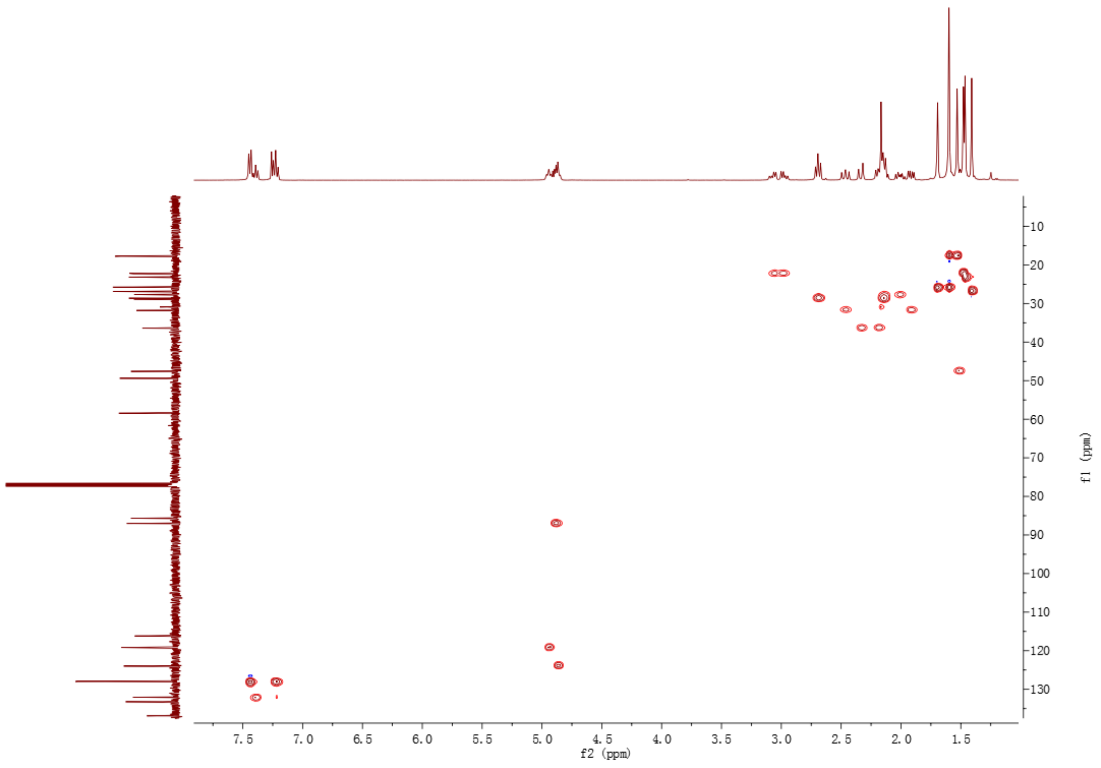

HMBC of compound 2

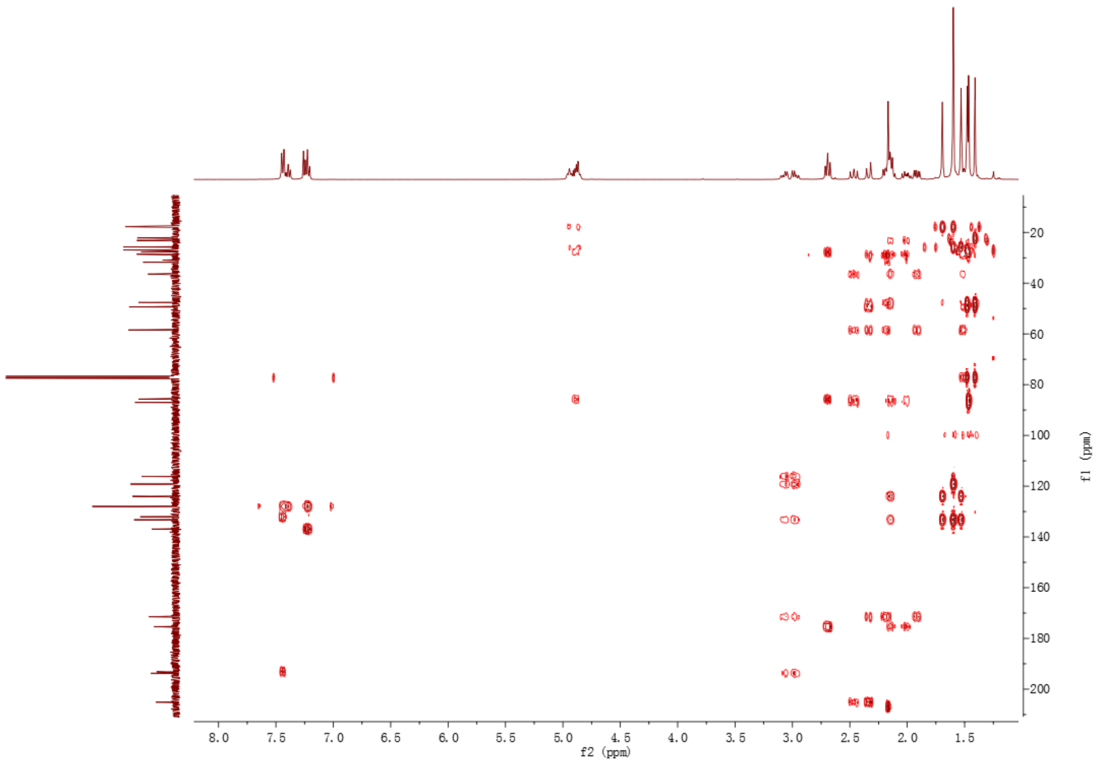

^{1}H–^{1}H COSY of compound 2

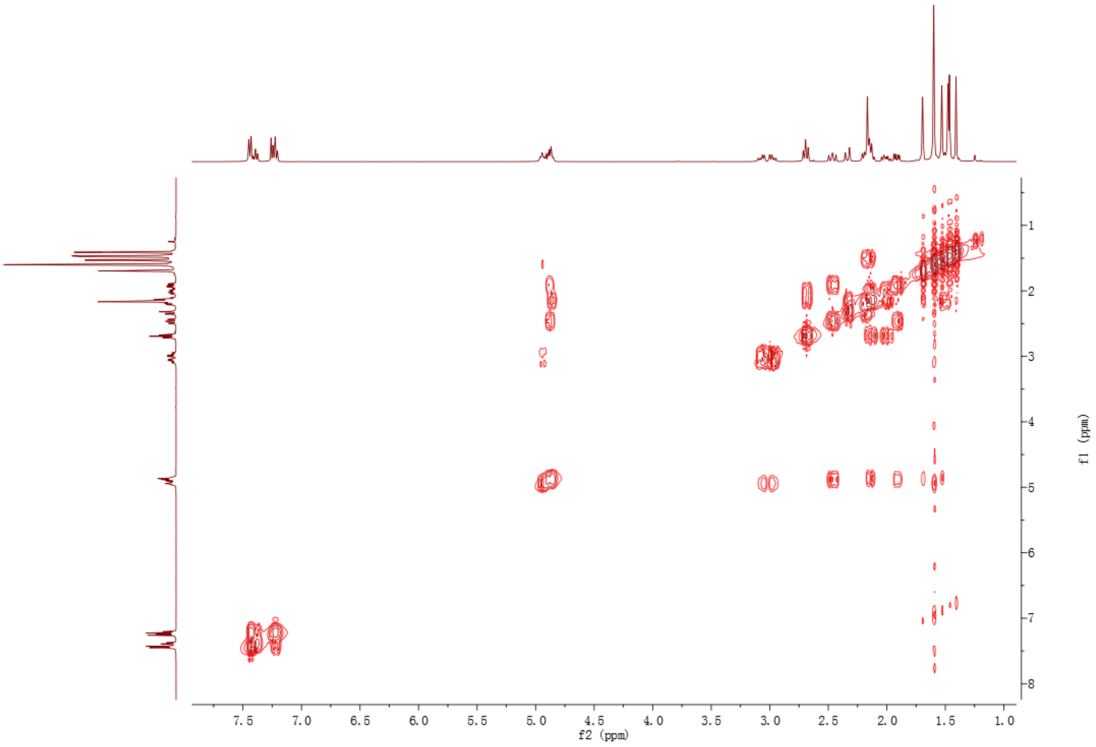

NOESY of compound 2

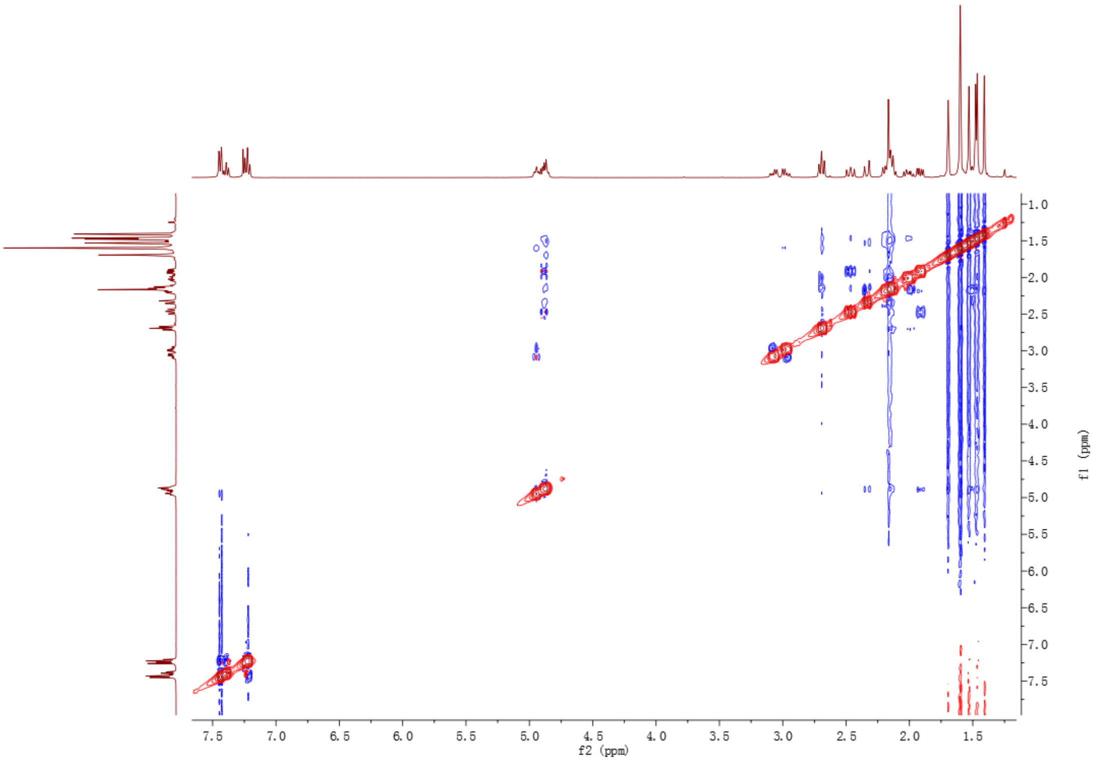

HRESIMS of compound 3

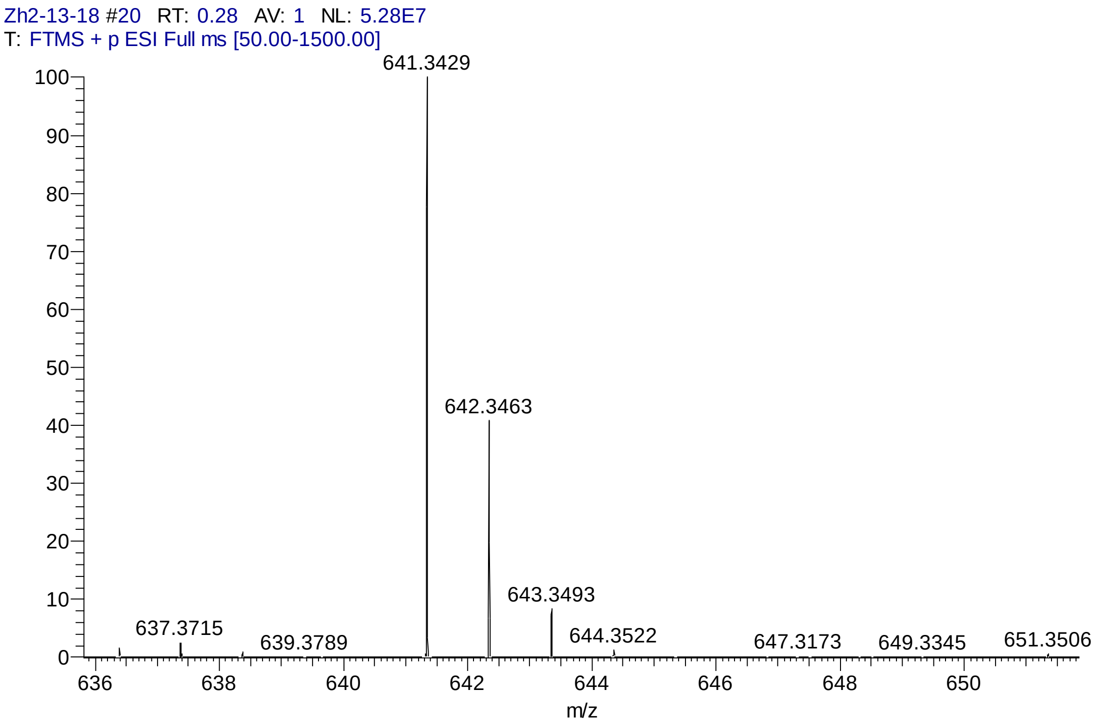

UV spectrum of compound 3

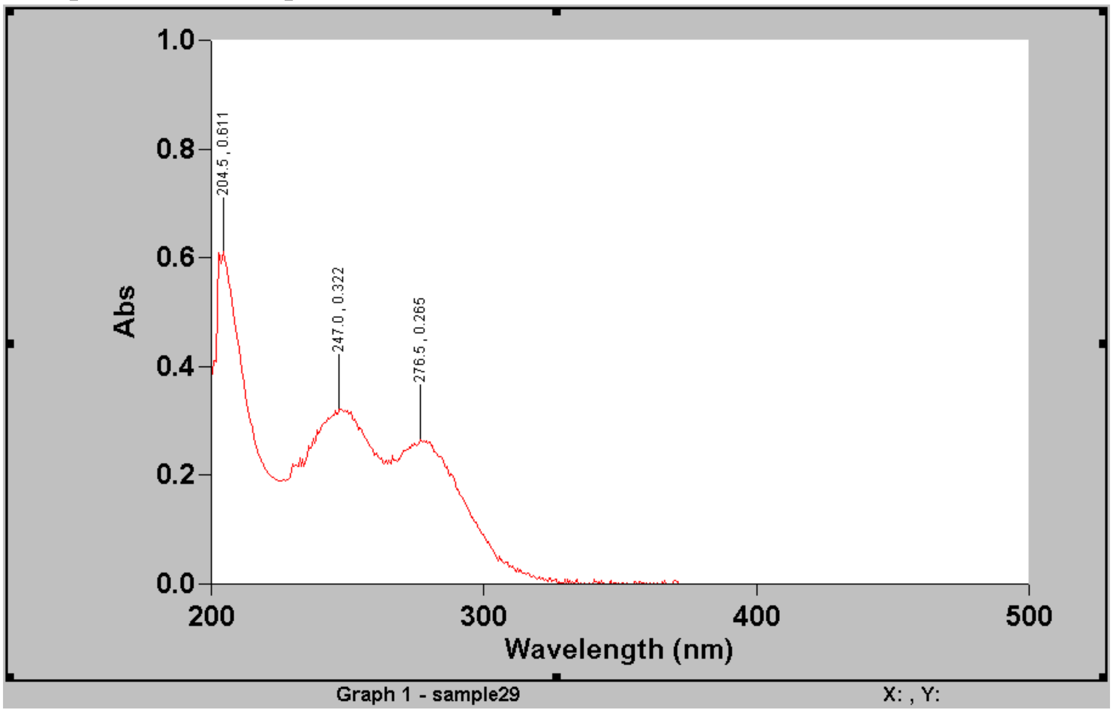

IR spectrum of compound 3

E:\同济医学院\张勇慧\20130905\ZH2-13-18.0

仪器型号：Bruker Vertex 70

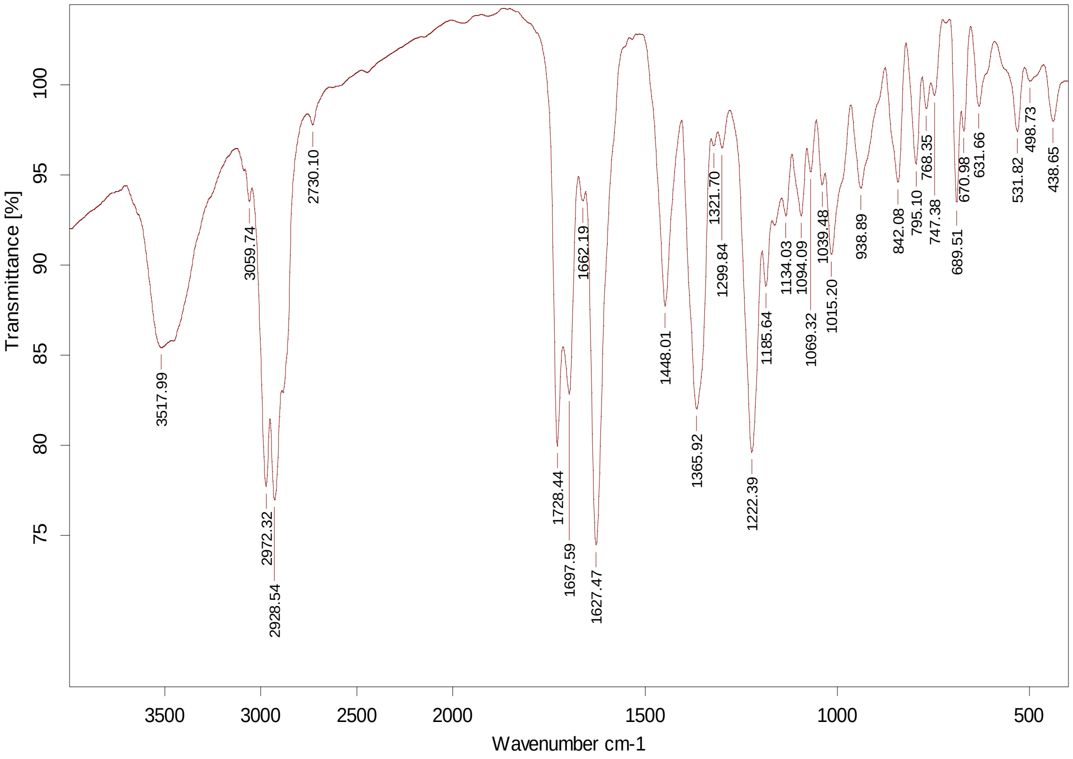

^{1}H NMR of compound 3

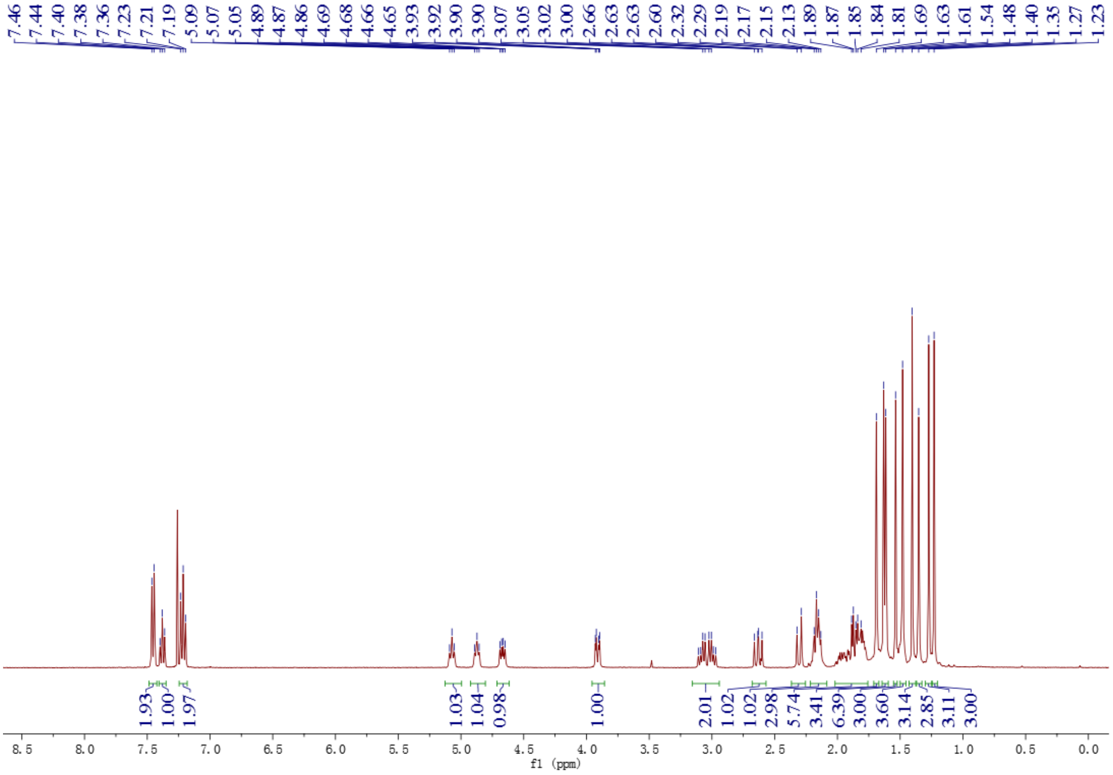

^{13}C NMR and DEPT of compound 3

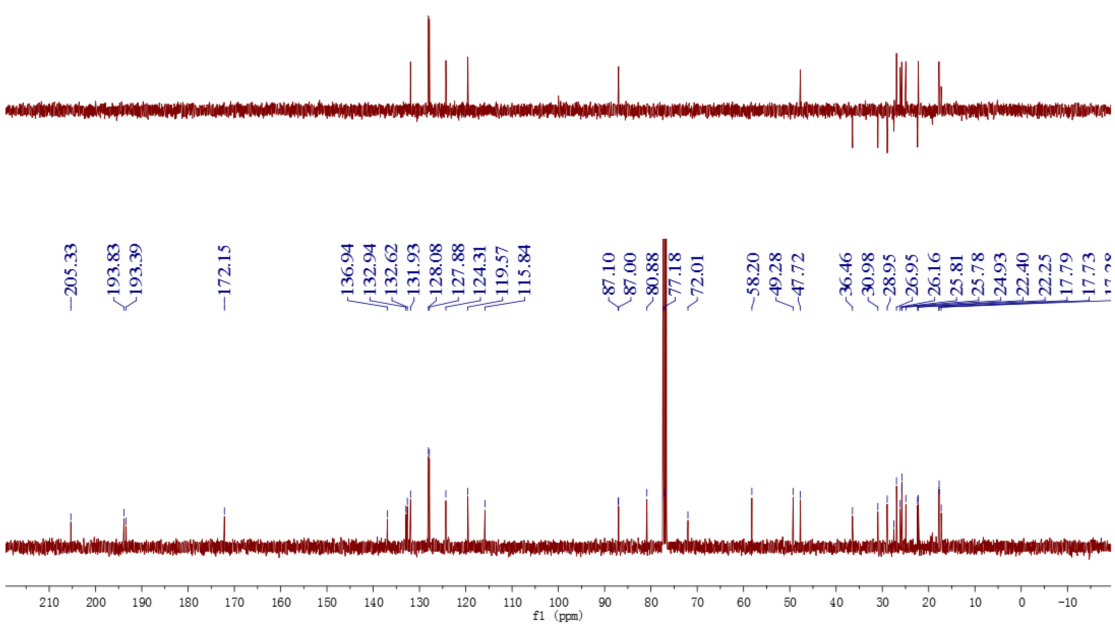

HSQC of compound 3

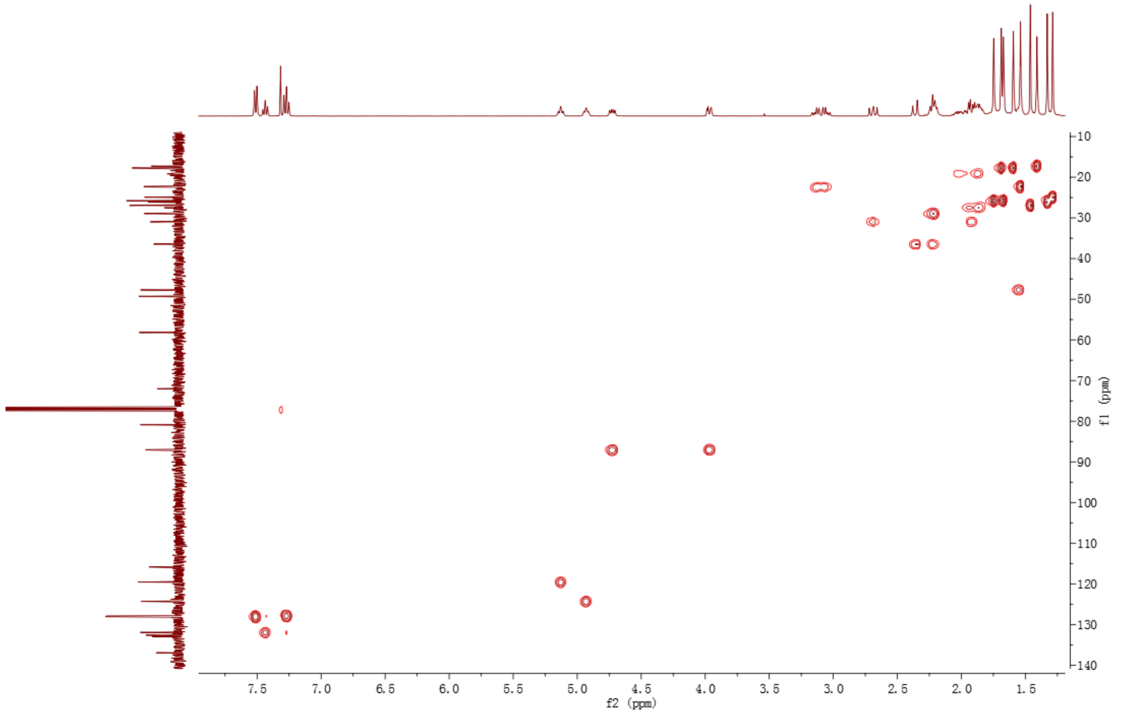

HMBC of compound 3

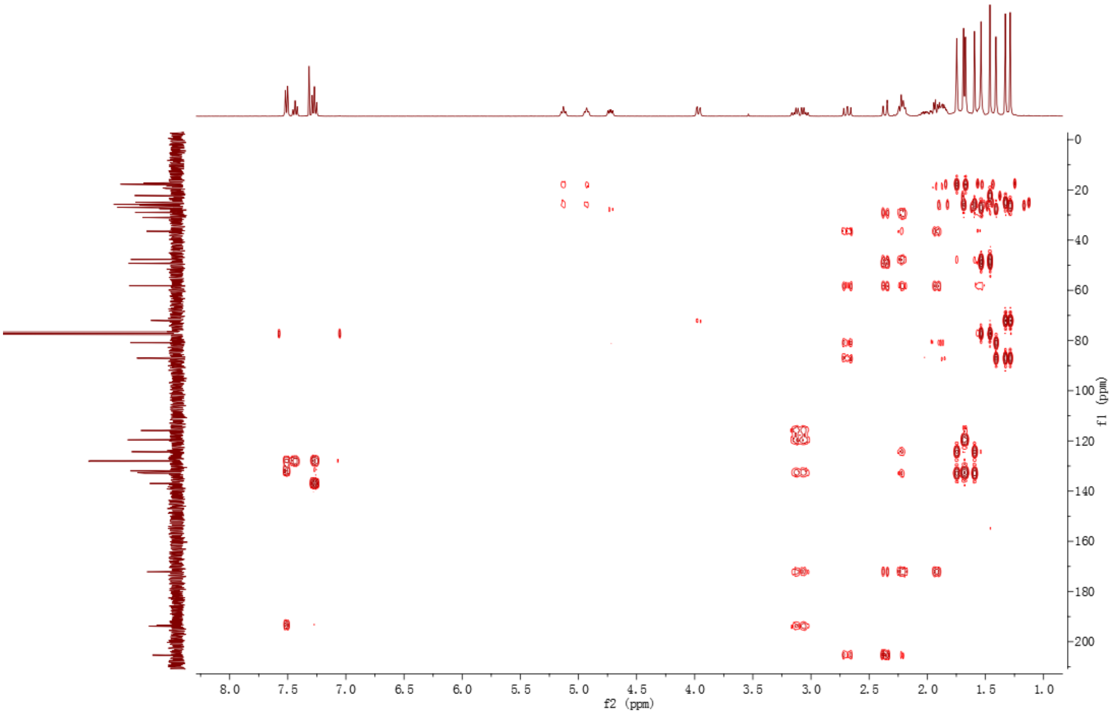

^{1}H–^{1}H COSY of compound 3

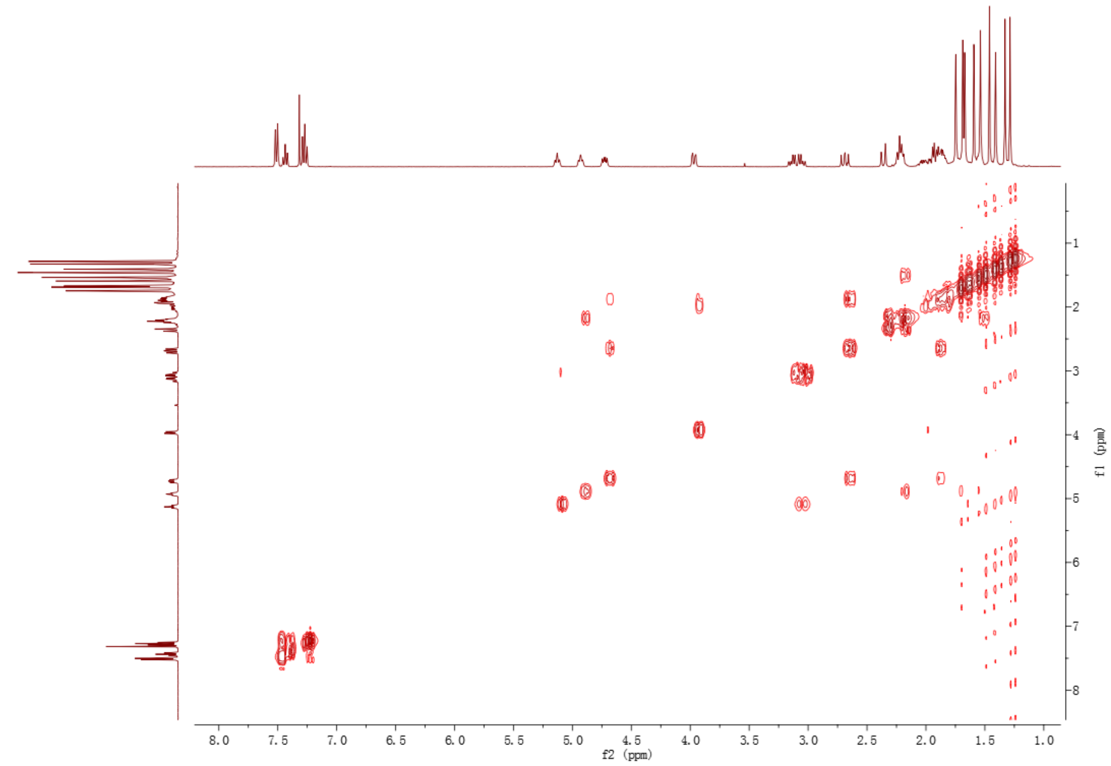

NOESY of compound 3

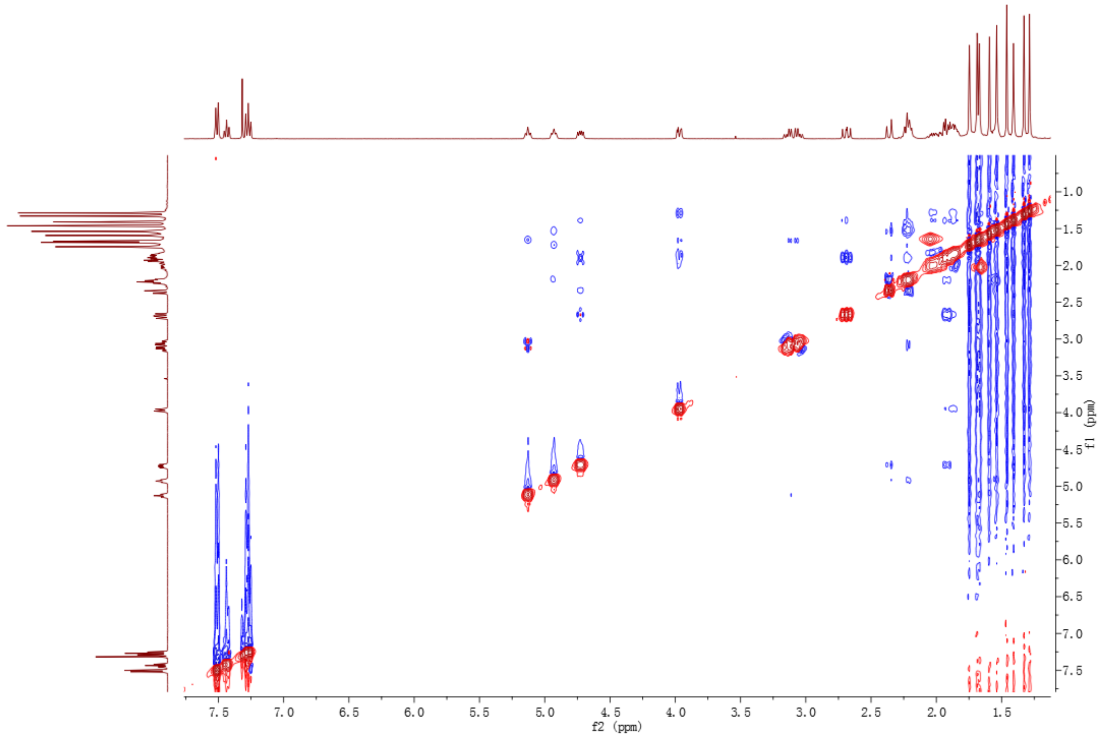

^{1}H NMR of compound 3 in C_5D_5N (600 MHz)

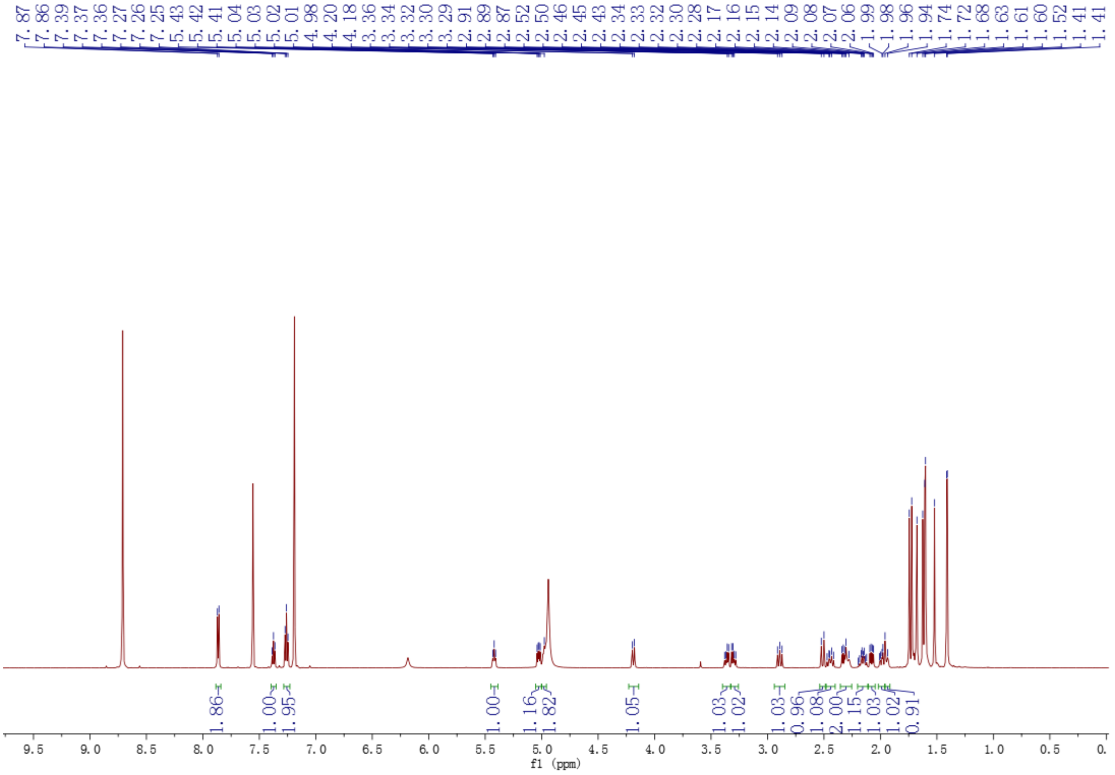

^{13}C NMR of compound 3 in C_5D_5N (600 MHz)

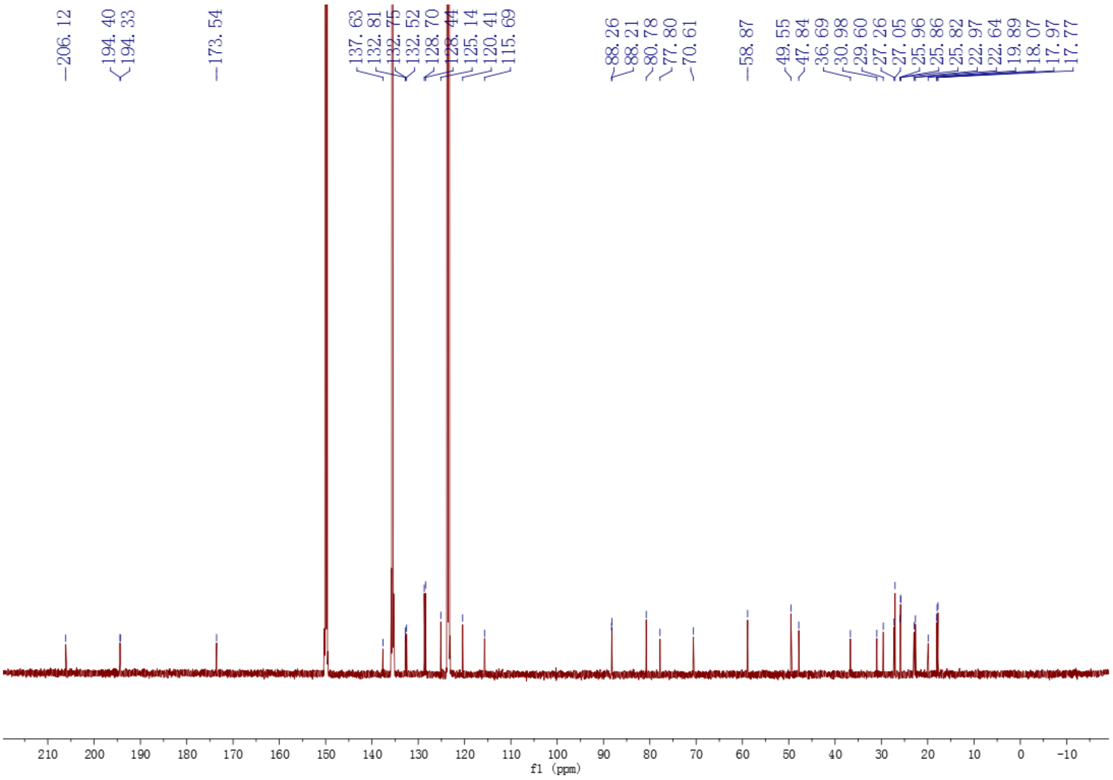

HSQC of compound 3 in C_5D_5N (600 MHz)

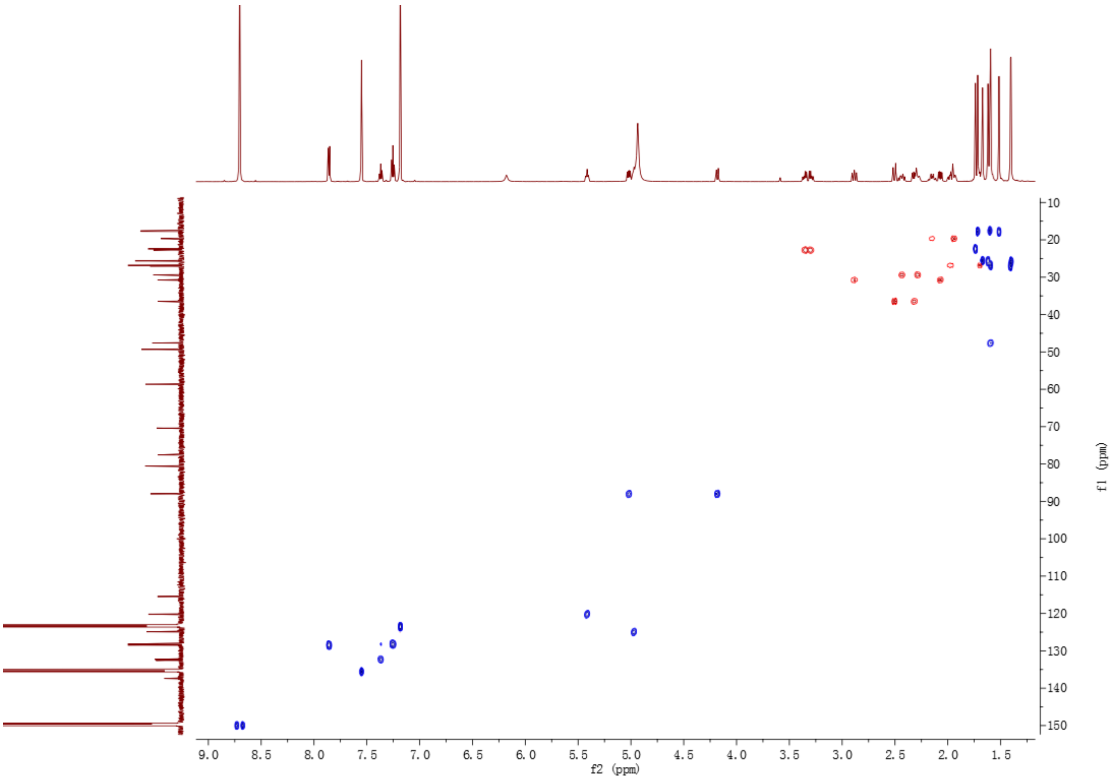

HMBC of compound 3 in C_5D_5N (600 MHz)

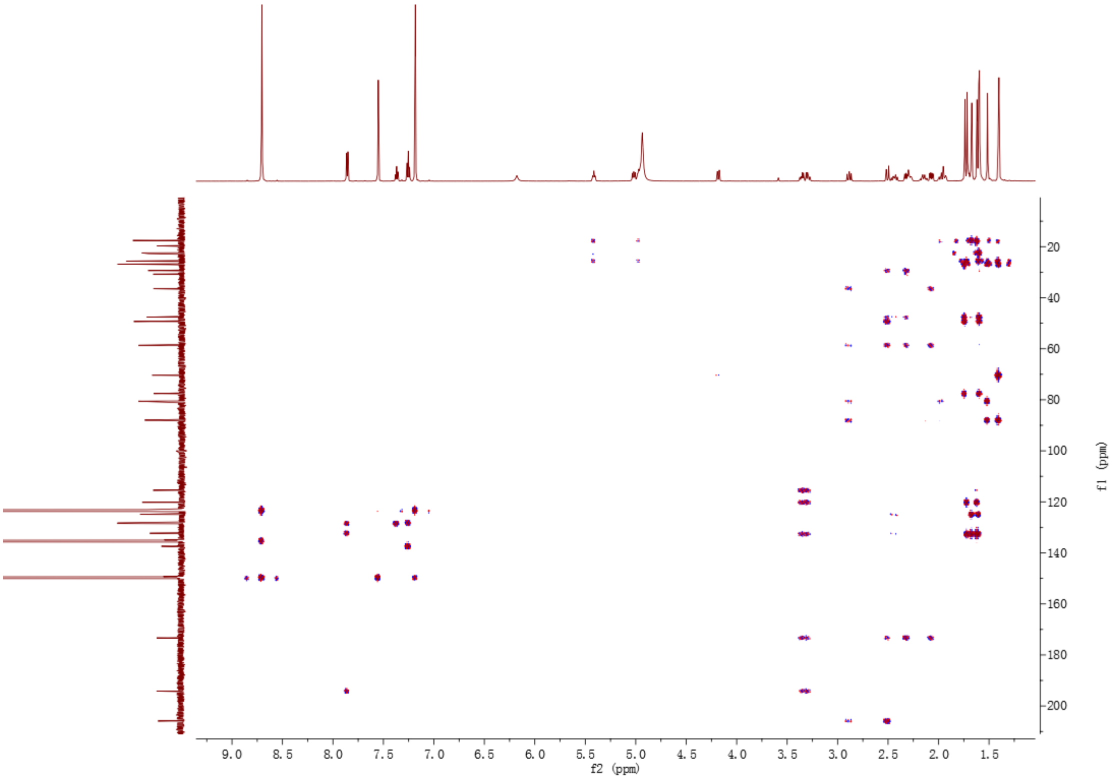

NOESY of compound 3 in C_5D_5N (600 MHz)

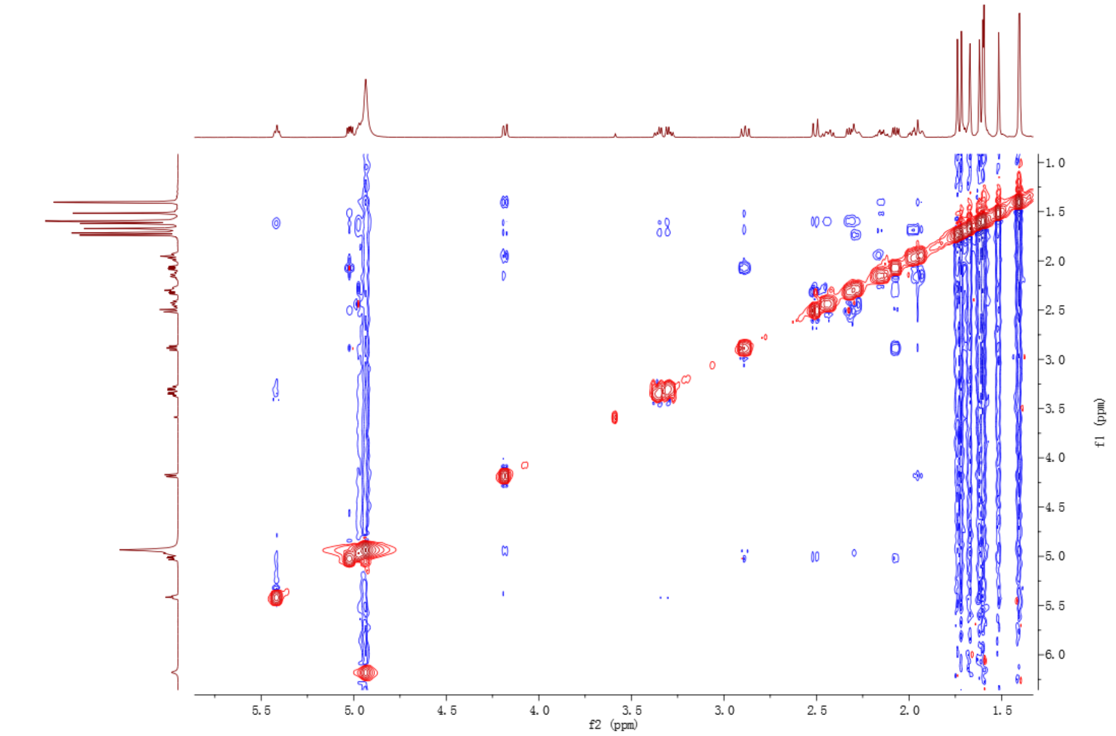

HRESIMS of compound 4

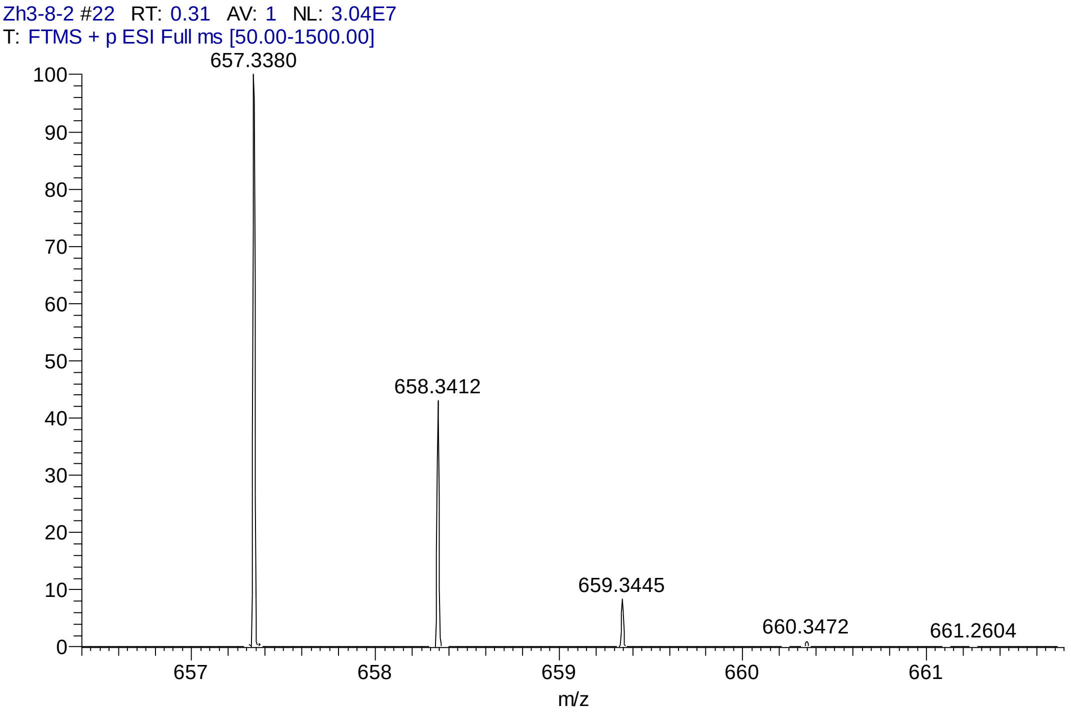

UV spectrum of compound 4

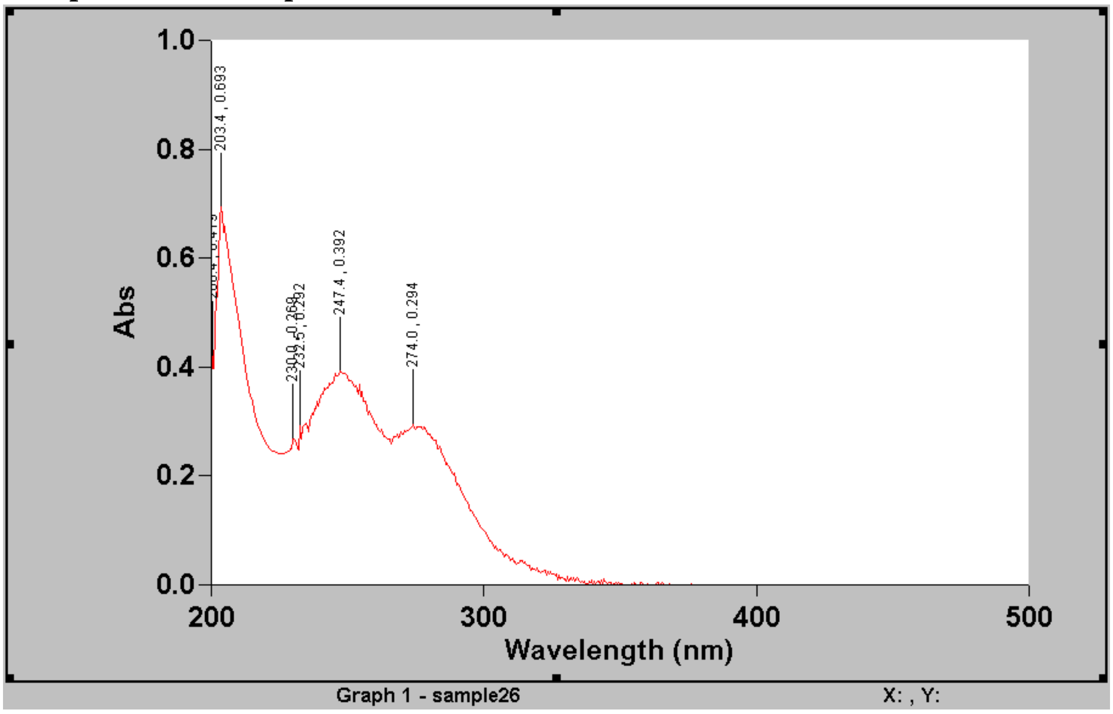

IR spectrum of compound 4

E:\同济医学院\张勇慧\20130905\ZH3-8-2.1

仪器型号：Bruker Vertex 70

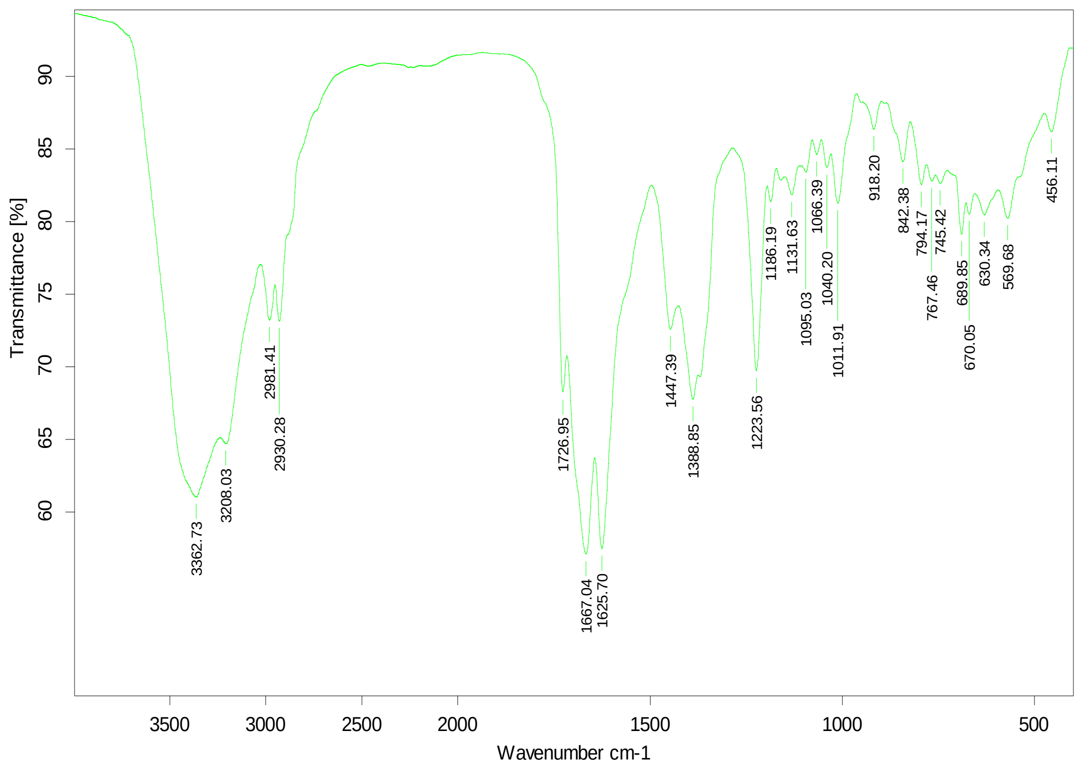

^{1}H NMR of compound 4

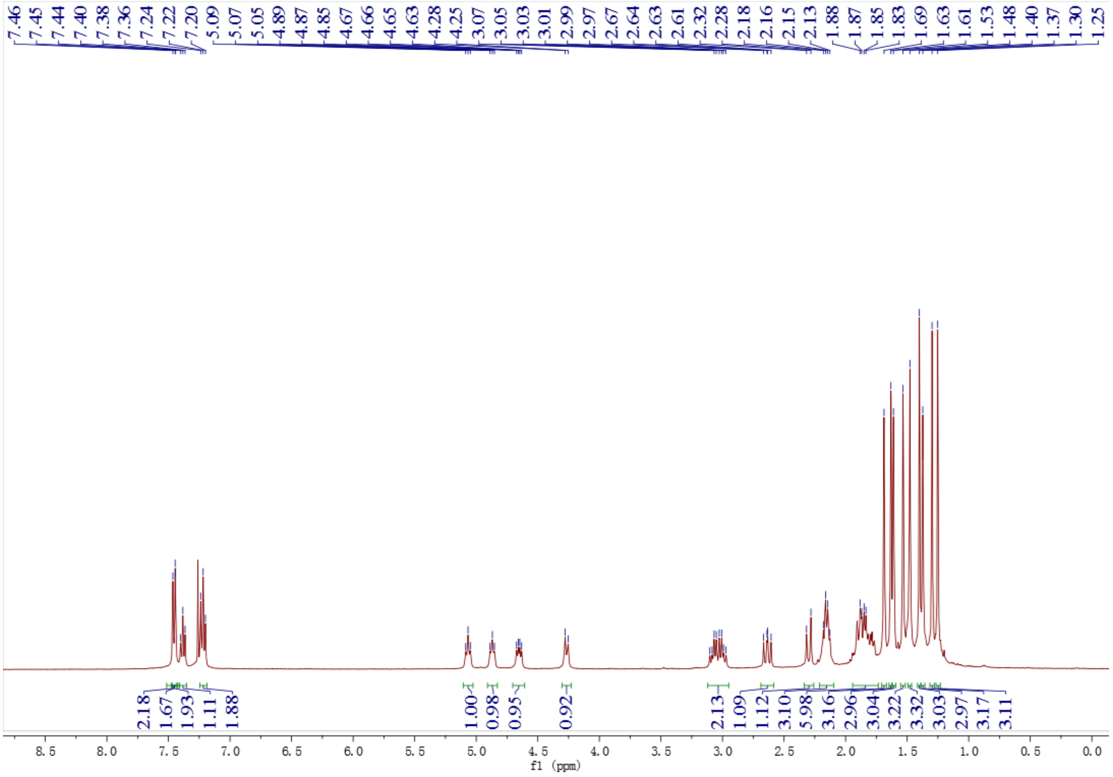

^{13}C NMR and DEPT of compound 4

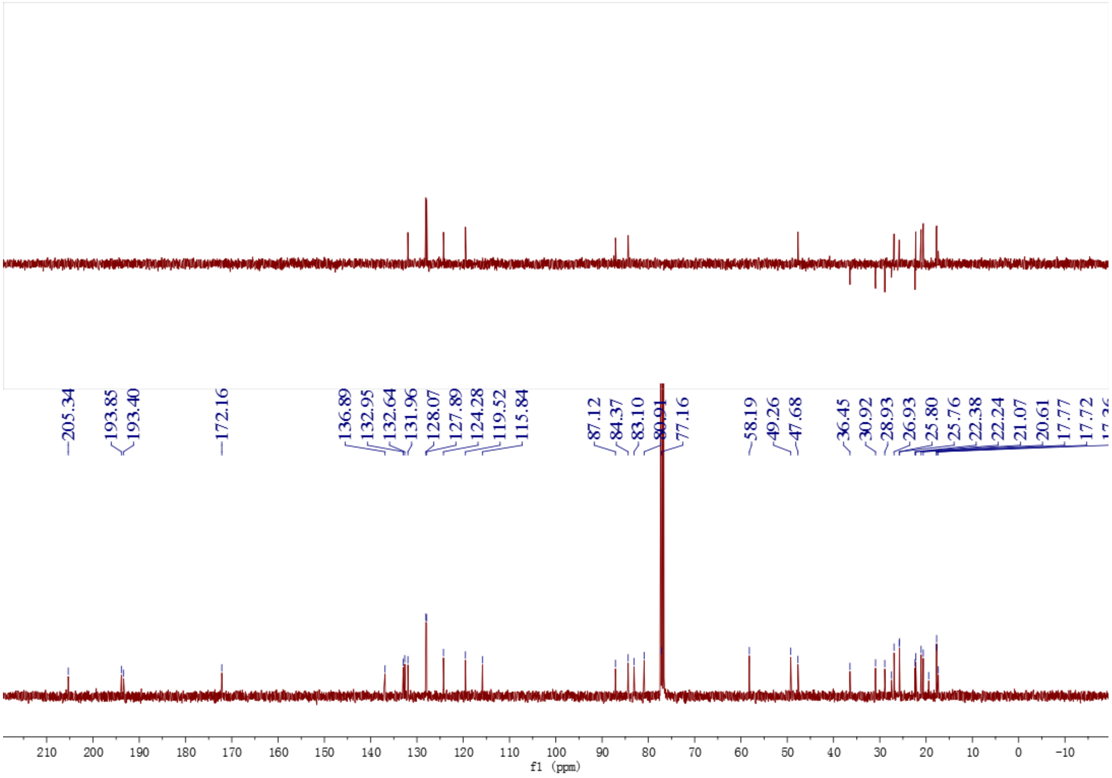

HSQC of compound 4

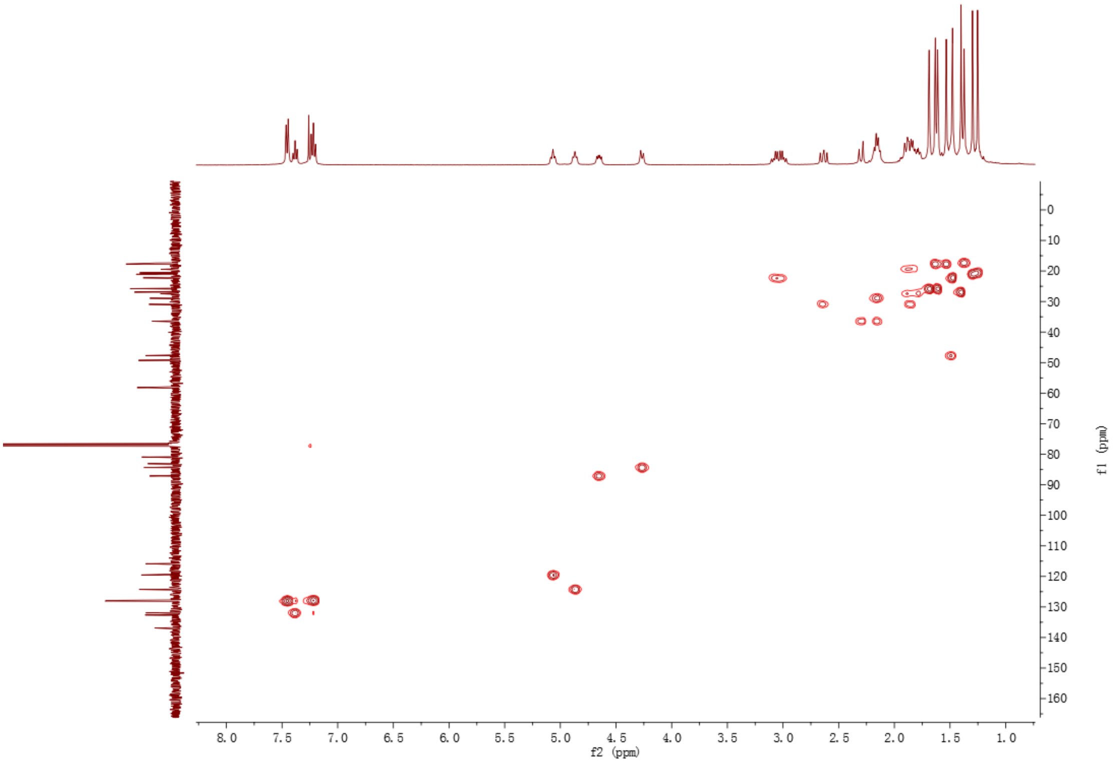

HMBC of compound 4

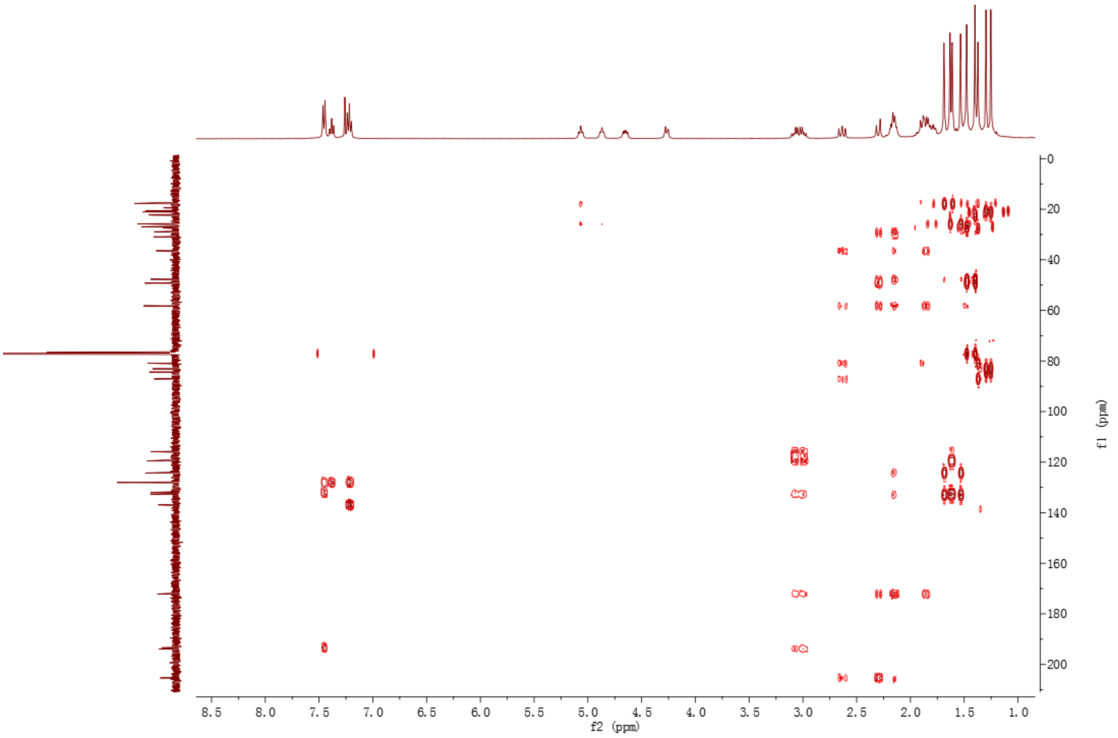

^{1}H–^{1}H COSY of compound 4

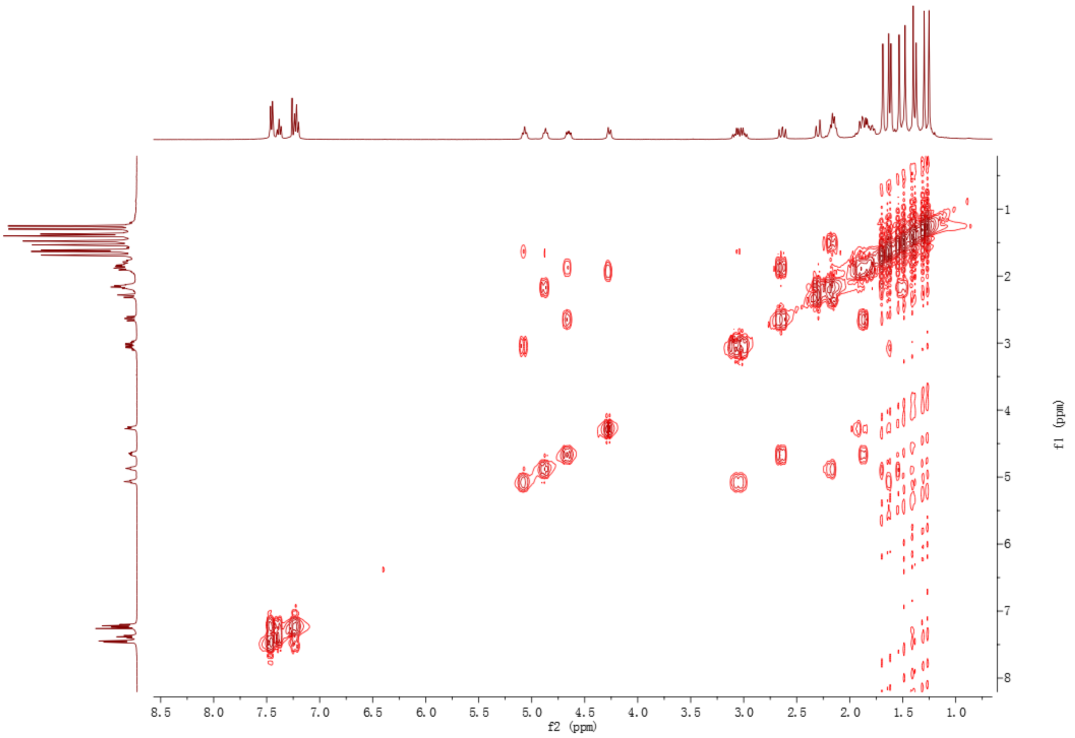

NOESY of compound 4

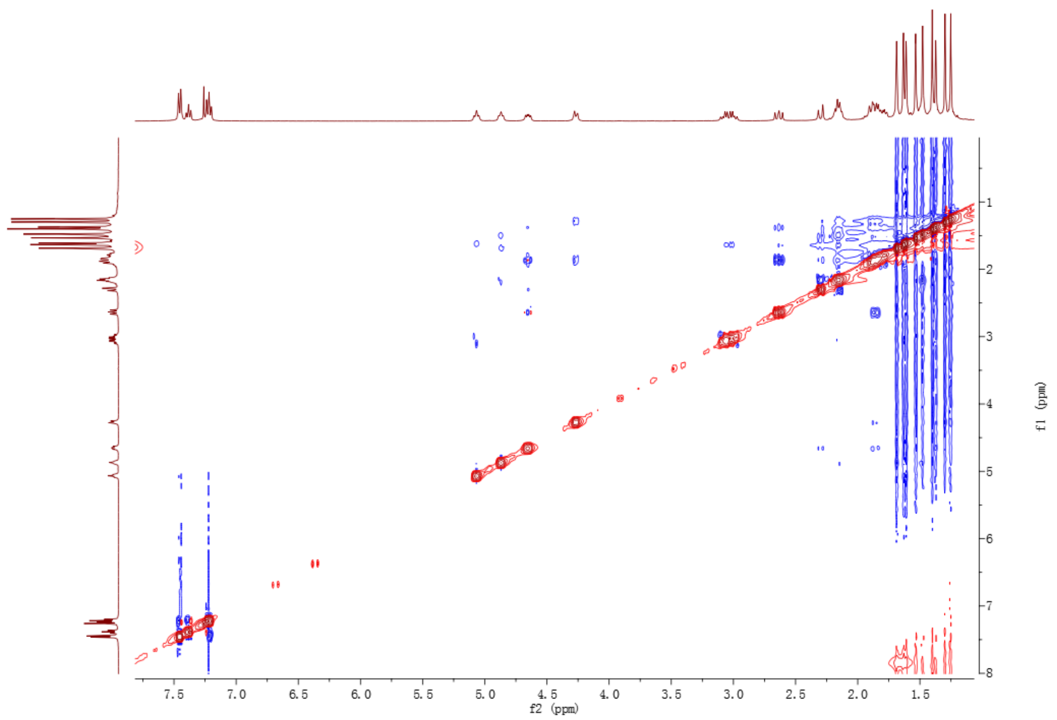

HRESIMS of compound 5

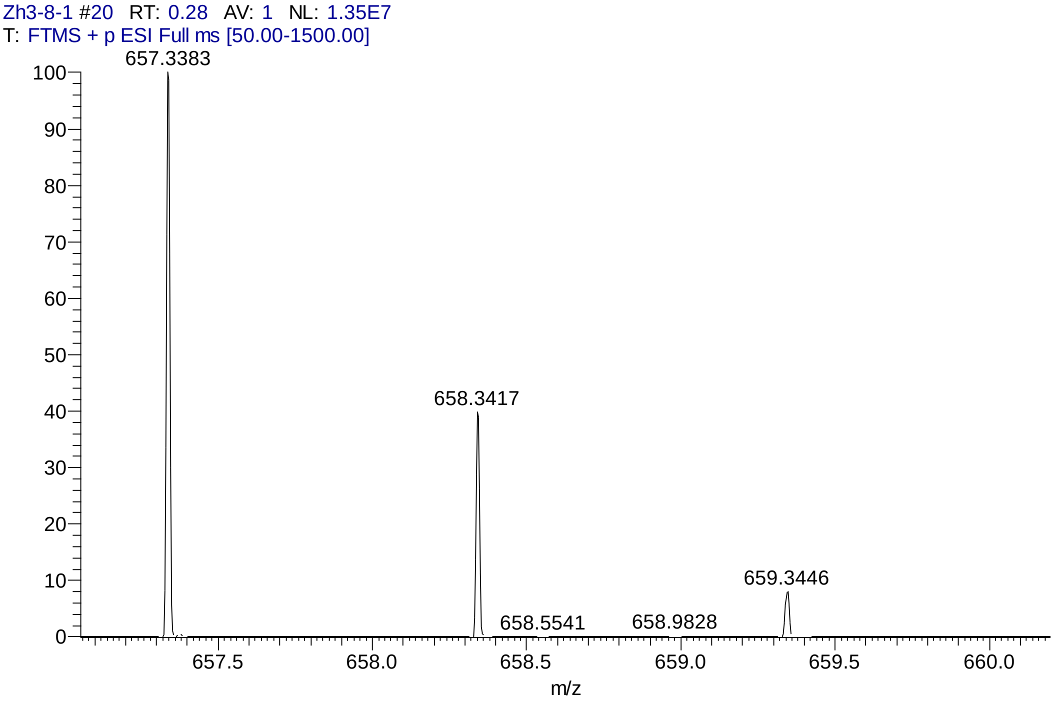

UV spectrum of compound 5

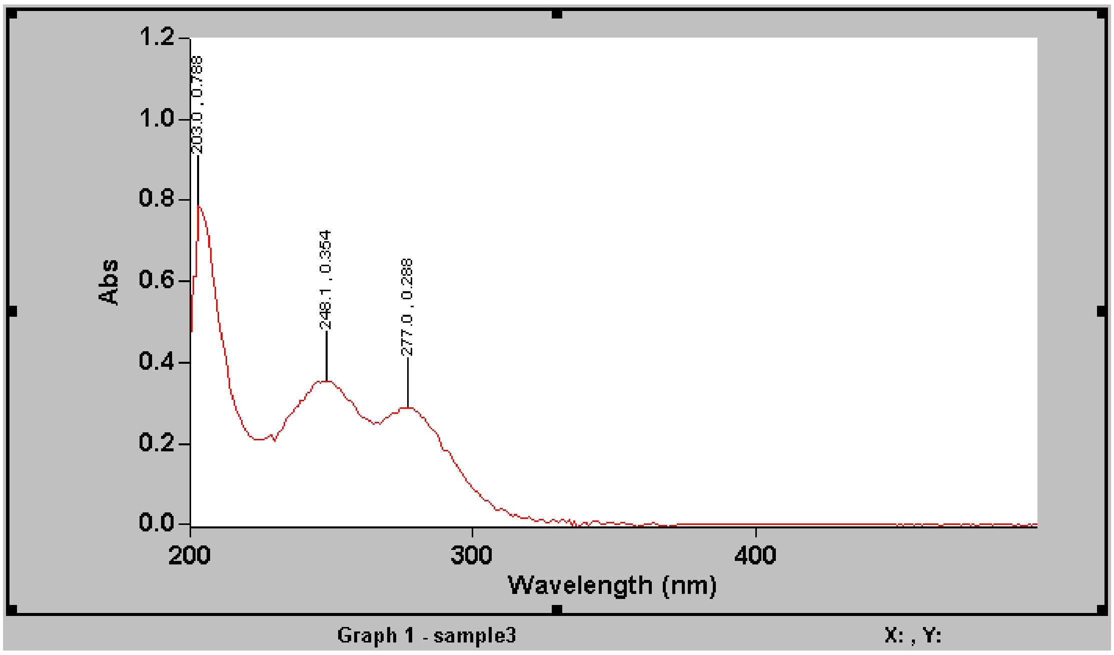

IR spectrum of compound 5

E:\同济医学院\张勇慧\20130905\ZH3-8-2.0

仪器型号：Bruker Vertex 70

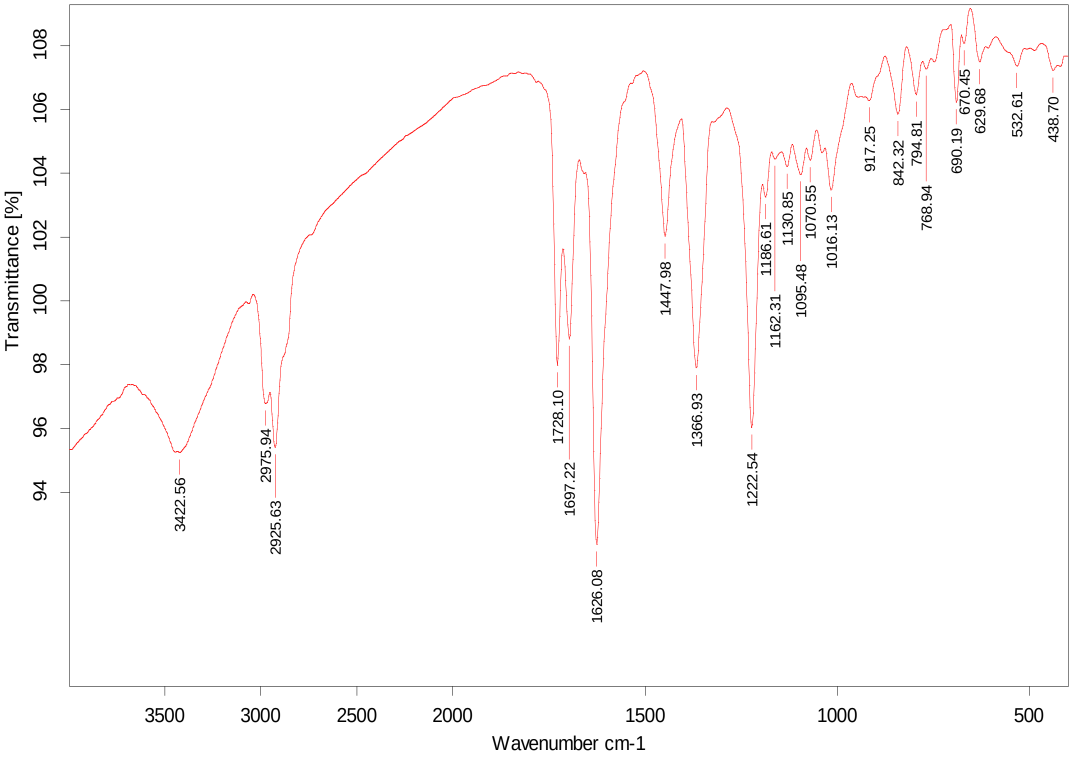

^{1}H NMR of compound 5

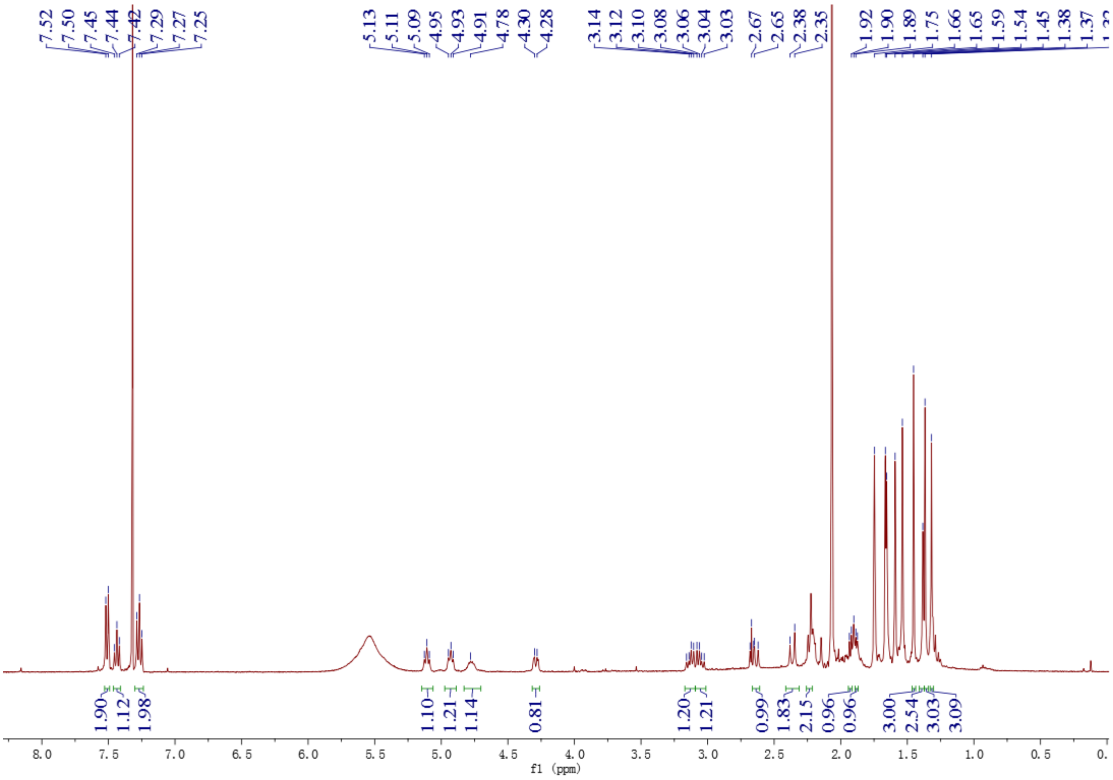

^{13}C NMR and DEPT of compound 5

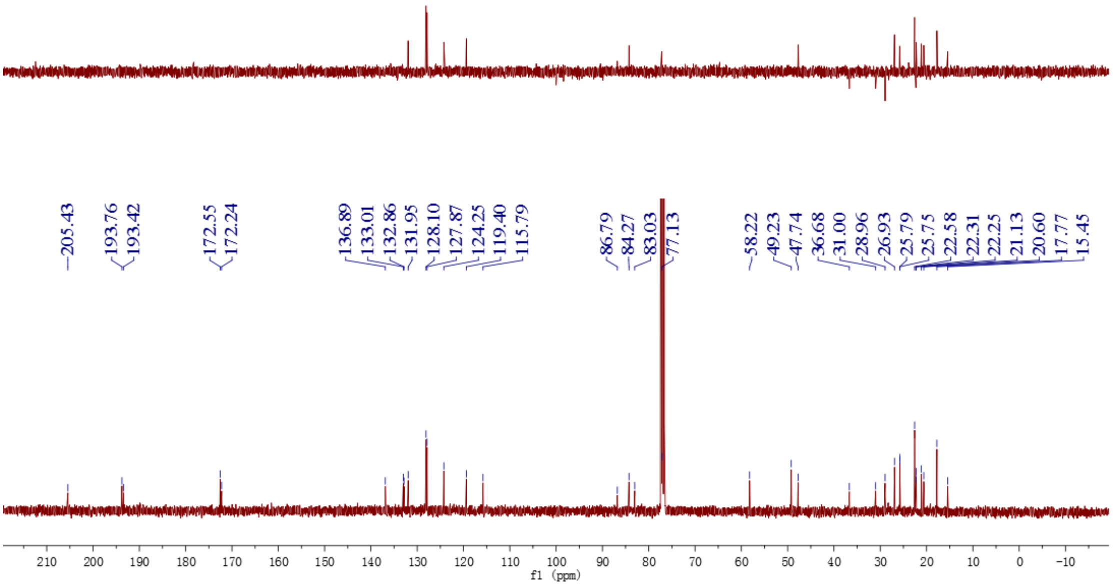

HSQC of compound 5

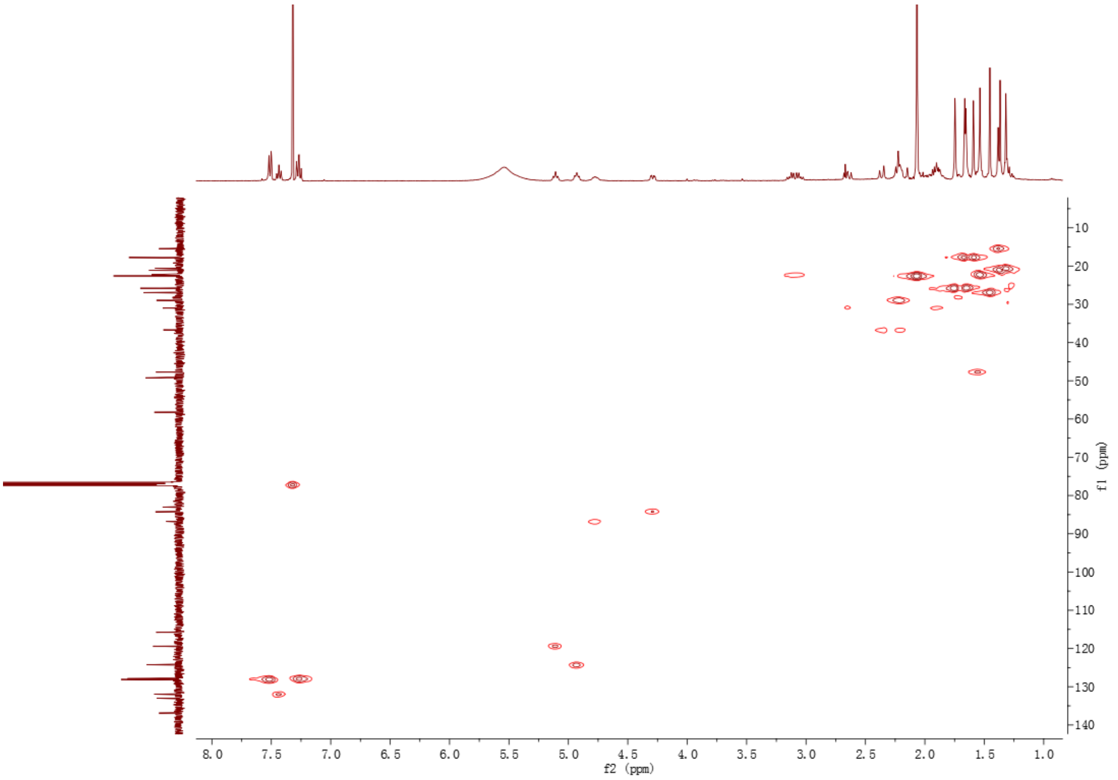

HMBC of compound 5

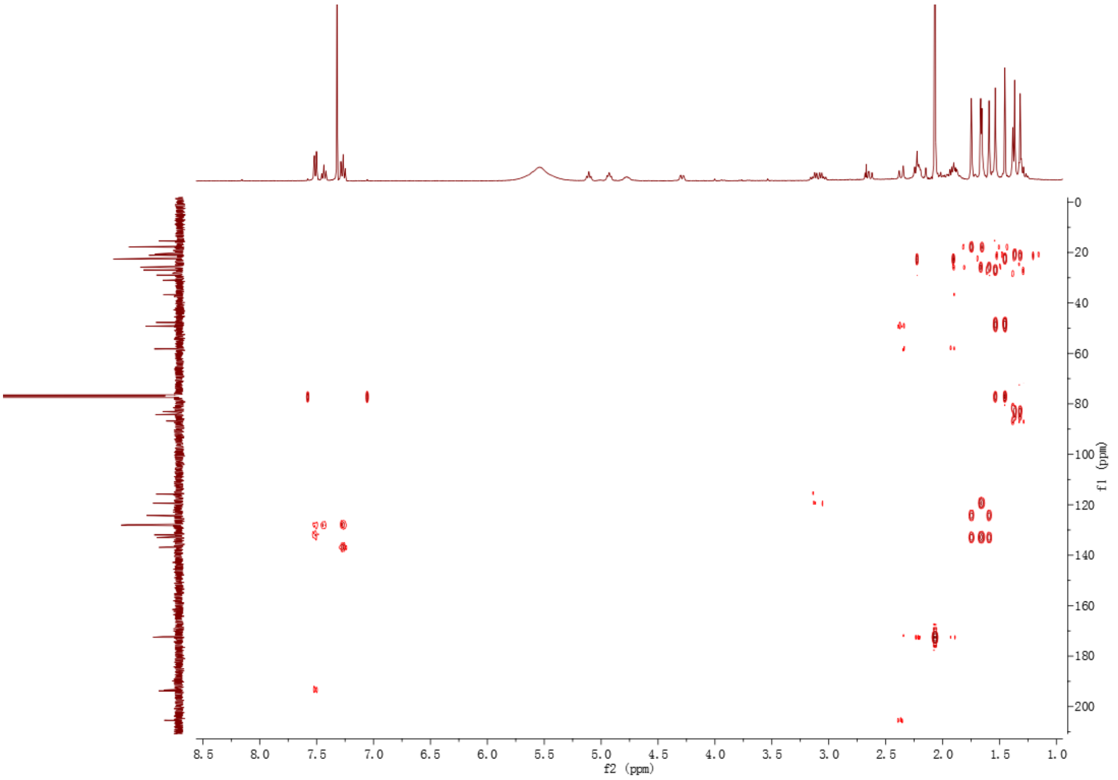

^{1}H–^{1}H COSY of compound 5

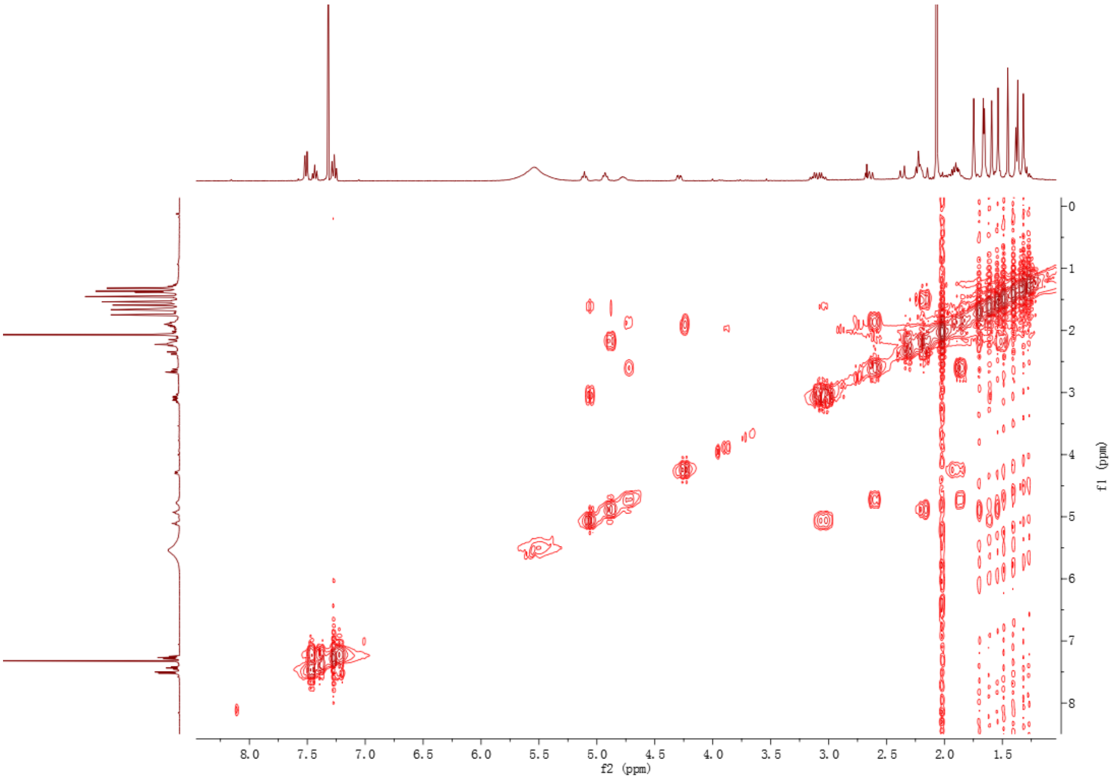

NOESY of compound 5

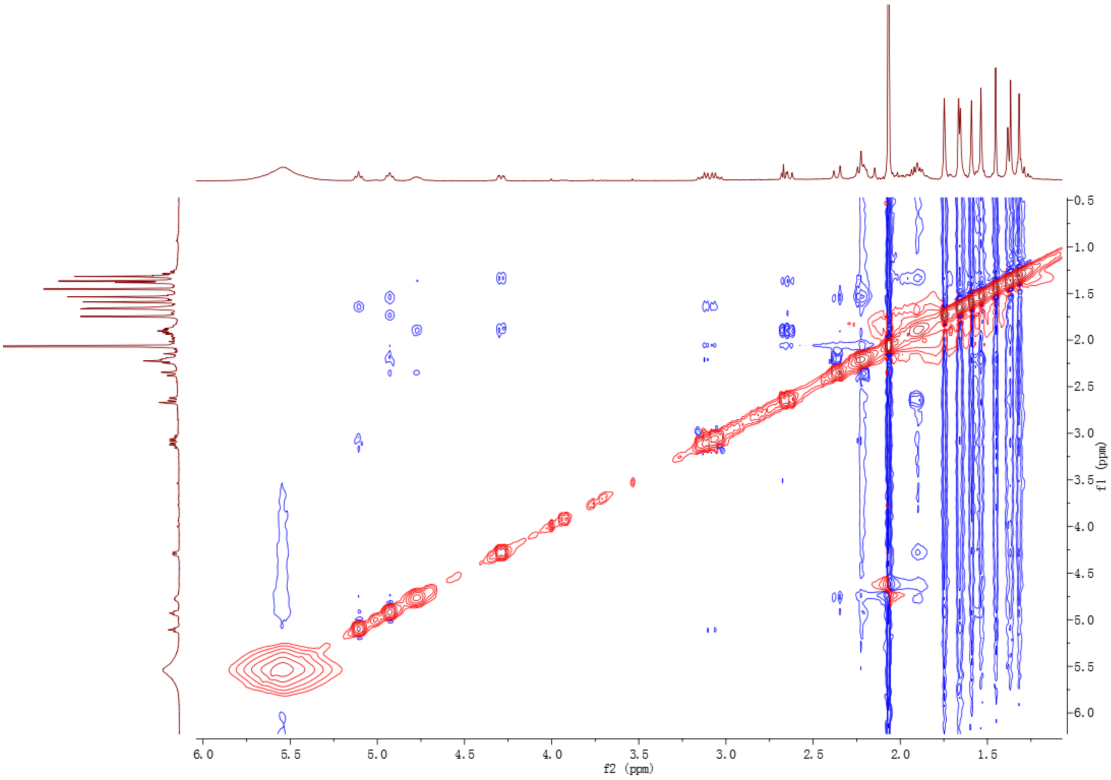

HRESIMS of compound 6

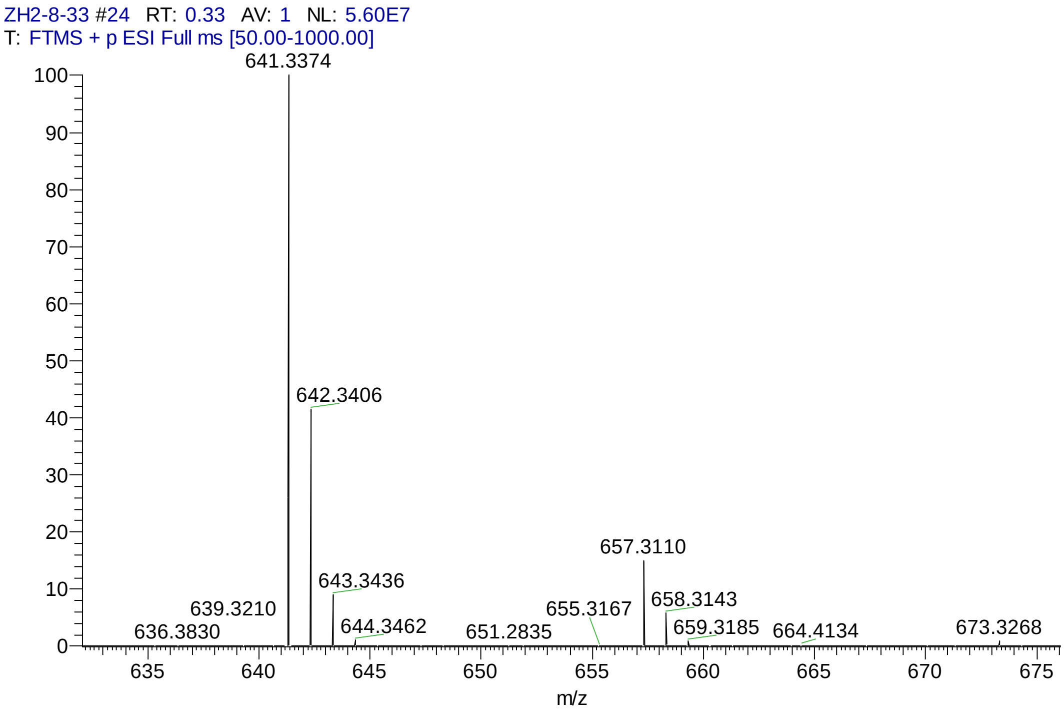

UV spectrum of compound 6

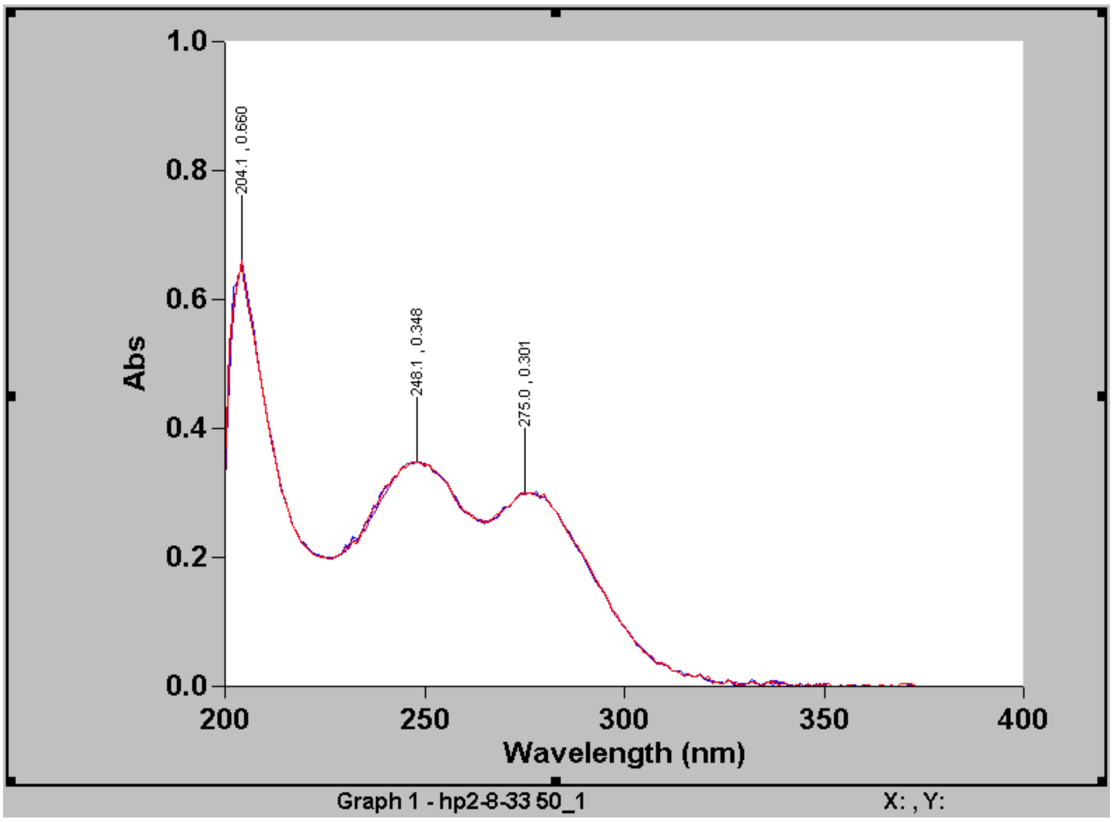

IR spectrum of compound 6

E:\同济医学院\张勇慧\20130314\zh2-8-33.0

仪器型号：Bruker Vertex 70

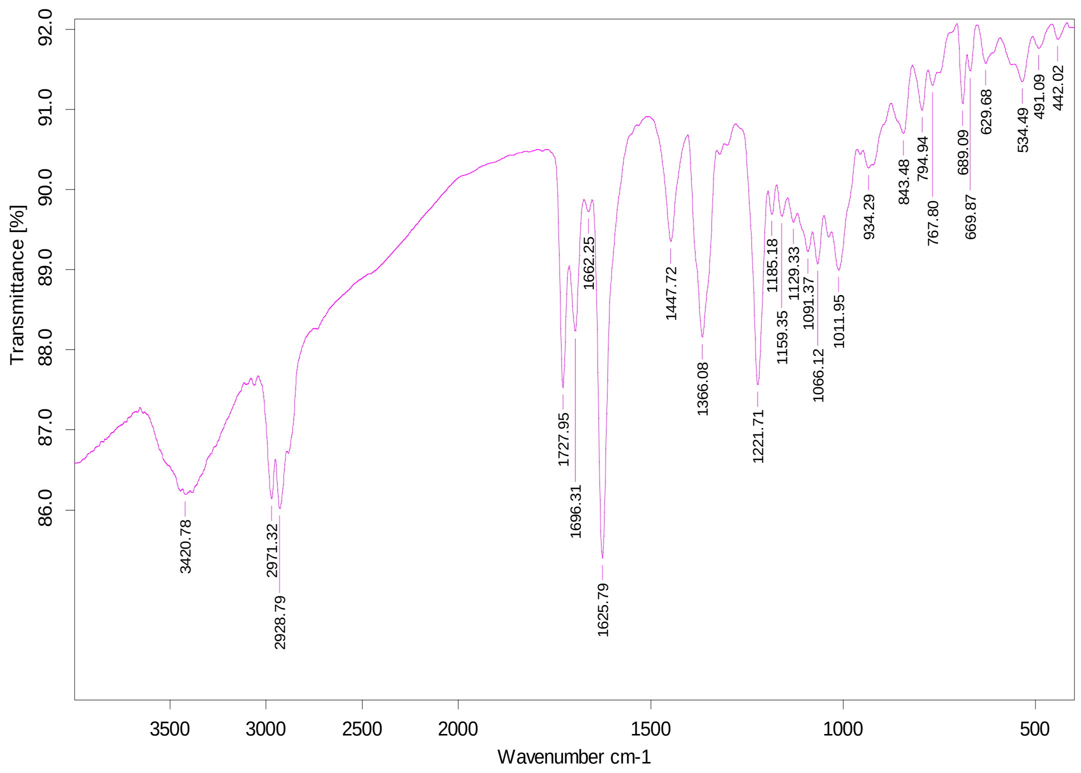

^{1}H NMR of compound 6

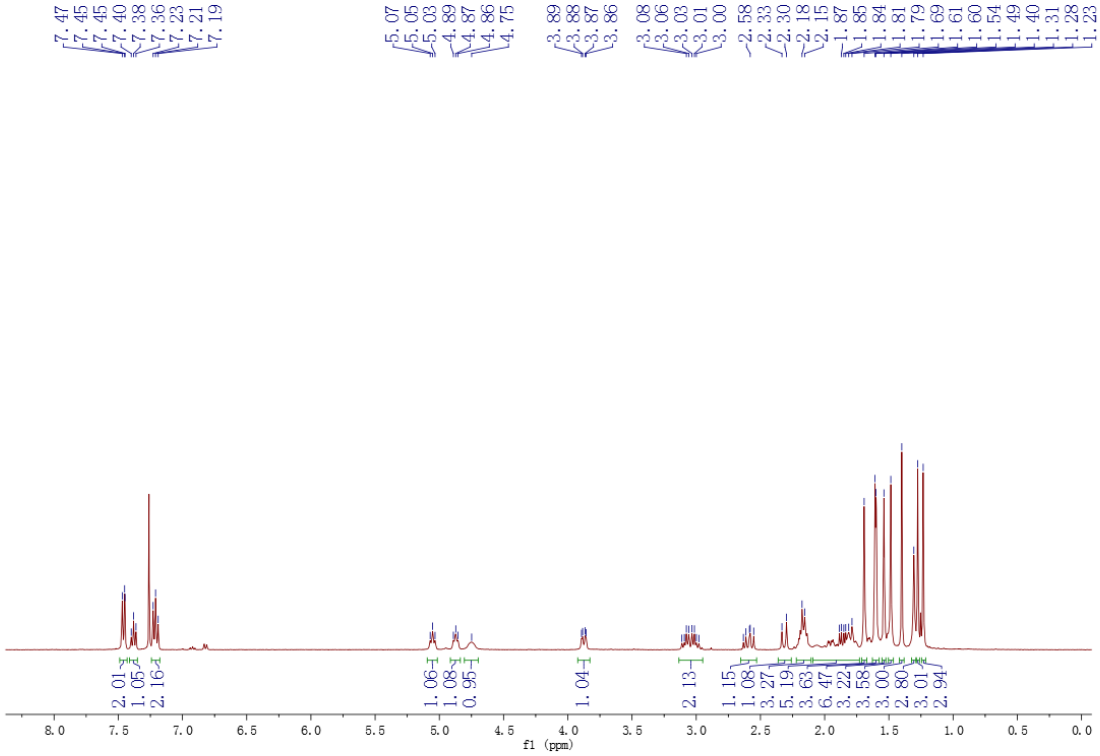

^{13}C NMR and DEPT of compound 6

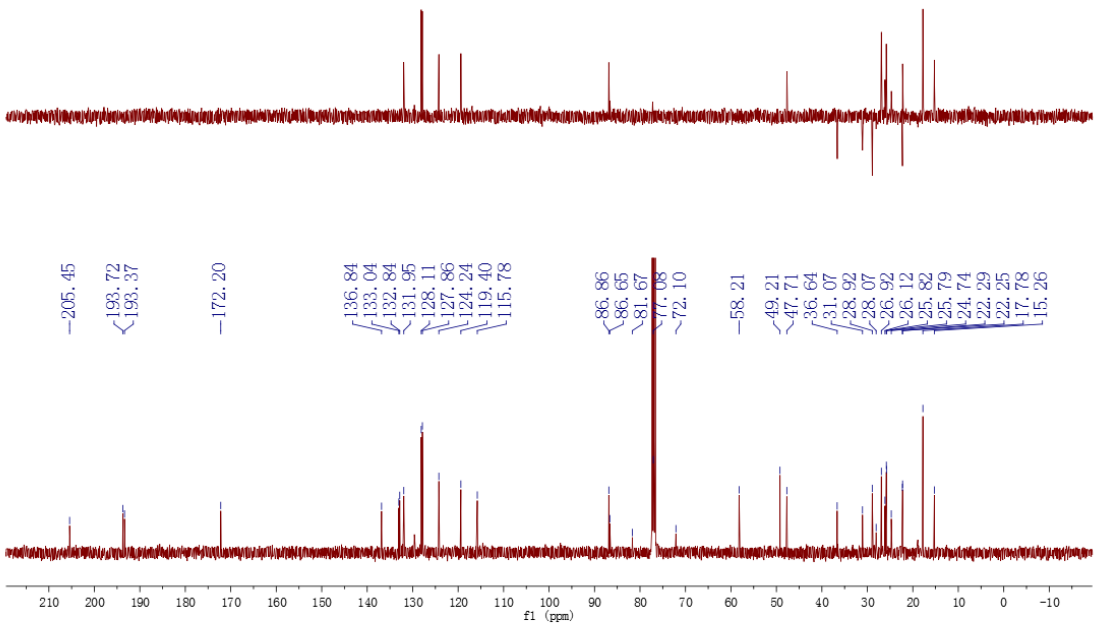

HSQC of compound 6

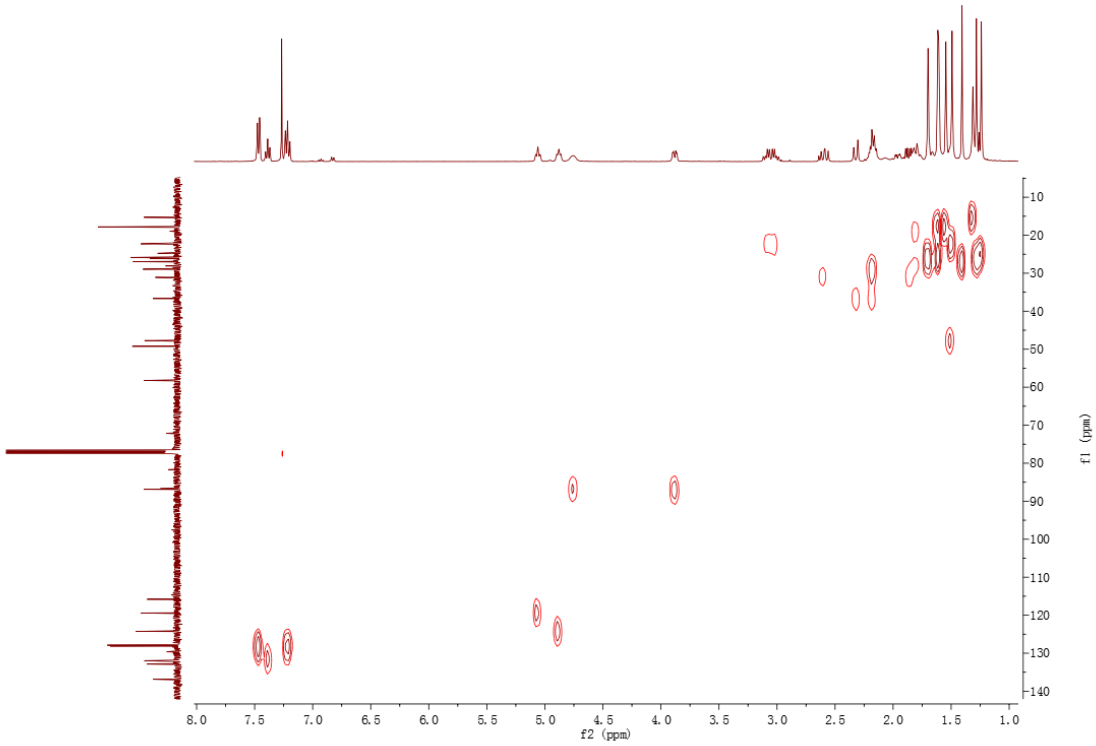

HMBC of compound 6

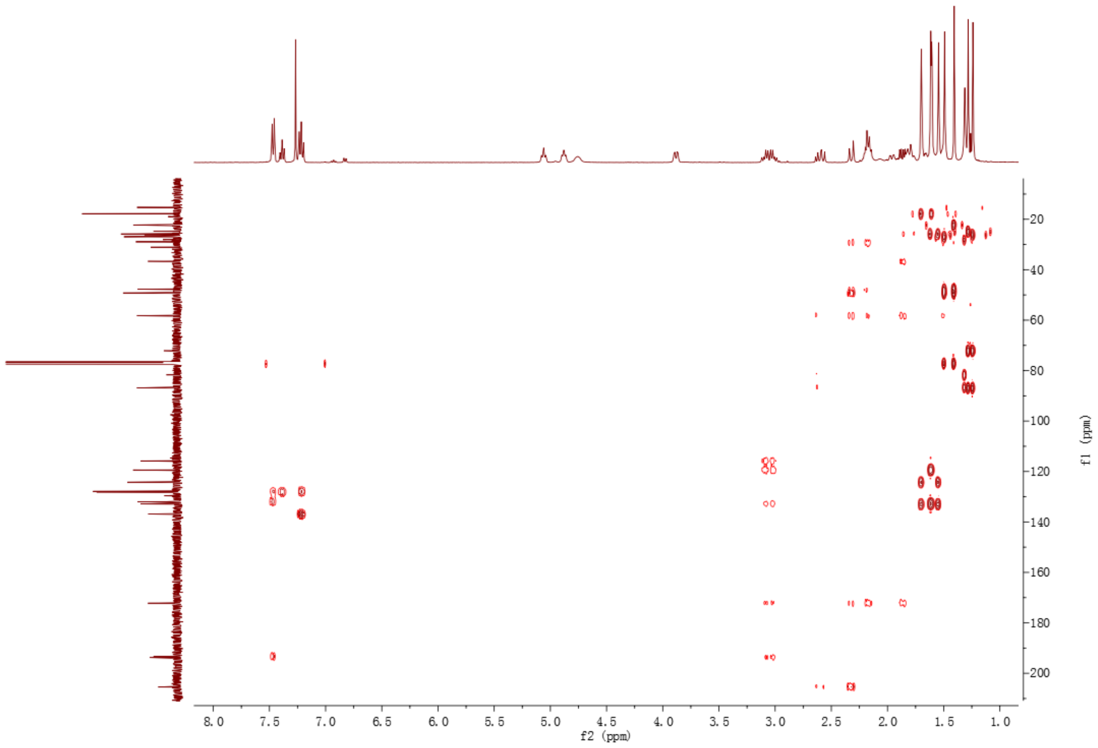

^1H–^1H COSY of compound 6

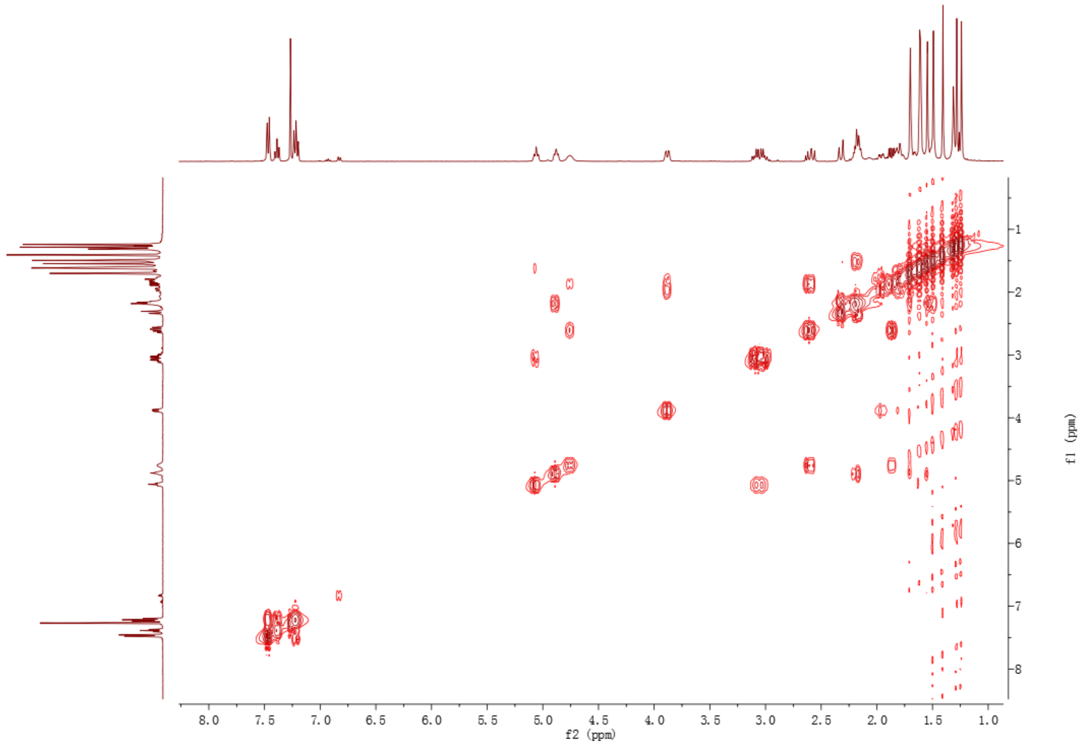

NOESY of compound 6

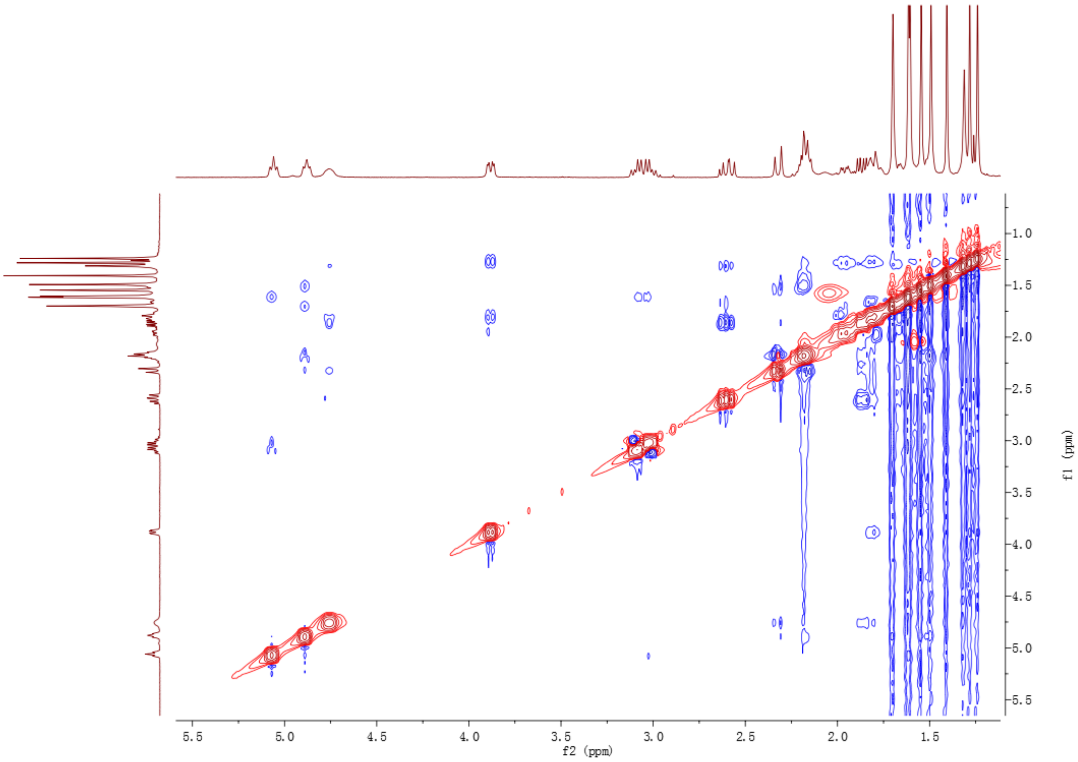

Supplement: Supplementary Information [file srep14772-s1.pdf]
